# Thermal Rates and High-Temperature Tunneling from Surface Reaction Dynamics and First-Principles

**DOI:** 10.1021/jacs.4c09017

**Published:** 2024-11-08

**Authors:** Florian Nitz, Liang Zhang, Nils Hertl, Igor Rahinov, Oihana Galparsoro, Alexander Kandratsenka, Theofanis N. Kitsopoulos, Daniel J. Auerbach, Hua Guo, Alec M. Wodtke, Dmitriy Borodin

**Affiliations:** † Institute for Physical Chemistry, Georg-August University of Goettingen, Tammannstraße 6, 37077 Goettingen, Germany; ‡‡ Department of Dynamics at Surfaces, Max Planck Institute for Multidisciplinary Sciences, Am Fassberg 11, 37077 Goettingen, Germany; § Department of Chemistry and Chemical Biology, Center for Computational Chemistry, University of New Mexico, Albuquerque, New Mexico 87131, United States; ∥ Department of Chemistry, University of Warwick, Gibett Hill Road, Coventry CV4 7AL, U.K.; ⊥ Department of Natural Sciences, The Open University of Israel, Raanana 4353701, Israel; # Donostia International Physics Center (DIPC), Paseo Manuel de Lardizabal 4, Donostia-San Sebastián 20018, Spain; ¶ Kimika Fakultatea, Euskal Herriko Unibertsitatea UPV/EHU, P.K. 1072 Donostia-San Sebastián 20018, Spain; ∇ School of Mathematics and Natural Sciences, University of Southern Mississippi, Hattiesburg, Mississippi 39406, United States; ○ International Center for Advanced Studies of Energy Conversion, Georg-August University of Goettingen, Tammannstraße 6, Goettingen 37077, Germany; ⧫ Center for Quantum Nanoscience (QNS), Institute for Basic Science (IBS), Seoul 03760, South Korea; †† Department of Physics, Ewha Womans University, Seoul 03760, South Korea

## Abstract

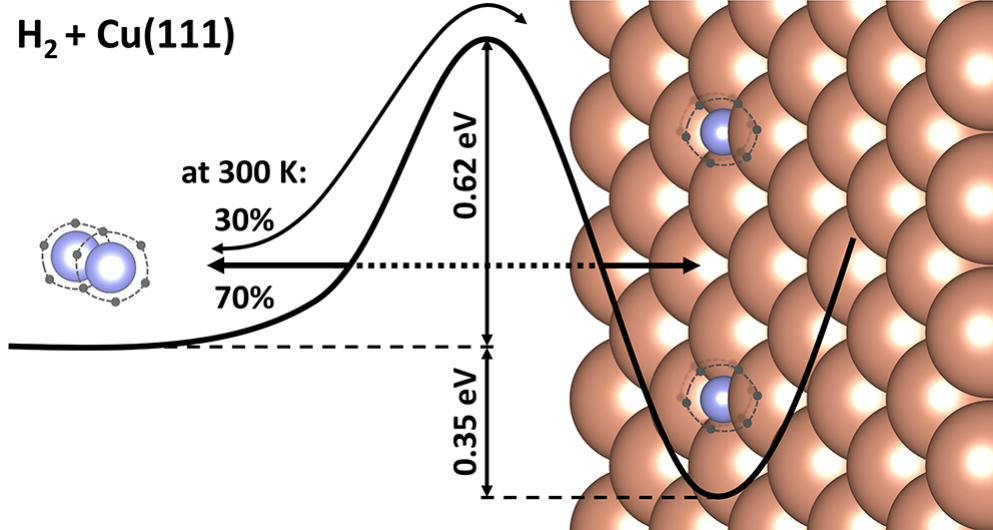

Studying dynamics of the dissociative adsorption and recombinative desorption of hydrogen on copper surfaces has shaped our atomic-scale understanding of surface chemistry, yet experimentally determining the thermal rates for these processes, which dictate the outcome of catalytic reactions, has been impossible so far. In this work, we determine the thermal rate constants for dissociative adsorption and recombinative desorption of hydrogen on Cu(111) between 200 and 1000 K using data from reaction dynamics experiments. Contrary to current understanding, our findings demonstrate the predominant role of quantum tunneling, even at temperatures as high as 400 K. We also provide precise values for the reaction barrier (0.619 ± 0.020 eV) and adsorption energy (0.348 ± 0.026 eV) for H_2_ on Cu(111). Remarkably, the thermal rate constants are in excellent agreement with a first-principles quantum rate theory based on a new implementation of ring polymer molecular dynamics for reactions on surfaces, paving the way to discovering better catalysts using reliable and efficient computational methods.

## 1 Introduction

Dissociative adsorption, the process by which a molecule breaks an internal bond while forming new bonds to atoms on a surface, is a critical step in heterogeneous catalysis as it produces an active form of a stable species.^1^ For example, hydrogen atoms bound on many metal surfaces are much more reactive than gas-phase hydrogen molecules. At the most fundamental level, this is how solid-state catalysts accelerate reactions that are normally too slow to be useful. The transition state of the dissociative adsorption reaction can be described as a species with partly broken internal bonds of the molecule along with only partly formed bonds to the surface atoms. This compromised electronic structure often means that the transition state is less stable than either reactants or products, i.e. the reaction possesses an activation barrier, which means high temperatures are required for the reaction to proceed rapidly. This explains why dissociative adsorption is often the slowest (rate-limiting) step in catalytic processes used in the industrial scale production of economically important chemicals. Well known examples include the dissociative adsorption of molecular nitrogen in the Haber-Bosch synthesis of ammonia,^2,3^ and the dissociative adsorption of methane^4^ in steam reforming used for industrial production of hydrogen. Understanding dissociative adsorption thus offers the prospect of enabling the development of predictive theories of surface chemistry that could guide efforts to improve important chemical processes in industry.

The challenge of achieving a quantitative fundamental understanding of dissociative adsorption has motivated the study of simple model systems which are both experimentally and theoretically tractable, a prime example being the thermally activated dissociative adsorption of hydrogen on copper surfaces. Since the first report of this reaction in 1843,^5^ our knowledge has been continually refined, spurred on by improving experimental capabilities and advances in theoretical methods. Today we have an extraordinary range of high-quality experimental data on the dynamics of this reaction including absolute dissociative adsorption probabilities as a function of the molecule’s vibrational temperature, incidence translation energy and incidence angle.^6−8^ Even more extensive and detailed experimental data are available on the reverse reaction, i.e. recombinative desorption, including angular and quantum-state resolved translational energy distributions of the desorbing products at a variety of surface temperatures.^8−12^ The quantitative consistency of the reported adsorption and desorption data, when considered within the principle of detailed balance,^8^ gives us great confidence in its intrinsic reliability. This treasure trove of detailed and reliable dynamical data has made the hydrogen dissociative adsorption reaction with copper an ideal test case for the development of theoretical methods for surface chemistry.^13^ As an example, theory has been able to come up with semiempirical density functional methods that when tuned to agree with molecular beam measurements of dissociative adsorption probabilities, accurately describe the barrier region of the potential energy surface (PES).^14^ This approach allowed molecular dynamics calculations to reproduce the probability of dissociative adsorption and its dependence on kinetic energy and vibrational state.^14,15^ With the application of the principle of detailed balance, a wide variety of data on the energy, angle, vibrational and rotational dependence of the associative desorption has also been successfully described.^16−18^ Due to these impressive successes, the adsorption barrier obtained with this approach is widely believed to be the most accurate value yet determined.

Despite this impressive progress, we do not have a validated first-principles theory for thermal rates of surface reactions, even for the extensively studied H_2_/Cu(111) model system. This is particularly unfortunate as thermal rates are by far the most commonly experienced manifestation of chemical transformations and provide information needed for practical applications. Thus, predictive rate theories are crucially important for the many cases where formidable instrumental difficulties make elementary rate constants inaccessible from an experiment.^19,20^

Several difficulties stand in the way of developing a predictive rate theory for surface chemistry in general and these challenges are exemplified by the hydrogen–copper benchmark system.

1 There are few reliable experimental measurements of thermal rates of surface reactions against which new theories might be tested; specifically, direct measurements of the dissociative adsorption rates of hydrogen on Cu(111) have proven so far impossible and reported values using indirect methods vary by a factor of 10^7^ at 300 K^8,21−23^—see Supporting Information Figure S1.
2 An accurate PES is a prerequisite for rate simulations, and it is typically not known with chemical accuracy. Even for the extensively studied system H_2_/Cu(111), there is a long-standing and still inconclusive discussion about a crucial feature of the PES—the dissociative adsorption energy—see Tables S1 and S2 and Figure S2 in the Supporting Information.
3 We do not have rate theories capable of rigorously accounting for the nuclear quantum effects expected for reactions involving light atoms.

In this work we show how to overcome these difficulties for the dissociative adsorption and recombinative desorption reactions of hydrogen on copper. First, we report highly accurate thermal rate constants over a wide temperature range (200–1000 K) derived from a plethora of existing reaction dynamics measurements. Specifically, we present a parametrized model describing the dissociative adsorption probability (sticking probability) for H_2_ and D_2_ interacting with a Cu(111) surface as a function of the incident molecule’s translational energy, ro-vibrational quantum state, incidence angle and the temperature of the copper surface. The parameters are optimized using a global fit to both adsorption and desorption data from previous experiments^6−12,21,24^ and then, to obtain thermal rates, the optimized sticking model is thermally averaged. In addition to the determination of the adsorption rate, we use previously reported^25−27^ temperature-programmed desorption (TPD) experiments and the principle of detailed balance to determine highly accurate thermal rate constants for recombinative desorption of H_2_ and D_2_. Based on this comprehensive experimental data, we are able to quantify the importance of tunneling in the thermal reactivity of hydrogen with Cu(111) and find that tunneling dominates the rate of adsorption and desorption even at temperatures as high as 380 K.

In addition, we use the experimentally derived thermal rate constants to determine accurate values for the dissociative adsorption barrier and adsorption energy (the energy released upon dissociative adsorption). This allows us to choose a density functional theory (DFT) exchange–correlation functional that accurately reproduces these values and thus can be used to calculate the PES needed as a starting point for first-principles calculations of thermal rates.

Finally, we introduce a new implementation of ring polymer molecular dynamics (RPMD) rate theory for chemical reactions on surfaces based on a PES constructed with this experimentally validated DFT functional and benchmark the RPMD predictions against the experimentally derived thermal rate constants. The use of an experimentally validated PES makes the comparison a test of the new rate theory, rather than an assessment of the accuracy of electronic structure theory. Although RPMD is known to accurately predict thermal rates of gas-phase reactions including nuclear quantum effects,^28^ this test is necessary as RPMD has not yet been applied to bimolecular reactions occurring on surfaces^29,45^ and there is no experimental validation of its ability to predict quantum effects in thermal surface chemistry. We find that the RPMD predictions are in excellent agreement with the experiment over the full temperature range for dissociative adsorption—a first-order process—and recombinative desorption—a second-order process. This represents, to the best of our knowledge, the most comprehensive and successful comparison of experimental thermal rates for a surface reaction with predictions of a first-principles thermal rate theory.

## 2 Results

**Figure 1:**
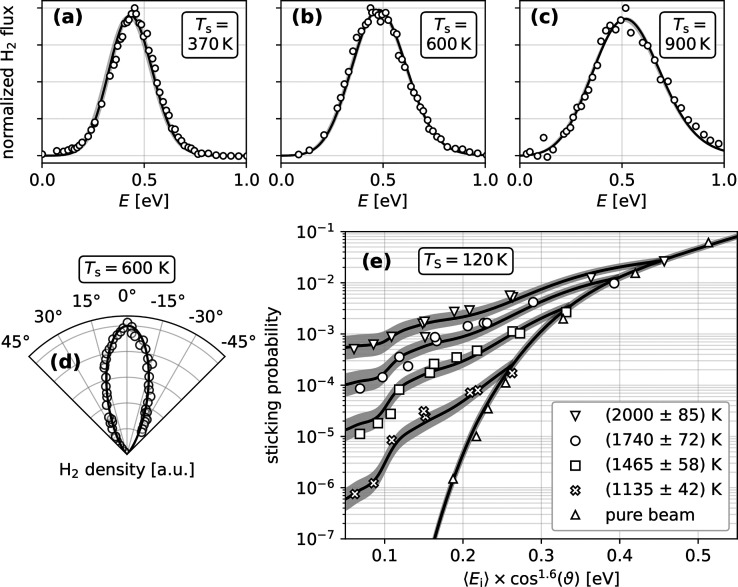
Experimental recombinative desorption dynamics and dissociative sticking probabilities for H_2_ compared to the fitted model. (a–c) Experimentally determined state resolved translational energy (*E*) distributions of H_2_ (*v* = 0, *J* = 1) desorbing from Cu(111) at various surface temperatures *T*_S_ (open circles).^11^ The solid line shows the fit achieved with the sticking model of this work. (d) Experimental angular density distribution of desorbing H_2_ from Cu(111) at *T*_S_ = 600 K (open circles) from ref (12) and fit achieved with the model of this work (solid line). (e) Hot nozzle data for H_2_ sticking on Cu(111) at *T*_S_ = 120 K. Experimental data (symbols) from ref (8) is compared to the fit (solid lines) achieved in this work. The uncertainty of the fit emerges from the uncertainty of the nozzle temperature, indicated by the gray shaded region. Data for (d,e) are not quantum state resolved, therefore model results were obtained from the Boltzmann average over the quantum states—see Supporting Information Section S2 for details of the analysis. An equivalent figure for D_2_ is shown in the Supporting Information (Figure S7).

Extending previous work,^8−11,16,21^ we develop a model which describes sticking probabilities *S*(*E_i_*,ϑ,*J*,*v*,*T*_s_) that depend on the hydrogen molecule’s incidence translational energy *E_i_* and angle ϑ, its rotational *J* and vibrational *v* quantum numbers,^6−12,21,24^ and the copper surface temperature *T*_S_(6,11)
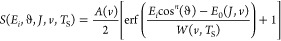
1*A*(*v*), *E*_0_, *n*, and *W*(*v*,*T*_S_) are parameters used to reproduce experimental observations. The term cos^*n*^(ϑ) is introduced to account for the fact that the component of incidence translational motion parallel to the surface is far less effective at promoting reaction than is the perpendicular component; *n* = 2 corresponds to “normal energy scaling”. A more detailed interpretation of this function and its parameters is given in ref (16). To determine the parameters of this model we performed a global fit over data of three types: absolute “hot nozzle” sticking probabilities,^7,8^ quantum-state resolved time-of-flight distributions of desorbing molecules^9,11^ and desorption angular distributions.^12^ We performed two global fits, one for data on H_2_ and another for D_2_. The fits give excellent agreement with the complete set of data, as shown for a representative subset in Figure 1 for H_2_, and for D_2_ in Figure S7 in the Supporting Information—see Section S2 for further details.

Using eq 1 with optimized parameters, we compute the incidence angle-averaged thermal sticking probability ⟨*S*⟩(*T*) and the thermal adsorption rate constant
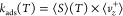
2where  is the thermal average velocity of gas-phase molecules (with mass *m*) toward the surface—see Supporting Information Section S3 for details. Results for *k*_ads_(*T*) will be denoted as “experimentally derived” in the following, as they have not been measured directly, but are derived from an experimentally parametrized model.

**Figure 2:**
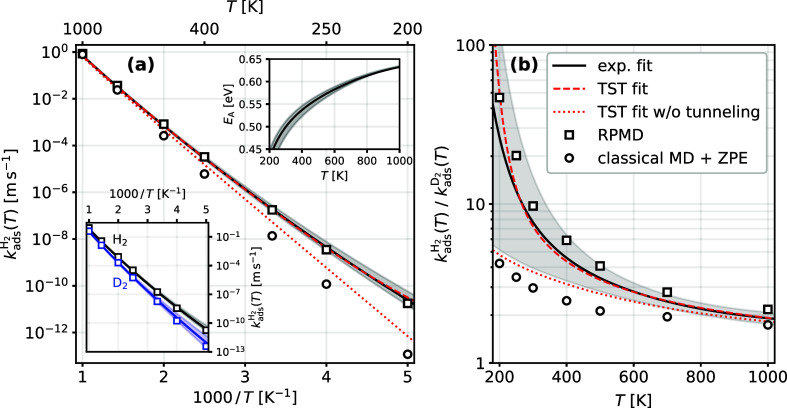
(a) Experimentally derived thermal adsorption rate constant *k*_ads_(*T*) and (b) Kinetic Isotope Effect (KIE) for dissociative hydrogen adsorption on Cu(111) (solid black lines). A TST model with recrossing and tunneling corrections is shown as red dashed lines. The TST model neglecting tunneling is shown as red dotted lines. Circles and squares are classical MD (1-bead RPMD) and quantum (converged RPMD) results, respectively, on a PES matching the experimental adsorption energy and barrier. 1-bead RPMD results were scaled to account for ZPE effects, which are not included in the simulation. The mismatch between circles and squares is indicative of quantum tunneling. The upper inset of panel (a) shows the Arrhenius activation energy of the adsorption rate constant for H_2_. The lower inset in (a) compares both H_2_ (black) and D_2_ (blue) adsorption rate constants with converged RPMD results (squares). The gray shaded regions indicate 65% confidence intervals for the experimentally derived results.

Figure 2a displays *k*_ads_(*T*) for H_2_ on Cu(111). Results for D_2_ are shown in the lower inset and in Figures S8 and S11 in Supporting Information. *k*_ads_(*T*) for H_2_ is markedly larger than that for D_2_ at all temperatures and this difference increases with decreasing temperature. This is shown more clearly in Figure 2b which displays the kinetic isotope effect (KIE); that is, the ratio of *k*_ads_(*T*) for H_2_ to D_2_. The growing KIE at reduced *T* reflects the fact that there is a curvature in the Arrhenius plots which is more pronounced for H_2_ than for D_2_. The upper inset of Figure 2a shows that for H_2_, the Arrhenius activation energy decreases by almost 0.2 eV as *T* goes from 1000 to 200 K. Taken together, these differences in the rates for H_2_ and D_2_ provide a strong indication^30,31^ that quantum tunneling plays an important role in this reaction.

Additional support for the importance of tunneling here be gained by transition state theory (TST) modeling. We compute the TST adsorption rate constant *k*_hTST_(*T*,ε_ads_^‡^) in the harmonic approximation based on DFT-derived transition state frequencies and apply three well-established ad hoc corrections. First, we account for trajectories that pass through the transition state and then return to the reactant state by multiplying the harmonic TST rate constant *k*_hTST_(*T*,ε_ads_^‡^) by a temperature-dependent recrossing correction factor κ(*T*) previously computed in ab initio molecular dynamics simulations.^32^ Second, we introduce a multiplicative temperature-independent correction factor *a* to account for anharmonicity of the nonreactive modes at the transition state. Third, we introduce a factor Γ(*T*,ν^‡^) to correct the TST model for tunneling. We compute Γ(*T*,ν^‡^) using a one-dimensional Eckart barrier model,^33−36^ which is parametrized by the imaginary frequency ν^‡^ of the transition state—see Supporting Information Section S4 for more details. In this case, no significant electronically nonadiabatic effects^37^ for H_2_ adsorption on Cu(111) are expected. In other cases, improved corrections to the TST rate expression may be required.

We performed a fit of this TST model to the H_2_ and D_2_ thermal adsorption rate constants simultaneously by adjusting the classical barrier ε_ads_^‡^ for adsorption, one anharmonicity correction for each isotopologue and H_2_’s imaginary transition state frequency. The corresponding imaginary frequency for D_2_ is not independently varied, rather it is scaled to the H_2_ value based on the reduced mass of the molecule. We find very good agreement of these fit results (red dashed lines in Figure 2) with rate constants derived from experiments (solid black lines) for both isotopologues over the entire temperature range.

To distinguish the influence of tunneling and ZPE on the adsorption rate constants, we set the tunneling correction Γ(*T*,ν^‡^) to unity, leaving only the ZPE effect in the model. The results are shown in Figure 2 as the red dotted lines. Clearly, tunneling is needed to explain the curvature of the rate constant and the increase of the KIE; neglecting tunneling degrades the fit over the entire temperature range.

The fit of the TST model also gives us an accurate determination of the classical barrier for hydrogen adsorption ε_ads_^‡^ = 0.619_-0.021_^+0.018^eV, where the error bars indicate the 95% confidence interval. Knowledge of ε_ads_^‡^ allows us to choose a semiempirical DFT functional, as was done in previous work using specific reaction parameter (SRP) functionals tuned to match experimental sticking probabilities for H_2_ on Cu(111).^13,14,17^ Here we use the PBEα-vdW functional^38,39^ with α set to 0.57, which was previously successfully applied to H_2_ adsorption on Cu(111)^40^ and predicts a barrier height of 0.607 eV. This allows us to evaluate a first-principles rate model of thermal surface chemistry with less concern that differences between its predictions and experiment arise from DFT related errors in the PES.

One of the motivations to obtain highly accurate experimentally derived thermal rate constants is to provide a basis for benchmarking first-principles quantum rate theories as they are developed. One such theory is RPMD. Extensively used for gas-phase reactions,^41−44^ RPMD has recently been extended to the calculation of dissociative adsorption rates on transition metal surfaces.^45^ RPMD simulates thermal rates, accounting for nuclear quantum effects (tunneling and zero-point energy)^28^ by mapping a quantum statistical property of a particle to that of a “necklace” of multiple harmonically connected beads.^46^ Through simulations of classical trajectories of such “ring polymers”, RPMD yields approximate quantum rate constants with far less computational complexity and better scaling than conventional quantum dynamics methods.^28,41^ Furthermore, it provides a natural framework for considering dynamical recrossing, which is challenging to include in TST. For precise quantum mechanical results, the number of beads in the simulation is increased until convergence is reached, while the classical result for the same system can be obtained simply by setting the number of beads to one.^47^ Hence, the comparison of converged and single-bead RPMD calculations, henceforth referred to as classical MD, provides a convenient way to determine the contribution of nuclear quantum effects to a given rate constant. Here, the RPMD calculations were performed for a rigid Cu(111) surface using a new permutation invariant polynomial-neural network (PIP-NN)^48^ PES based on energies from the PBEα-vdW(α = 0.57) functional,^38,39^ slightly modified (by shifts of <20 meV) to match the experimental dissociation barrier and adsorption energy derived in this work. Our new implementation of RPMD for on-surface reactions required several important modifications of the RPMD rate theory for gas-phase bimolecular reactions, including the definition of the initial dividing surface for two adsorbed reactants and the use of a two-dimensional free gas approximation for the reactants. The approximations we have chosen here may not be applicable to all systems, particularly not to those where the diffusion barrier is large or when surface atoms are significantly involved in the reaction. For further details on the RPMD rate theory see Supporting Information Section S5.

Figure 2 also includes the RPMD predictions of thermal dissociative adsorption rate constants. The converged, quantum results, shown as squares, are in excellent agreement with the experimentally derived rate constants. ZPE corrected classical calculations are shown as circles and were obtained by multiplying classical MD rate constants by exp(−ΔZPE/*k*_B_*T*), where ΔZPE is the difference between zero-point energies of the transition state and the gas-phase molecule. These results fail to reproduce the experimental observations and the deviation is largest at low temperatures. This is additional strong evidence that quantum tunneling is important in this reaction.

So far, we have been discussing the process of dissociative adsorption. We now extend the discussion to the determination of the rate constant for the reverse process, recombinative desorption, *k*_des_(*T*), by application of the detailed balance rate model (DBRM):^49−51^
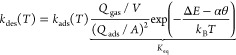
3where Δ*E* = Δε + ZPE_gas_ – 2 ZPE_ads_. This equation is easily understood by recognizing the statistical mechanical expression for the equilibrium constant *K*_eq_, which is computed from normalized partition functions for the gas-phase molecule *Q*_gas_ and the adsorbed hydrogen/deuterium atoms *Q*_ads_. We have shown in previous work^49^ how *Q*_ads_ can be computed from an explicit counting of nuclear eigenstates obtained by solving the nuclear Schrödinger equation on a DFT-derived H/D-Cu(111) interaction potential. We follow that procedure here—see Supporting Information Section S6.1 for more details. This procedure also yields ZPEs of the adsorbates, ZPE_ads_. Together with the experimental ZPE of the gas-phase molecule, ZPE_gas_, we introduce the isotope-independent, classical adsorption energy Δε. The parameter α accounts for the coverage dependence of the adsorption energy. Note that *k*_des_(*T*) has units of m^2^ s^–1^, or alternatively, after multiplying with the Cu atom density per unit area (1.77 × 10^19^ m^–2^)^26^, ML^–1^ s^–1^.

**Figure 3:**
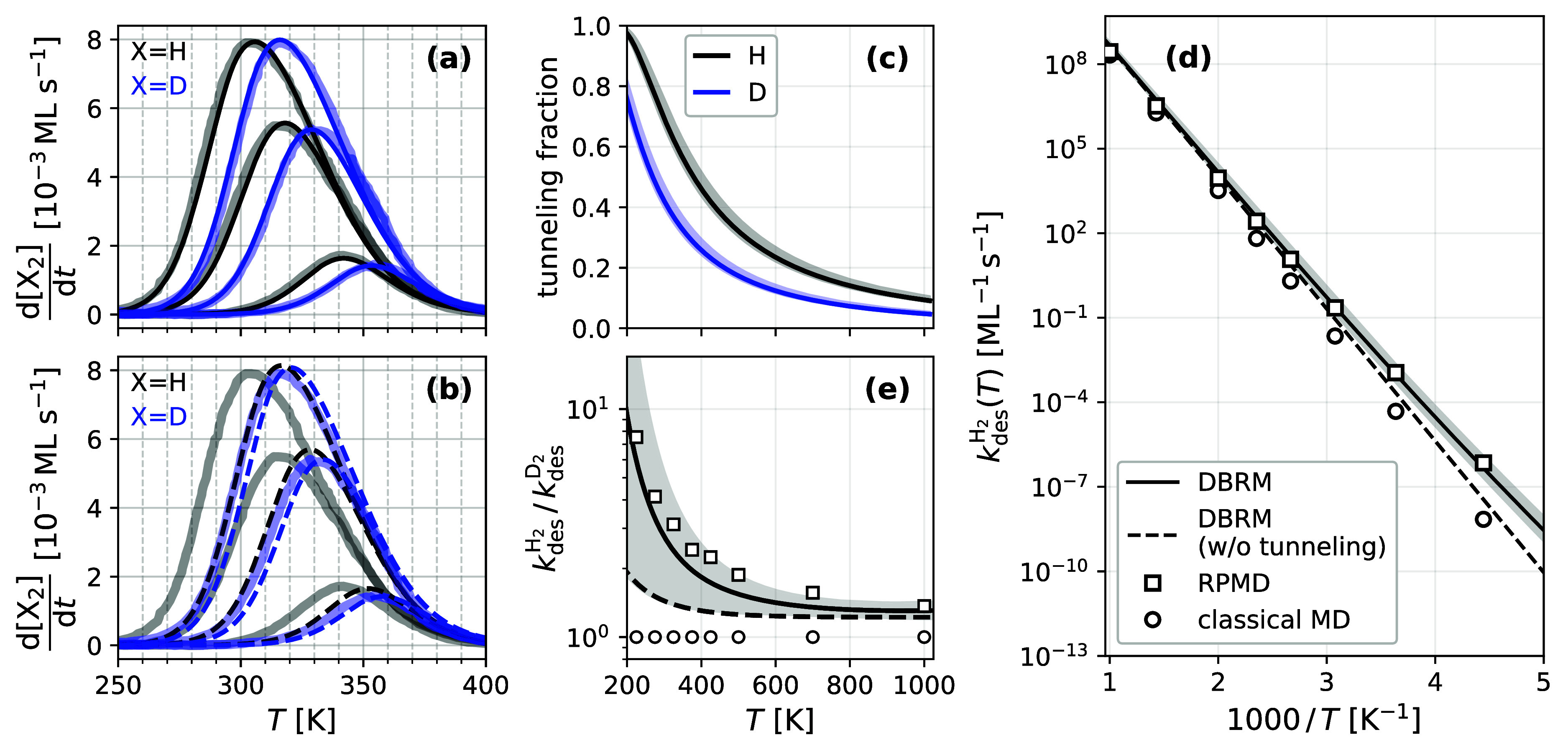
(a) Experimental^26^ TPD spectra for H_2_ (black) and D_2_ (blue) at three initial coverages (0.05, 0.19, 0.30 ML, with increasing amplitudes) shown as thick transparent lines. The thin solid lines are based on the DBRM—eq 3—and values of *k*_ads_(*T*) from Figure 2. (b) shows the same lines as (a) but with a version of the DBRM neglecting tunneling (dashed lines). (c) Fraction of the desorption rate constant arising from tunneling, shown for H_2_ (black) and D_2_ (blue). (d) Thermal recombinative desorption rate constants for H_2_ (black solid line) at zero H atom coverage derived from a fit of eq 3 to TPD data. Also shown are converged RPMD rate constants (open squares) and rate constants from classical MD (1-bead RPMD, circles). The black dashed line shows a version of eq 3 neglecting tunneling. (e) KIE for the recombinative desorption rate constant; symbols and lines are the same as in (d).

We optimized the values of Δε and α in order to fit simultaneously TPD data for H_2_ and D_2_ desorption from three independent studies^25−27^ involving 25 TPD curves at different coverages and heating rates. We find good agreement with all data when setting the classical adsorption energy to Δε = 0.348_–0.023_^+0.028^ eV and its linear dependence on surface coverage to α = 0.208_–0.072_^+0.049^ eV ML^–1^. From these results we can also determine the classical barrier height for desorption, ε_des_^‡^ = ε_ads_^‡^ + Δε = 0.967_–0.031_^+0.033^ eV. Figure 3a shows a fit of eq 3 to a representative subset of the experimental data for H_2_ and D_2_, each at three distinct initial coverages reproduced from ref (26). For further details see Section S6.2 and Figure S21 in Supporting Information.

We note that there is a large KIE in the TPD data depicted in Figure 3a, which is quantitatively reproduced by the DBRM. Since eq 3 was constructed from the principle of detailed balance and because it relies on the experimentally derived adsorption rate constants *k*_ads_(*T*), the recombinative desorption rate constants *k*_des_(*T*) also includes strong tunneling contributions. If we replace *k*_ads_(*T*) in eq 3 with adsorption rate constants that neglect tunneling (see above), we obtain the black and blue dashed TPD spectra of Figure 3b. These fail to reproduce experiment and the isotope effect is almost completely absent. Thus, we conclude that the large isotope effect seen in TPD originates primarily from quantum tunneling.

Figure 3c shows the relative contribution of tunneling to the adsorption and desorption rate constants, the tunneling fraction, defined as 1–1/Γ(*T*,ν^‡^), where Γ(*T*,ν^‡^) is the fitted Eckart tunneling correction (see above and Section S4 in the Supporting Information). Tunneling is responsible for more than 50% of the thermal reactivity of H_2_ on Cu(111) up to temperatures as high as 380_–13_^+29^ K, which is actually higher than the peak temperature of TPD spectra.

We also used our PIP-NN PES to compute thermal rate constants for recombinative H_2_ and D_2_ desorption with RPMD, see Section S5 in the Supporting Information. As discussed in ref (49), neglecting the electronic spin degeneracy for this reaction would lead to a 4-fold overestimation of the recombination rate constants, therefore all results of the RPMD theory presented below have been divided by 4 to account for this effect in an a posteriori manner. An additional division by 2 was introduced to account for the indistinguishability of the two atoms in the recombination reaction. In Figure 3d we show converged, quantum RPMD recombination rate constants as open squares in comparison to experimentally derived rate constants (solid lines). The agreement between experiment and quantum RPMD is excellent, within a factor 1.7 for H_2_ over the entire temperature range. The results of the classical MD rate calculations are shown as open circles in Figure 3d; the agreement to the experiment substantially deteriorates due to the absence of tunneling. Figure 3e shows the KIE of the recombination rate constant, again demonstrating the importance of tunneling in this system.

## 3 Discussion

The basis of the work presented here is a global fit of previously reported reaction dynamics data, from which we derive thermal rate constants for H_2_ and D_2_ adsorption on Cu(111). Due to the low thermal reaction probability, no direct measurements of these rate constants are available. There have been previous attempts to extract the rate constant from reaction dynamics data^8,21−23^ however the results vary widely; for example, there is a spread of 7 orders of magnitude in the rates for adsorption at 300 K (see Figure S1 in the Supporting Information). In contrast to previous work, this study takes a far wider range of reaction dynamics data into account, includes more recent experiments, and successfully performs a global fitting of a sticking probability model that, once optimized, can be thermally averaged to obtain thermal rate constants. By using the DBRM, we also derive rate constants for the reverse process—recombinative desorption—that perfectly reproduce TPD data. This leads us to believe that both the adsorption and desorption rate constants of this work are far more accurate than any previous reports.

We also presented the first experimental evidence of quantum tunneling in this reaction, which was suggested from theory previously.^23^ This evidence includes: curvature in the Arrhenius plots of adsorption rates that is larger for H_2_ than for D_2_; an increase in this curvature with decreasing temperature; and a large isotope effect in both the thermal rate constants and peak desorption temperatures in TPD that cannot be explained from differences in ZPE alone. We are able to unambiguously evaluate the contribution of tunneling to the adsorption and desorption reactions of H_2_ on Cu(111) and find that it is dominant even at high temperatures (50% contribution at 380_–13_^+29^ K).

Our results support prior claims that tunneling can dominate chemical reactivity even far above room temperature, for example in methane dissociation on tungsten^52,53^ and even for complex condensed phase systems such as enzymes.^54,55^ However, these studies could not quantify the contribution of tunneling to reaction rates experimentally.

Our first-principles rate simulations required an accurate PES. The state of the art in computational heterogeneous catalysis research uses PESs based on DFT at the generalized gradient approximation level,^3,19,56,57^ which unfortunately gives a large variation of the adsorption energy and barrier depending on the choice of the exchange–correlation functional (see Supporting Information Table S2). Our TST and detailed balance-based analysis yields accurate values for these two critical features of the PES derived from experiments, and we used these to select an accurate functional for constructing the PES underlying the rate simulation. We chose the semiempirical PBEα-vdW functional (with α = 0.57^40^) as its predictions of the dissociative adsorption barrier height (0.607 eV) and also the adsorption energy (0.227 eV at an H atom coverage of 0.5 ML) are in good agreement to the experimentally derived results—0.619_–0.021_^+0.018^ and 0.244_–0.034_^+0.046^ eV—see also Supporting Information Figure S24.

It is interesting to compare these results with predictions of other commonly used functionals, see Figure S26 and Section S7 in the Supporting Information. While most functionals yield predictions that are in poor agreement with the experiment, the semiempirical functionals SRP48^16,17^ and PBEα-vdW (α = 0.57)^40^ give adsorption energies and barriers extraordinarily close to the values derived in this work. The agreement for the barrier height is not surprising since, for example, SRP48 was tuned to reproduce experimental adsorption probabilities of H_2_ on Cu(111). The agreement with the experimental adsorption energy is less obvious and indeed it was pointed out that it might be difficult to reproduce both the adsorption energy and barrier with the same functional.^58^ We see in this work that this is not the case for the system investigated here, and we feel that it is likely to be valid for all reactions dominated by a single electronic state. In Figure S26 in the Supporting Information we show that adsorption energies and barriers calculated with various DFT functionals are correlated in a manner reminiscent of the Brønsted-Evans–Polanyi relationship^59^ and that tuning a functional so it correctly predicts one of these quantities also leads to an accurate prediction of the other. This is true for both the SRP48 and PBE α-vdW functional. This correlation suggests that there is a broad applicability of semiempirical functionals for computational catalysis.

We calculated rate constants for dissociative adsorption and recombinative desorption with RPMD and compared the results to the accurate experimentally derived thermal rates determined in this work. For H_2_ adsorption and desorption we find agreement within a factor of 1.7 over the entire temperature range of this work—200 to 1000 K. For D_2_ deviations are similar (within a factor of 2.4). This agreement between RPMD and experiment is outstanding, especially when considering that the rate constants vary by 15 orders of magnitude over this temperature range.

The overall good agreement shows that RPMD is a predictive method for surface reactions involving nuclear quantum effects at a computational cost and scaling far less severe than full quantum dynamics simulations. Moreover, RPMD provides a more rigorous framework to account for dynamical recrossing, anharmonicity and quantum effects than does the ad hoc treatment of these factors within the TST framework. This is particularly important because we have shown incontrovertible evidence that H atom tunneling is important in surface chemical reactions at surprisingly high temperatures.

As stated in the introduction, the system studied here is a particularly favorable one for testing theoretical models. Clearly RPMD must be tested in the future for a wide variety of systems to assess to what extent the approximations we used here will work for other systems in heterogeneous catalysis. Nevertheless, the ability of RPMD to predict thermal rates with such high accuracy demonstrated here offers the prospect of accurately treating realistic problems in heterogeneous catalysis, electrocatalysis, and materials processing.

## Supporting Information Available

Analysis of experimental sticking coefficient data, calculation of thermal adsorption rate constants, details of the TST model, construction of the neural network PES for adsorption and desorption, details of the ring polymer molecular dynamics rate theory, construction of the model used to fit experimental TPD data, computational details, comparison to the literature and additional references (PDF)
