# Supplementary material for: Thermal Rates and High-Temperature Tunneling from Surface Reaction Dynamics and First-Principles
Source: J Am Chem Soc. 2024 Nov 8;146(46):31538–46. doi: 10.1021/jacs.4c09017 (PMC11583301; doi:10.1021/jacs.4c09017)
Supplement: Supplementary file 1 — ja4c09017_si_001.pdf [file ja4c09017_si_001.pdf]

Thermal Rates and High-Temperature Tunneling from Surface Reaction Dynamics and First-Principles

Florian Nitz[1,2]†, Liang Zhang[3]†, Nils Hertl[4], Igor Rahinov[5], Oihana Galparsoro[6,7], Alexander Kandratsenka[2], Theofanis N. Kitsopoulos[2,8], Daniel J. Auerbach[2], Hua Guo[3]*, Alec M. Wodtke[1,2,9]**, Dmitriy Borodin[2,10,11]***

[1] Institute for Physical Chemistry, Georg-August University of Goettingen, Tammannstraße 6, 37077 Goettingen, Germany

[2] Department of Dynamics at Surfaces, Max Planck Institute for Multidisciplinary Sciences, Am Fassberg 11, 37077 Goettingen, Germany

[3] Department of Chemistry and Chemical Biology, Center for Computational Chemistry, University of New Mexico, Albuquerque, New Mexico 87131, United States

[4] Department of Chemistry, University of Warwick, Gibett Hill Road, Coventry CV4 7AL, U. K.

[5] Department of Natural Sciences, The Open University of Israel, Raanana 4353701, Israel

[6] Donostia International Physics Center (DIPC), Paseo Manuel de Lardizabal 4, Donostia-San Sebastián 20018, Spain

[7] Kimika Fakultatea, Euskal Herriko Unibertsitatea UPV/EHU, P.K. 1072 Donostia-San Sebastián 20018, Spain

[8] School of Mathematics and Natural Sciences, University of Southern Mississippi, Hattiesburg, Mississippi 39406, United States

[9] International Center for Advanced Studies of Energy Conversion, Georg-August University of Goettingen, Tammannstraße 6, Goettingen 37077, Germany

[10] Center for Quantum Nanoscience (QNS), Institute for Basic Science (IBS), Seoul 03760, South Korea

[11] Department of Physics, Ewha Womans University, Seoul 03760, South Korea

Email: *hguo@unm.edu, **alec.wodtke@mpinat.mpg.de, ***borodin.dmitriy@qns.science

† These authors contributed equally to this work.

Contents

S1. Literature Values for Adsorption Rate Constants, Adsorption Barriers and Energies 3

S1.1. Adsorption Rate Constants 3

S1.2. Adsorption Barriers and Adsorption Energies 4

S2. Re-Analysis of Sticking Probability Measurements 8

S2.1. Fit to Absolute Adsorption Data 8

S2.2. Quantum-State Resolved Kinetic Energy Distributions 17

S2.3. Angular Density Distributions of Desorbing H_2 and D_2 21

S2.4. Additional Figures on Sticking Probability Measurements 23

S3. Thermal Sticking Coefficient and Adsorption Rate Constant 24

S4. Adsorption Rate Constant Modeling using Transition State Theory 27

S5. Adsorption and Desorption Rate Constants from Ring Polymer Molecular Dynamics 32

S5.1. Six-Dimensional Potential Energy Surface 32

S5.2. Ring Polymer Molecular Dynamics Rate Theory 33

S5.3. Derivation of Equation (S35) 44

S6. Determination of the Dissociative Adsorption Energy 47

S6.1. Adsorbate Partition Function Modeling 47

S6.2. Fits to Temperature Programmed Desorption Data 50

S6.3. Estimation of the Adsorption Energy from Eley-Rideal Reactions 54

S6.4. Previous Determinations of the Adsorption Energy 56

S7. Density Functional Theory (DFT) Calculations 58

S7.1. Hydrogen Dissociation: Adsorption Barrier and Energy 58

S7.2. Calculation of the in-plane Interaction Potential 64

S8. Additional References 65

S1. Literature Values for Adsorption Rate Constants, Adsorption Barriers and Energies

S1.1. Adsorption Rate Constants

Figure S1 shows previous reports of the thermal rate constant $k_{\mathrm{ads}}(T)$ for hydrogen and deuterium adsorption on Cu(111). Here, "thermal rate constant" refers to an average of the quantum state and kinetic energy specific microcanonical rate constants weighted by the Maxwell-Boltzmann populations of these states at the temperature T [1].

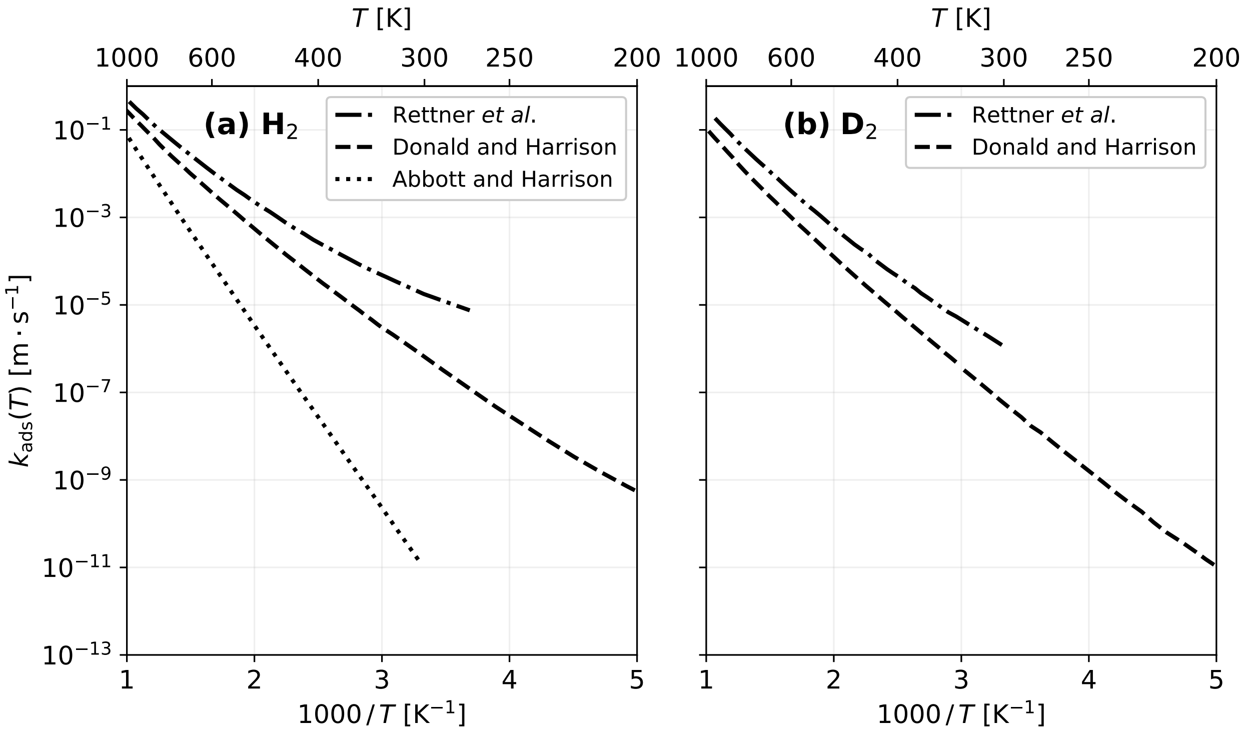

Figure S1: Thermal rate constants for dissociative adsorption of hydrogen—panel (a)—and deuterium—panel (b)—on Cu(111), taken from Rettner et al. [2,3], Donald and Harrison [4] and Abbott and Harrison [5]. Note: The thermal adsorption rate constant can be determined by multiplying the thermal sticking coefficient with the thermal average velocity $\langle v_z^+ \rangle$ along one (positive) direction of space $\langle v_z^+ \rangle = \sqrt{k_B T/(2\pi m)}$, see also section S3.

S1.2. Adsorption Barriers and Adsorption Energies

In Table S1 and Table S2 below we list barrier heights and adsorption energies for dissociative adsorption of a hydrogen molecule at copper surfaces. We define the adsorption barrier height as the energy of the transition state minus the energy of the H_2 molecule in the gas phase. The adsorption energy is defined as the energy of the H_2 gas-phase molecule minus the energy of two surface-adsorbed hydrogen atoms. Therefore, the adsorption energy is positive if energy is released upon dissociative adsorption of a gas-phase hydrogen molecule to form two hydrogen atoms.

Table S1 shows previously reported experimental values for the activation energy of hydrogen adsorption (and adsorption barriers italicized) and heats of hydrogen adsorption (and adsorption energies italicized).

Table S2 shows classical dissociative adsorption energies and classical barrier heights for hydrogen adsorption on Cu(111) obtained with density functional theory (DFT), semi-empirical methods and diffusion Monte-Carlo. DFT values include calculations based on the local density approximation (LDA), generalized gradient approximation (GGA), meta-GGA with various exchange-correlation functionals, as well as random phase approximation (RPA).

Figure S2 is a visual representation of Table S1 and Table S2. For this figure, 26 meV (37 meV) was subtracted from all DFT computed classical barrier heights (adsorption energies) to yield ZPE corrected values for hydrogen. These corrections are based on results from this work (see sections S4 and S6.2) and ensure comparability to experimental results from Table S1.

Table S1: Previously reported experimental activation energies for adsorption (or adsorption barriers, italic) and heats of adsorption (or adsorption energies, italic) for dissociative adsorption of hydrogen on various copper surfaces.

**reference**	**year**	**value [eV]**	**material**
activation energies for adsorption / *adsorption barriers*			
Kwan *et al.*[6]	1951	0.890	reduced copper
Mikovsky *et al.* [7]	1954	1.003	copper foil
Cadenhead, Wagner [8]	1971	0.738	electro-reduced $Cu(NO_3)_2$
Alexander, Pritchard [9]	1972	0.363	copper films
Kiyomiya *et al.* [10]	1974	0.391	copper powder
Balooch, Stickney [11]	1974	0.260	Cu(111)
Balooch, Stickney [11]	1974	0.087	Cu(110)
Balooch, Stickney [11]	1974	0.217	Cu(100)
Hayden, Lamount [12]	1989	1.000	Cu(110)
Campbell, Campbell [13]	1991	0.621	Cu(110)
Hayden, Lamount [14]	1991	0.650	Cu(110)
Sandoval, Bell [15]	1993	0.454	silica-supported copper
Rasmussen *et al.* [16]	1993	0.497	Cu(100)
Rettner et al. [2]	1995	0.390	Cu(111)
Tabatabaei *et al.* [17]	1999	0.435	polycrystalline copper
Donald, Harrison [4]	*2014*	*0.450*	*Cu(111)*
heats of adsorption / *adsorption energies*			
Ward [18]	1931	0.391	reduced copper oxide
Beebe [19]	1932	0.535	reduced copper oxide
Beebe *et al.* [20]	1935	0.475	reduced copper oxide
Shield, Russell [21]	1960	0.434	material not specified
Holden, Rossington [22]	1965	0.333	copper films
Alexander, Pritchard [9]	1972	0.467	copper films
Kiyomiya *et al.* [10]	1974	0.399	copper powder
Campbell, Campbell [13]	1991	0.056	Cu(110)
Sandoval, Bell [15]	1993	0.438	silica-supported copper
Reisfeld *et al.* [23]	1995	0.580	Cu(111)
Tabatabaei *et al.* [17]	1999	0.269	polycrystalline copper
Cao et al. [24]	*2018*	*0.152*	*Cu(111)*

Table S2: Classical dissociative barrier heights and classical energies for hydrogen adsorption on Cu(111) obtained with DFT, semi-empirical methods and diffusion Monte-Carlo. For calculational details on values from this work, see section S7.1.

reference	energy value [eV]	H coverage [ML]	method
dissociative barrier heights			
Wei *et al.* [25]	0.307	1/2	GGA (PBE+D3BJ)
Wei *et al.* [25]	0.587	1/2	RPA
Hammer *et al.* [26]	0.540	2/3	GGA (PW91)
Diaz *et al.* [27]	0.628	1/2	SRP43*
Nattino *et al.* [28]	0.628	1/2	SRP48*
Tchakoua *et al.* [29]	0.478	2/9	GGA (PBE)
Tchakoua *et al.* [29]	0.762	2/9	GGA (RPBE)
Tchakoua *et al.* [29]	0.618	2/9	GGA (SRP50)
Tchakoua *et al.* [29]	1.026	2/9	GGA (vdW-DF1)
Tchakoua *et al.* [29]	1.144	2/9	GGA (vdW-DF2)
Tchakoua *et al.* [29]	0.889	2/9	GGA (PBE-vdW-DF2)
Tchakoua *et al.* [29]	0.928	2/9	GGA (BEEF-vdW-DF2)
Tchakoua *et al.* [29]	0.667	2/9	GGA (revTPSS)
Tchakoua *et al.* [29]	0.382	2/9	meta-GGA (SCAN)
Tchakoua *et al.* [29]	0.647	2/9	meta-GGA (MS-B86bl)
Tchakoua *et al.* [29]	0.378	2/9	meta-GGA (MS2)
Tchakoua *et al.* [30]	0.617	2/9	GGA (PBEα-vdW,α=0.57)
Doblhoff-Dier *et al.* [31]	0.559	**	diffusion Monte-Carlo
this work	0.292	1/2	GGA (PBE-TS)
this work	0.440	1/2	GGA (PBE)
this work	0.468	1/2	GGA (optB86b)
this work	0.563	1/2	GGA (RPBE:PBE=43:57)
this work	0.578	1/2	GGA (SRP48)
this work	0.584	1/2	GGA (SRP50)
this work	0.730	1/2	GGA (RPBE)
this work	1.122	1/2	GGA (vdW-DF2)
this work	0.607	1/2	GGA (PBEα-vdW,α=0.57)
adsorption energies			
Wei *et al.* [25]	0.243	1/2	GGA (PBE+D3BJ)
Wei *et al.* [25]	0.003	1/2	RPA
Hammer *et al.* [26]	-0.130	2/3	GGA (PW91)
Hammer *et al.* [26]	0.750	2/3	LDA
Mudiyanselage *et al.* [32]	0.567	3/4	GGA (PW91)
Sakong, Groß [33]	0.176	1/4	GGA (PW91)

this work	0.511	1/2	GGA (PBE-TS)
this work	0.386	1/2	GGA (PBE)
this work	0.365	1/2	GGA (optB86b)
this work	0.257	1/2	GGA (RPBE:PBE=43:57)
this work	0.242	1/2	GGA (SRP48)
this work	0.236	1/2	GGA (SRP50)
this work	0.088	1/2	GGA (RPBE)
this work	-0.198	1/2	GGA (vdW-DF2)
this work	0.227	1/2	GGA (PBEα-vdW,α=0.57)

* this value was obtained semi-empirically
** extrapolated to infinite system size [31]

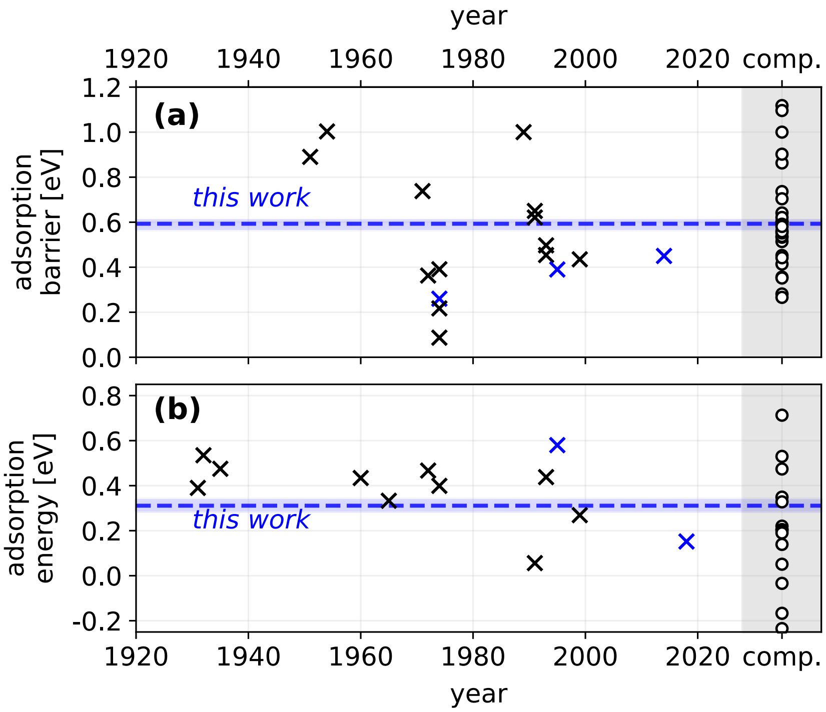

Figure S2: (a) Visual representation of previously reported experimental adsorption barrier heights (crosses) compared to experimental results from this work (dashed line) over the past 100 years. Blue and black symbols indicate values for Cu(111) and other copper surfaces, respectively. The transparent region around the dashed line indicates experimental uncertainties from this work. Barrier heights obtained with computational methods such as Density Functional Theory, semi-empirical methods and diffusion Monte-Carlo are shown as circles on the right side. (b) Same as (a), but for the adsorption energy. To all DFT values a zero-point energy correction was applied as described in the text. For references and more details, see Table S1 & Table S2.

S2. Re-Analysis of Sticking Probability Measurements

The dynamical representation of the sticking probability of H_2 and D_2 on copper is expressed by equation (1) of the main text and reproduced here.

$$S(E,\vartheta,J,v,T_S) = \frac{A(v)}{2}\left[\mathrm{erf}\left(\frac{E-E_0(J,v)}{W(v,T_S)}\right)+1\right] \quad (S1)$$

Here, E is related to the molecule's incidence translational energy E_i by the equation $E = E_i\cos^n(\vartheta)$ if molecules impinge under the angle ϑ to the surface normal. E_0 reflects the characteristic kinetic energy around which the sticking curve S is centered, W represents the steepness of the sticking probability with increasing E and A represents the asymptotic sticking probability at high E. The parameter n describes relative effectiveness of the molecule's normal component of motion to promote adsorption. If $n = 2$, only the normal component promotes adsorption, whereas if $n = 0$ normal energy and parallel energy are equally effective. Consistent with previous work [2,34], we assume that: E_0 values depend on both rotational J and vibrational v quantum numbers but are independent of surface temperature T_S; W-values are dependent only on v and T_S and; $A(v)$ values are only dependent upon v. Hence S are threshold like curves that depend on E, ϑ, J, v, & T_S. E_0 and W-values used in this work were taken from a recent study, Kaufmann *et al.* [34], where $T_S = 923$ K. In section S2.1 we determine the values for A from sticking probability data, where $T_S = 120$ K. To handle the information available from these two experiments consistently in our analysis, we assume a simple dependence of W-values on T_S.

$$W(v,T_S = 120\ \mathrm{K}) = \delta_W(v)\times W(v,T_S = 923\ \mathrm{K}) \quad (S2)$$

S2.1. Fit to Absolute Adsorption Data

In the work of references 2,3,35-37, sticking probabilities were measured using hot nozzle supersonic beams of pure and anti-seeded H_2 or D_2 (in different rare gases) at various incidence angles ϑ and molecular beam nozzle temperatures T_n between room temperature and 2100 K. Variation of the nozzle temperature influences both the incidence translational energy of molecules colliding with the surface and their population in higher rotational and vibrational quantum states. Such high nozzle temperatures require introducing sticking contributions from H_2 ($v = 2$) and D_2 ($v = 3$) to achieve a good fit to the data. Although these states do not directly contribute to the thermal adsorption rate constant for the temperature range of interest in this study (200 K to 1000 K), we found that without them, ground-state sticking curves were distorted. $E_0(v,J)$ and $W(v)$ parameters for H_2 ($v = 2$) and D_2 ($v = 3$) were not determined experimentally by Kaufmann *et al.* [34] and must therefore be found in another way. For H_2, $W(v = 2, T_S = 923\ \mathrm{K})$ was estimated by linear extrapolation from Kaufmann's values for $v = 0$ & 1. In a similar way, we used the vibrational efficacy for $v = 1$ to extrapolate all values $E_0(J, v = 1)$ to values of $E_0'(J, v = 2)$. This is an approximation as the vibrational efficacy for $v = 2$ is not necessarily the same as that of $v = 1$, but the vibrational efficacy of $v = 2$ has not been measured previously [34]. We therefore correct

for this approximation by scaling resulting $E_0'(J, v = 2)$ with an additional fit parameter δ_{E_0} to yield $E_0(J, v = 2)$, which is then inserted into equation (S1).

$$E_0(J, v = 2) = \delta_{E_0} \times E_0'(J, v = 2) \tag{S3}$$

For D_2, a similar extrapolation based on vibrational efficacy leads to unphysical results. Therefore, we made $E_0(J, v = 3)$ an additional fitting assuming the same value for all J-states, similar to previous analysis of reference 35. As before, $W(v = 3, T_S = 923\ \mathrm{K})$ is linearly extrapolated based on known $W(v, T_S = 923\ \mathrm{K})$ values for $v = 0, 1$ & 2.

Since the molecular beam expansion results in a distribution of translational energies $g(E)$ around a mean value $\langle E \rangle$, the sticking coefficient $S(E, J, v, T_s)$ must be averaged over $g(E)$ to obtain the experimentally derived molecular sticking probability $\bar{S}_{\mathrm{mol}}(\langle E \rangle, T_n)$; see the integrals in equation (S4).

$$\bar{S}_{\mathrm{mol}}(\langle E \rangle, T_n) = \sum_{J=0} \sum_{v=0} P_{\mathrm{Boltzm.}}(J, v, T_n) \frac{\int_0^\infty S(E, J, v, T_S) \times g(E)\, \mathrm{d}E}{\int_0^\infty g(E)\, \mathrm{d}E} \tag{S4}$$

We weight these averages for each pair of quantum numbers (J, v) by their Boltzmann population $P_{\mathrm{Boltzm.}}(J, v, T_n)$ at the nozzle temperature T_n. Rotational state distributions of the incident molecules are known to follow Boltzmann distributions with a rotational temperature T_{rot} that is between 80% and 100% of T_n [2]. We therefore assume a thermal population of rotational states in our modelling where $T_{\mathrm{rot}} = 0.9\ T_n$. Vibrational state populations are assumed to be thermalized at T_n; vibrational relaxation in the expansion was found to be negligible [2,36]. The summation in equation (S3) is performed up to $v = 2$ for H_2 and $v = 3$ for D_2, and for all respective J-states for which E_0 parameters were reported by or are predictable from Kaufmann *et al.* The beam translational energy distribution is approximated as a Gaussian function with a full width at half maximum of $0.15\ \langle E \rangle$ [38].

$$g(E) = \frac{1}{\sqrt{2\pi\sigma^2}} \exp\left(-\frac{(E - \langle E \rangle)^2}{2\sigma^2}\right) \quad \text{with:} \quad \sigma = \frac{0.15\ \langle E \rangle}{2\sqrt{2\ln(2)}} \tag{S5}$$

We find that the fit results are weakly dependent on whether a Gaussian or a flux weighted Maxwell-Boltzmann distribution, as done in previous work [27,39,40], is used for describing the molecular beam profile. In all cases, the changes are smaller than the 1σ uncertainties of the fit parameters.

At high nozzle temperatures, the molecular beam also contains a significant fraction of H-atoms due to thermal dissociation. It is therefore crucial to include an atomic contribution to the measured sticking coefficient $\bar{S}(\langle E \rangle, T_n)$ according to equation (S6).

$$\bar{S}(\langle E \rangle, T_n) = \bar{S}_{\mathrm{mol}}(\langle E \rangle, T_n)\ (1 - x_{\mathrm{atom}}) + \frac{1}{2}\, S_{\mathrm{atom}}\ x_{\mathrm{atom}} \tag{S6}$$

The factor $1/2$ accounts for the fact that coverage was measured in terms of H_2 molecules. The sticking coefficient of H (or D) atoms, S_{atom}, is assumed to be unity, consistent with previous reports [41]. x_{atom} is the equilibrium molar fraction of atoms at a given T_{n} and pressure p_{n}, which we calculate using equation (S7). In the hot-nozzle p_{n} was 4 bar [42].

$$x_{\mathrm{atoms}} = -K(T_{\mathrm{n}},p_{\mathrm{n}})/2 + \sqrt{K^2(T_{\mathrm{n}},p_{\mathrm{n}})/4 + K(T_{\mathrm{n}},p_{\mathrm{n}})} \tag{S7}$$

The equilibrium constant $K(T_{\mathrm{n}},p_{\mathrm{n}})$ is defined by equation (S8) with the standard pressure p° and the free energy of dissociation $\Delta G(T_{\mathrm{n}})$. $\Delta G(T_{\mathrm{n}})$ is calculated from tabulated parameters for the Shomate equation [43]. We note that the Shomate parametrization agrees with statistical mechanic modelling based on molecular spectroscopic constants within 0.8% at all temperatures relevant to this work.

$$K(T_{\mathrm{n}},p_{\mathrm{n}}) = \frac{p^\circ}{p_{\mathrm{n}}} \exp\left(-\frac{\Delta G(T_{\mathrm{n}})}{k_{\mathrm{B}}T_{\mathrm{n}}}\right) \tag{S8}$$

The hot-nozzle data provides sticking probabilities for average *incidence* translational energy $\langle E_{\mathrm{i}} \rangle$ and incidence angle ϑ to the surface normal. With these values, each data point is converted to $\langle E \rangle = \langle E_{\mathrm{i}} \rangle \times \cos^n(\vartheta)$. The model (equation (S6)) is subsequently fitted to data points as a function of $\langle E \rangle$. Our fitting gave best results for $n = 1.6$ for both H_2 and D_2, whereas it is commonly assumed that $n = 2.0$, implying normal energy scaling. Our results are consistent with prior work on D_2 adsorption on Cu(111), which reported a violation of normal energy scaling ($n = 1.8$) [3,35,36].

There are some subtleties to using the hot nozzle data in this analysis. Each experimental data point should include a value for T_{n}. These were reported for most data points in the experimental studies, however not for all. There are some points—referred to as "pure H_2/D_2"—for which only the highest and the lowest nozzle temperatures were reported. Fortunately, the composition of the molecular beam associated with these points was not varied. Consequently, the nozzle temperature can be inferred from the beam's reported kinetic energy, as $\langle E \rangle \propto T_{\mathrm{n}}$. Another small problem in defining T_{n} arose at high temperature—we found that in the D_2 experiments, the data point with the highest reported nozzle temperature (2100 K) did not follow the $\langle E \rangle \propto T_{\mathrm{n}}$ expectation. This suggests an error associated with the measurement of T_{n}, which may arise at high temperature due to the increased temperature gradient between the thermocouple position and the actual nozzle. In light of this, we correct this very high nozzle temperature to be $T_{\mathrm{n}} = (2250 \pm 150)$ K, somewhat higher than the reported $T_{\mathrm{n}} = 2100$ K. For the sake of consistency, we apply this temperature also for the $T_{\mathrm{n}} = 2100$ K antiseeded data points. This allowed us to fit the data better, a fact that supports this approach to determining T_{n}. For the purposes of fitting, all data points are also assigned an uncertainty in the nozzle temperature ΔT_{n}, which increases with T_{n} to describe the greater influence of temperature gradients at high temperature. We therefore set $\Delta T_{\mathrm{n}} = 0$ K at 298 K and equate ΔT_{n} at the highest nozzle temperature to the difference between reported T_{n} and expected T_{n} when employing the linear $\langle E \rangle \propto T_{\mathrm{n}}$ scaling.

Optimized model parameters were determined (see Table S3) for H_2 using a global fitting routine considering all hot nozzle data. A separate global fit was performed for D_2. Figure S3 compares the model using these parameters to experimental data.

The fitting routine minimized the experimental-error-weighted root mean square deviation (ERMSD) between experimental ($\bar{S}_{\mathrm{exp},i}$) and modeled ($\bar{S}_{\mathrm{mod},i}$) sticking coefficients as defined in equation (S9) on a logarithmic scale. The fitting is performed on a logarithmic scale to balance the optimization to very low (10^{-6}) sticking probabilities and high sticking probabilities (10^{-1}).

$$\mathrm{ERMSD} = \sqrt{\frac{1}{N}\sum_{i}^{N}\left(\frac{\log_{10}\left(\bar{S}_{\mathrm{exp},i}\right) - \log_{10}\left(\bar{S}_{\mathrm{mod},i}\right)}{\sigma}\right)^2} \quad \text{(S9)}$$

In equation (S9), N is the number of all data points for a given isotopologue. σ is approximated as the difference between the modeled sticking coefficients using the upper and lower limit of the nozzle temperature uncertainty range according to equation (S10).

$$\sigma = \frac{1}{2}\left[\log_{10}\left(\bar{S}_{\mathrm{mod}}(T_{\mathrm{n}} + \Delta T_{\mathrm{n}})\right) - \log_{10}\left(\bar{S}_{\mathrm{mod}}(T_{\mathrm{n}} - \Delta T_{\mathrm{n}})\right)\right] \quad \text{(S10)}$$

This definition is chosen since the deviation between $\bar{S}_{\mathrm{mod}}(T_{\mathrm{n}} + \Delta T_{\mathrm{n}})$ and $\bar{S}_{\mathrm{mod}}(T_{\mathrm{n}} - \Delta T_{\mathrm{n}})$ is in most cases higher than the actual scatter of experimental data. Furthermore, this achieves a better representation of the low-temperature data points, which are crucial for the thermal sticking coefficient and puts less emphasis on the less important high-temperature data. The fitting procedure is performed in two steps: 1) optimization of the initial guess with the Nelder-Mead algorithm was implemented in the Python library SciPy and 2) another optimization with smaller step sizes and tolerances was performed using the result of the first step as the initial guess. For this fit, the Python library LMFIT and the Levenberg-Marquardt algorithm were used. This second optimization yields final fit parameters as well as their covariance matrices. We extract 1σ uncertainties for each fitting parameter from the square root of diagonal elements of the covariance matrices. Best-fit parameters are listed in Table S3. The set of quantum state resolved error function parameters for $T_{\mathrm{S}} = 120$ K is given in Table S4 for H_2 and in Table S5 for D_2.

It is important to note that the fit parameters δ_W for the excited states where an extrapolation was used to estimate W account not only for the surface temperature dependence of W, but also for possible inaccuracies of the extrapolation procedure. Therefore, δ_W of these states was fitted separately and it deviates from the other δ_W parameters for which experimental W parameters are available. The fact that the resulting parameter $W(v = 2) = 0.012$ eV (see Table S4) is lower than $W(v = 0)$ and $W(v = 1)$ is consistent with previous reports [2].

The experimentalists measured the two D_2 data points with highest incidence energy and highest nozzle temperature using H_2 as a seeding gas [36]. These points are shown in panel (b) of Figure S3 as circles with a black '+'. The King and Wells method instead of the TPD technique had to be used for determination of the sticking probability in these two experiments [36]. Since the sticking

coefficient is determined from the D_2 partial pressure rise with and without a crystal in the molecular beam [36], no correction for an atomic contribution to the sticking coefficient is required. We therefore evaluated our model with best fit parameters for these high kinetic energy data points excluding the contribution of D atoms to the sticking coefficient. Figure S3 (b) shows results of this evaluation as the two red crosses.

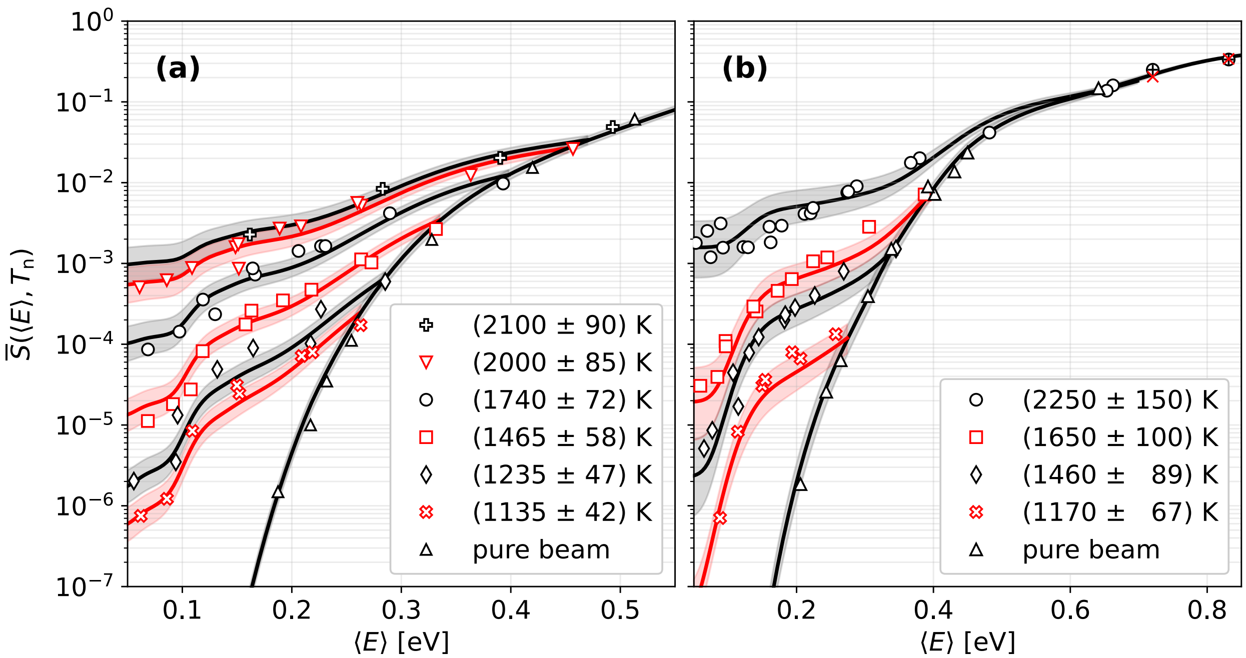

Figure S3: Model fit to absolute H_2 (panel (a)) and D_2 (panel (b)) hot nozzle adsorption measurements on Cu(111) at a surface temperature of 120 K. Data was reported by Rettner, Michelsen and Auerbach [2,35,36]. Experimental sticking coefficients (points) and optimized model results (solid lines) are plotted against $\langle E \rangle = \langle E_i \rangle \times \cos^{1.6}(\vartheta)$, where $\langle E_i \rangle$ is the mean incidence kinetic energy of the molecular beam profile and ϑ the angle with respect to the surface normal. For most of the curves, nozzle temperatures (as indicated in the legend) were held constant but beams were used with variable ratios of antiseeding gas. For curves indicated as "pure beam", nozzle temperatures range from 770 K to 2300 K for H_2 and from 875 K to 2100 K for D_2 [2,35,36]. Nozzle temperature uncertainties are assumed to decrease linearly with the nozzle temperature in both cases, their effect on the model result is indicated by the shaded region around the solid lines. The two data points marked with a black '+' have been measured using the King and Wells method and therefore do not include an atomic contribution to the sticking coefficient. The red crosses show an evaluation of the model ignoring the correction for atoms when computing the sticking coefficient.

Table S3: Resulting model parameters derived from the global optimization routine reproducing the absolute hot nozzle H_2 (D_2) adsorption measurements reported by Rettner, Michelsen and Auerbach [2,35,36]. The listed values either directly parametrize the sticking coefficients from equation (S1), or scale experimental values from Kaufmann et al.[34]. v and J are the vibrational and rotational quantum numbers, respectively. Listed uncertainties refer to 1 σ.

model parameter		this work	Literature
**H_2**			
$A(v)$	$A(0) = A(1) = A(2)$	0.43 ± 0.02	0.24[2], 0.33[34]
$\delta_W(v)$	$\delta_W(0) = \delta_W(1)$	0.56 ± 0.02	0.69[34]
	$\delta_W(2)$	0.09 ± 0.07	0.69[34]
$\delta_{E_0}(v)$	$\delta_{E_0}(2)$	2.4 ± 0.1	not fitted[34]
**D_2**			
$A(v)$	$A(0)$	0.36 ± 0.03	0.34[35], 0.51[34]
	$A(1)$	0.83 ± 0.09	0.34[35], 0.51[34]
	$A(2)$	0.17 ± 0.04	0.34[35], 0.51[34]
	$A(3)$	set to 1	0.34[35], not fitted[34]
$\delta_W(v)$	$\delta_W(0) = \delta_W(1) = \delta_W(2)$	0.50 ± 0.05	0.64[34]
	$\delta_W(3)$	0.25 ± 0.04	not fitted[34]
$E_0(J, v)$	$E_0(J, 3)$ [eV]	0.138 ± 0.006	0.115[35], not fitted[34]

Table S4: Parameters of the quantum state resolved sticking probability (equation (S1)) for H_2 at Cu(111) at a surface temperature $T_S = 120\ K$. $E_0(J, v = 0{,}1)$ was reported by Kaufmann et al.[34], remaining values result from fits to absolute adsorption data. J and v are the rotational and vibrational quantum numbers.

H_2	$v = 0$	$v = 1$	$v = 2$
$A(v)$	0.43 ± 0.02	0.43 ± 0.02	0.43 ± 0.02
$W(v)$ [eV]	0.106 ± 0.004	0.092 ± 0.004	0.012 ± 0.010
J		$E_0(J, v)$ [eV]	
0	0.708 ± 0.008	0.342 ± 0.005	0.09 ± 0.01
1	0.720 ± 0.008	0.350 ± 0.005	0.11 ± 0.01
2	0.721 ± 0.008	0.354 ± 0.005	0.12 ± 0.01
3	0.737 ± 0.008	0.364 ± 0.005	0.15 ± 0.01
4	0.723 ± 0.008	0.354 ± 0.006	0.13 ± 0.02
5	0.711 ± 0.008	0.344 ± 0.006	0.11 ± 0.02
6	0.671 ± 0.008	0.320 ± 0.007	0.06 ± 0.02
7	0.626 ± 0.008	0.281 ± 0.008	-0.03 ± 0.02
8	0.572 ± 0.008	$-$	$-$
9	0.509 ± 0.008	$-$	$-$
10	0.474 ± 0.008	$-$	$-$
11	0.375 ± 0.009	$-$	$-$

Table S5: Parameters of the quantum state resolved sticking probability (equation (S1)) for D_2 at Cu(111) at a surface temperature $T_S = 120\ K$. $E_0(J, v = 0,1,2)$ was reported by Kaufmann et al.[34], remaining values result from fits to absolute adsorption data. J and v are the rotational and vibrational quantum numbers.

D_2	$v = 0$	$v = 1$	$v = 2$	$v = 3$
$A(v)$	0.36 ± 0.03	0.83 ± 0.09	0.17 ± 0.04	1.0
$W(v)$ [eV]	0.100 ± 0.009	0.089 ± 0.008	0.080 ± 0.008	0.035 ± 0.006
J		$E_0(J, v)$ [eV]		
0	0.748 ± 0.006	0.477 ± 0.004	0.220 ± 0.005	0.138 ± 0.006
1	0.751 ± 0.006	0.484 ± 0.004	0.242 ± 0.005	0.138 ± 0.006
2	0.761 ± 0.006	0.492 ± 0.004	0.243 ± 0.005	0.138 ± 0.006
3	0.777 ± 0.006	0.497 ± 0.004	0.266 ± 0.005	0.138 ± 0.006
4	0.790 ± 0.006	0.497 ± 0.004	0.277 ± 0.006	0.138 ± 0.006
5	0.799 ± 0.006	0.494 ± 0.004	0.277 ± 0.009	0.138 ± 0.006
6	0.797 ± 0.006	0.488 ± 0.004	0.286 ± 0.008	0.138 ± 0.006
7	0.781 ± 0.006	0.462 ± 0.004	0.257 ± 0.008	0.138 ± 0.006
8	0.762 ± 0.006	0.449 ± 0.004	0.248 ± 0.006	0.138 ± 0.006
9	0.729 ± 0.006	0.401 ± 0.004	−	−
10	0.691 ± 0.006	−	−	−
11	0.639 ± 0.006	−	−	−
12	0.600 ± 0.007	−	−	−
13	0.538 ± 0.006	−	−	−
14	0.501 ± 0.007	−	−	−

We use the root mean square deviation (RMSD) to quantify the quality of our fit. We also calculated the RMSD for previous fitting attempts of this type, which allows us to compare the fit quality on an absolute scale. To enhance sensitivity for low sticking coefficients, we calculate the RMSD on a logarithmic scale. Results are shown in Table S6.

$$\mathrm{RMSD} = \sqrt{\frac{1}{N}\sum_{i}^{N}\left(\log_{10}\left(\bar{S}_{\mathrm{exp},i}\right) - \log_{10}\left(\bar{S}_{\mathrm{mod},i}\right)\right)^2} \quad \text{(S11)}$$

Table S6: Root-mean-square deviations (RMSD) according to equation (S11) between experimental sticking coefficients and model results on a logarithmic scale in comparison to literature results. For H_2, several models in the literature were not evaluated for the entire range of nozzle temperatures. We therefore compute RMSDs for three subsets of sticking data to allow for a quantitative comparison. The column "all curves" refers to the full set of experimental data available. For any choice of the experimental data subset our fit outperforms previous work.

	reference	all curves	without "pure beam"	without 1235 K, 2100 K
H_2	**this work**	**0.137**	**0.137**	**0.125**
	Rettner *et al.*[2]			0.132
	Kaufmann *et al.*[34]	0.234	0.227	0.227
	Abbott and Harrison[5]		0.451	
D_2	**this work**	**0.159**	**0.168**	
	Kaufmann *et al.*[34]	0.257	0.223	
	Michelsen *et al.*[37]	0.277	0.215	
	Nattino *et al.*[40]	0.297	0.211	
	Abbott and Harrison[5]		0.486	

S2.2. Quantum-State Resolved Kinetic Energy Distributions

In the analysis of the last section, the absolute adsorption data measured at $T_S = 120$ K used input values—$E_0(v, J)$ and $W(v, T_S)$—from experiments performed at $T_S = 923$ K of Kaufmann *et al.* [34]. This required introducing a T_S dependence to W, where only two temperatures were considered. We turn now to the determination of $W(v,T_S)$ over a broader range of temperatures. This is possible using data from Murphy and Hodgson who, similar to Kaufmann *et al.*, measured state-selected translational energy distributions for recombinatively desorbing H_2 and D_2 upon exposure with atomic hydrogen, but at $T_S = 370, 600$ & 900 K[44]. Since the desorption flux $F_{des}(E, J, v, T_S)$ is directly related to the sticking probability via equation (S12), the desired surface temperature dependence can be obtained.

$$F_{des}(E, J, v, T_S) \propto S(E, J, v, T_S) \times E \exp\left(-\frac{E}{k_B T}\right) \mathrm{d}E \qquad \text{(S12)}$$

The $E_0(J, v)$ parameters from Kaufmann's work (at $T_S = 923$ K) were found to describe absolute adsorption data at $T_S = 120$ K, indicating that $E_0(J, v)$ can be treated as temperature independent, consistent with previous reports [2,34,35]. Therefore, W is the only parameter in the sticking probability model that exhibits a dependence on surface temperature. We obtain excellent agreement with Murphy and Hodgson's energy distributions using equation (S12) and $W(v, T_S)$ as the only fitting parameter. We emphasize that here, the $E_0(J, v)$-parameters are fixed to those obtained by Kaufmann *et al.* This avoids correlations with the fitted $W(v, T_S)$ and ensures consistency of all parameters of equation (S1).

Fit results are plotted against the surface temperature in Figure S4 as black squares. Since the energy distributions are very sensitive to $W(v, T_S)$, uncertainties for the results are negligibly small and therefore not shown. We also show $W(v,T_S = 120$ K) derived from fits to absolute adsorption data as open circles. They fall precisely on the expected temperature dependent curve predicted from analysis of the Hodgson data (filled squares). There is a large discrepancy at highest surface temperatures between results derived from Murphy and Hodgson's data and Kaufmann's values (open squares in Figure S4). This was reported previously [34]. We cannot clarify this inconsistency in the data set. We speculate that the calibration of ion collection efficiency results in an underestimation of the width of the obtained energy distributions in the work of Kaufmann *et al.* [34]. The overlap of a low kinetic energy channel, observed concurrently with the hyperthermal channel [34], which is of interest for this work, could also cause uncertainties in Kaufmann's fits of the width parameter. In all the following, we consider only $W(v, T_S)$ derived from Murphy and Hodgson's experiments, as they consistent with the hot nozzle adsorption data and provide information important to the low temperature range, which is crucially important to the derivation of the thermal adsorption rate constants.

Of course, evaluation of the thermal adsorption rate constant requires a continuous surface temperature dependence of $W(v, T_S)$. We therefore employ a fit to all available $W(v, T_S)$ at $T_S \leq$ 900 K using a second-degree polynomial (solid line in Figure S4). An uncertainty range for this

polynomial was estimated from the deviation of the fitted $W(v, T_S)$ and the $T_S = 120$ K results from the previous section. The parameters for these polynomials are given in Table S7.

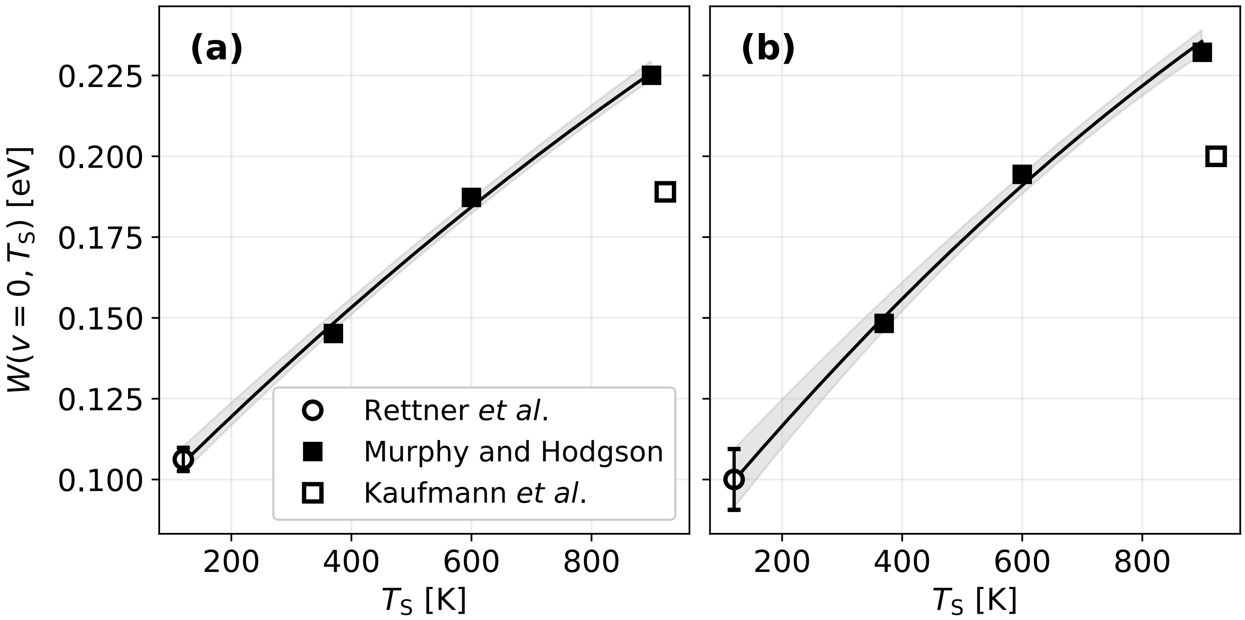

Figure S4: W-parameters determined from fits to absolute adsorption data (circles), reported by Rettner et al.[2,35,36]*, and state-selected kinetic energy distributions of the products of recombinative desorption studied by Murphy and Hodgson* [44] *(black squares) and Kaufmann et al.*[34] *(open squares). Panel (a) shows results for* H_2 *and panel (b) for* D_2*. We find that the W-parameter exhibits a surface temperature* (T_S) *dependence and derive a continuous relationship between W and* T_S *by fitting a second-degree polynomial equation to the obtained data points (solid black line) and indicate an uncertainty range (gray shaded regions around solid lines). The polynomial parameters are given in Table S7. These polynomials were used without further adjustment in the fitting of the absolute sticking probabilities shown in Fig. S2 and in the main text. The observed surface temperature dependence is inconsistent with measurements of W from Kaufmann et al.*[34]*, see text for a discussion.*

Table S7: Second-degree polynomial parameters which were found to describe the surface temperature dependence of the W-parameter (see solid line in Figure S4). We estimate a 1σ uncertainty for these parameters by employing additional fits that limit the range of experimentally tolerated W-parameters (shown as gray regions around solid lines in Figure S4).

$W(v=0, T_S)/\text{eV} = a\left(\frac{T_S}{K}\right)^2 + b\left(\frac{T_S}{K}\right) + c$	limit	H_2	D_2
	lower	-3.76	-7.44
$a \times 10^8$	**best**	**-3.40**	**-5.30**
	upper	-2.20	-3.60
	lower	1.94	2.56
$b \times 10^4$	**best**	**1.89**	**2.28**
	upper	1.75	2.03
	lower	7.94	6.17
$c \times 10^2$	**best**	**8.29**	**7.30**
	upper	8.95	8.56

Using this polynomial surface temperature dependence, we calculate expected translational energy distributions for desorbing H_2 and D_2 based on equation (S12), which we then compared to experimental distributions from Murphy and Hodgson (see Figure S5). The agreement is excellent, confirming the validity of treatment of T_S dependence just described. Previous work has reported parameters that fail to describe these observations (see colored lines in Figure S5).

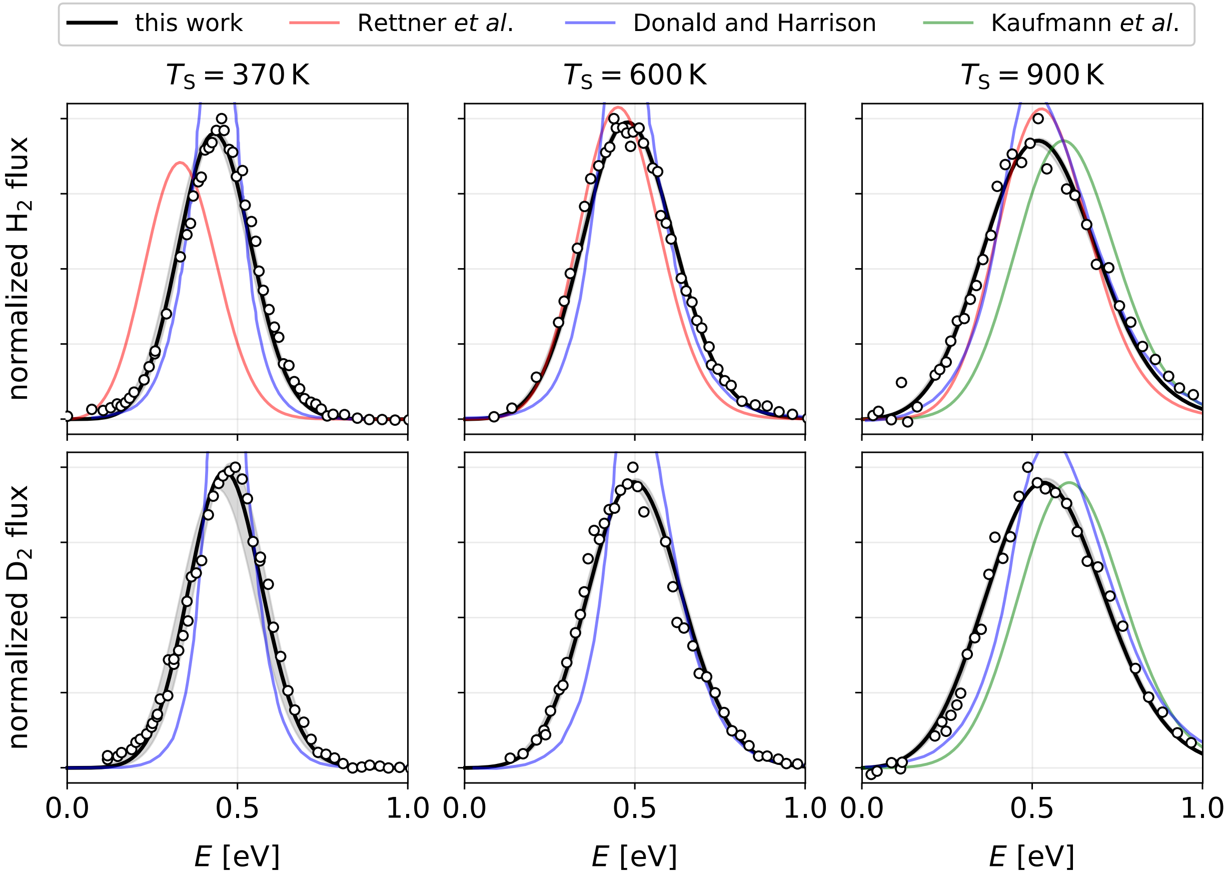

Figure S5: State-selected kinetic energy distributions of desorbing H_2 ($v = 0, J = 1$) and D_2 ($v = 0, J = 2$) (open circles) at the indicated surface temperatures T_S which were reported by Murphy and Hodgson [44]. Solid black lines are based on (S12) with Kaufmann's E_0 - parameters and a temperature dependent W-parameter according to Table S7. We also show that previous models proposed by Rettner et al.[2,35], Donald and Harrison [4], and Kaufmann et al.[34] are inconsistent with the observed temperature dependence.

As shown in Figure S5, we successfully derived the temperature dependence $W(v, T_S)$ from Murphy and Hodgsen's results on H_2 $(v = 0, J = 1)$ and $D_2(v = 0, J = 2)$ [44]. As above, we assume that $W(v, T_S)$ is independent of J [34,35,44]. Murphy and Hodgson conducted their experiments for $v = 0$ only. We obtained temperature dependent W-parameters for vibrationally excited states of H_2 and D_2 using equation (S13).

$$W(v > 0, T_S) = W(v = 0, T_S) \times \frac{W(v > 0, T_S = 120\text{ K})}{W(v = 0, T_S = 120\text{ K})} \qquad \text{(S13)}$$

This procedure was motivated from fits to the absolute adsorption data, where the fit parameters that scale the vibrational ground and excited state W-parameters are the same in our model $\delta_W(v = 0) = \delta_W(v = 1)$ (see Table S3).

S2.3. Angular Density Distributions of Desorbing H_2 and D_2

Angular distributions of desorbing molecules provide a sensitive measure of the angular dependence of the sticking probability. Given that we identified a deviation from normal energy scaling in subsection S2.1, we can verify our results against independently measured angular distributions. These distributions are also available over a wide range of surface temperatures, providing another test of the T_S dependent model we established in subsection S2.2.

To accomplish this, we rely on the principle of detailed balance to write an expression—equation (S14)—for the *flux*-weighted desorption probability $\langle D\rangle(J,v,T,\vartheta)$ as a function of the angle ϑ with respect to the surface normal. Note that we omit the index of the surface temperature T_S since under thermal equilibrium considerations gas-phase and surface temperature are equal.

$$\langle D\rangle(J,v,T,\vartheta) = \cos^{n/2}(\vartheta)\frac{\int_{E=0}^{\infty} S(E,J,v,T)\, E\exp\left(-\frac{E/\cos^n(\vartheta)}{k_\mathrm{B}T}\right)\mathrm{d}E}{\int_{E=0}^{\infty} E\exp\left(-\frac{E/\cos^n(\vartheta)}{k_\mathrm{B}T}\right)\mathrm{d}E} \tag{S14}$$

Rettner *et al.* measured the angular *density* distribution of desorbing H_2 and D_2 from Cu(111) at various surface temperatures [45]. To compare the present model with their data, results of equation (S14) are first divided by the mean thermal velocity $\langle v\rangle(J,v,T,\vartheta)$ of desorbing molecules (equation (S15)) to yield a *density* distribution [35].

$$\langle v\rangle(J,v,T,\vartheta) = \frac{\int_{E=0}^{\infty}\sqrt{\frac{2E}{m\cos^n(\vartheta)}}\, S(E,J,v,T)\, E\exp\left(-\frac{E/\cos^n(\vartheta)}{k_\mathrm{B}T}\right)\mathrm{d}E}{\int_{E=0}^{\infty} S(E,J,v,T)\, E\exp\left(-\frac{E/\cos^n(\vartheta)}{k_\mathrm{B}T}\right)\mathrm{d}E} \tag{S15}$$

Equations (S14) and (S15) are evaluated numerically with E ranging from 0 to 4 eV within 1200 evenly spaced steps, which leads to well converged results. Since experimental angular distributions were not resolved by quantum state, modeled density distributions are weighted with the respective Boltzmann population distribution $P_\mathrm{Boltzm.}(J,v,T)$ and summed to yield the quantum state averaged density distribution $\Phi(T,\vartheta)$. This summation is done over all rotational states listed in Table S4 and Table S5 and up to $v = 1$ for H_2 and up to $v = 2$ for D_2, inclusive. We assume that the ionization and detection efficiency in the mass spectrometer is independent of the rovibrational quantum state. We ensured convergence in Boltzmann populations for all experimental conditions. Consistent with our finding from fits to absolute adsorption data, we use $n = 1.6$ in all cases.

$$\Phi(T,\vartheta) = \sum_{J,v} P_\mathrm{Boltzm.}(J,v,T)\times\frac{\langle D\rangle(J,v,T,\vartheta)}{\langle v\rangle(J,v,T,\vartheta)} \tag{S16}$$

Results are shown in Figure S6 without additional optimization. The ability of our temperature dependent sticking model to describe these desorption angular distributions is a significant improvement over past work [4].

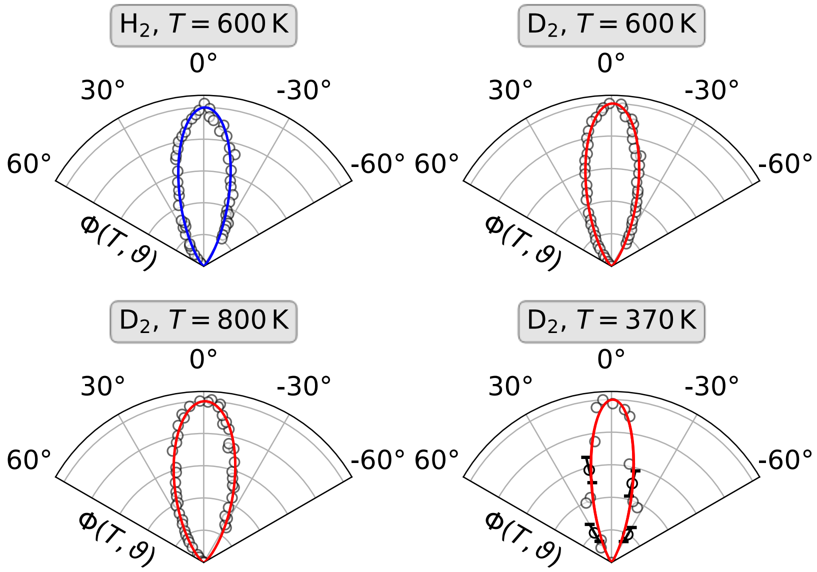

Figure S6: Angular density distributions $\Phi(T,\vartheta)$ of desorbing H_2 and D_2 from Cu(111) as a function of the polar angle ϑ for different surface temperatures T. Open circles: experimental data from Rettner et al.[45], solid lines: model results without additional optimization of previously derived parameters.

S2.4. Additional Figures on Sticking Probability Measurements

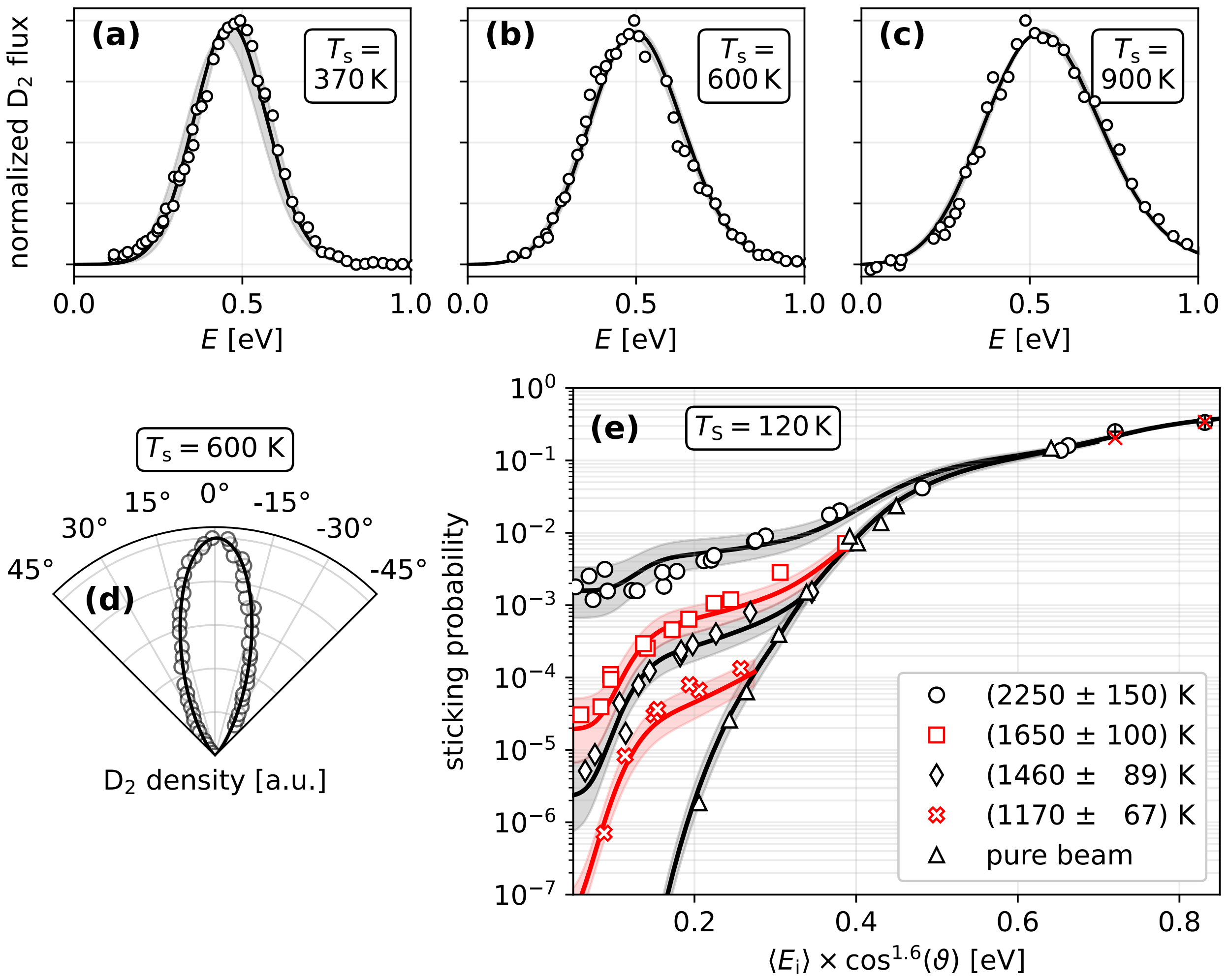

Figure S7: Experimental recombinative desorption dynamics and dissociative sticking probabilities for D_2 compared to the fitted model. (a)-(c) Experimentally determined state resolved translational energy (E) distributions of D_2 (v=0, J=2) desorbing from Cu(111) at various surface temperatures T_S (open circles)[44]*. The solid line shows the fit achieved with the sticking model of this work. (d) Experimental angular density distribution of desorbing D_2 from Cu(111) at T_S=600 K (open circles) from ref. 45 and fit achieved with the model of this work (solid line). (e) Hot nozzle data for D_2 sticking on Cu(111) at T_S=120 K. Experimental data (symbols) from ref. 35,37 is compared to the fit (solid lines) achieved in this work. The uncertainty of the fit emerges from the uncertainty of the nozzle temperature, indicated by the grey shaded region. Data for (d) and (e) are not quantum state resolved, therefore model results were obtained from the Boltzmann average over the quantum states—see SI section S2.1 for details of the analysis. The two data points marked with a black '+' have been measured using the King and Wells method and therefore do not include an atomic contribution to the sticking coefficient. The red crosses show an evaluation of the model ignoring the correction for atoms when computing the sticking coefficient.*

S3. Thermal Sticking Coefficient and Adsorption Rate Constant

We used the dynamically derived sticking probabilities $S(E, \vartheta, J, v, T)$ obtained in section S2 to calculate the thermally averaged sticking coefficient $\langle S \rangle(T)$ for temperatures between 200 K and 1000 K using equations (S17) and (S18). Note that we now use T in place of T_S since at thermal equilibrium, the gas-phase and surface temperature are identical.

$$\langle S \rangle(T) = \sum_{J,v} P_{\text{Boltzm.}}(J, v, T) \times \langle S \rangle(J, v, T), \text{where} \tag{S17}$$

$$\langle S \rangle(J, v, T) = \frac{\int_{E=0}^{\infty} \int_{\vartheta=0}^{\pi/2} S(E, \vartheta, J, v, T) \frac{E}{\cos^{2n}(\vartheta)} \exp\left(-\frac{E/\cos^{n}(\vartheta)}{k_B T}\right) \sin(\vartheta) \cos^{n/2}(\vartheta)\, d\vartheta dE}{\int_{E=0}^{\infty} \int_{\vartheta=0}^{\pi/2} \frac{E}{\cos^{2n}(\vartheta)} \exp\left(-\frac{E/\cos^{n}(\vartheta)}{k_B T}\right) \sin(\vartheta) \cos^{n/2}(\vartheta)\, d\vartheta dE} \tag{S18}$$

Convergence of the integrals in (S18) was obtained with numerical integration over the energy E from 0 up to 4 eV using 1200 evenly spaced steps and over ϑ from 0 up to $\pi/2$ using 300 evenly spaced steps. Further increase of the upper limit of E or the number of steps in the integration did not influence the result to within 0.1% at all temperatures. The summation in equation (S17) was performed up to $v = 1(2)$ for $H_2(D_2)$ inclusive and over all rotational states listed in Table S4 and Table S5. These states account for $1 - 10^{-5}(1 - 10^{-4})$ of the total Boltzmann population at the highest evaluated temperature for $H_2(D_2)$. Uncertainties for $\langle S \rangle(T)$ were determined by evaluating equations (S17) and (S18) using the lower and upper limits of the 1σ fit parameter uncertainties from Table S3 as well as the uncertainty for the derived temperature dependence of $W(v, T_S)$.

Thermal sticking probabilities and thermal adsorption rate constants $k_{\text{ads}}(T)$ are closely related—equation (S19)—by the thermal average velocity $\langle v_z^+ \rangle$ along one (positive) direction of space.

$$k_{\text{ads}}(T) = \langle S \rangle(T) \times \langle v_z^+ \rangle \quad \text{with:} \quad \langle v_z^+ \rangle = \sqrt{\frac{k_B T}{2\pi m}} \tag{S19}$$

Here, m is the mass of the H_2/D_2 molecule. Note that k_{ads} has units of $\text{m} \cdot \text{s}^{-1}$, such that upon multiplication by the reactant's gas-phase particle density, units of adsorption flux are obtained.

Figure S8 shows Arrhenius plots of $k_{\text{ads}}(T)$ obtained in this way for H_2 and D_2. The one-way velocity exhibits negligible curvature in a similar Arrhenius plot. Hence, many of the points we are about to make for $k_{\text{ads}}(T)$ also apply to $\langle S \rangle(T)$. In Figure S8 we also show $k_{\text{ads}}(T)$ obtained from prior work. All studies are in reasonable agreement at high temperature but diverge dramatically with decreasing temperature. We attribute this to the fact that previous studies aimed toward an accurate description of recombination experiments, mainly performed at $T_S \approx 920$ K, and did not focus on an accurate description of absolute adsorption data, performed at $T_S = 120$ K.

Figure S5 shows that these previous attempts also fail to reproduce energy distributions of recombinative desorption at reduced temperatures.

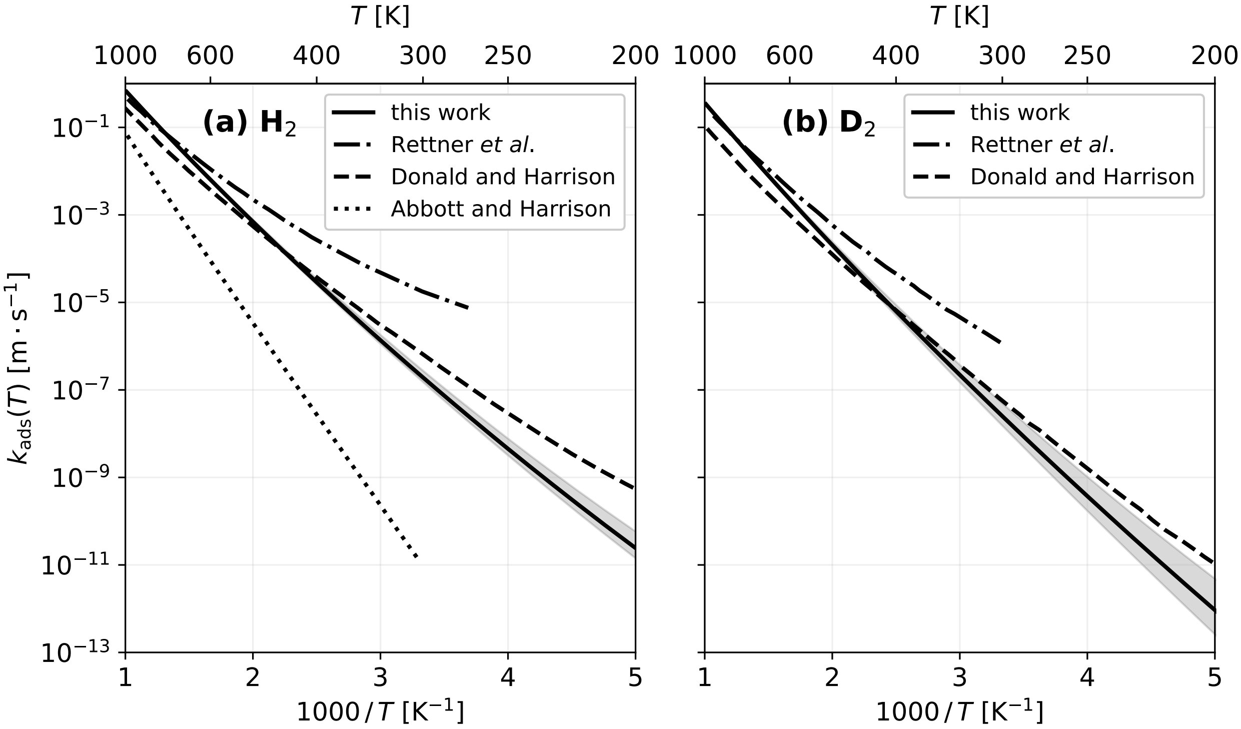

Figure S8: Thermal rate constants for thermal dissociative adsorption of hydrogen—panel (a)—and deuterium—panel (b)—on Cu(111). Results from this work are shown as solid lines. Transparent regions around solid lines indicate uncertainties. Literature data was taken from Rettner et al.[2,3], Donald and Harrison [4] and Abbott and Harrison [5]. In cases where thermal sticking coefficients were reported instead of adsorption rate constants, we employed equation (S19).

We also calculated the adsorption rate constant for specific vibrational quantum states individually to understand their contribution to the thermal adsorption rate. To do this we excluded the summation over v in equation (S17) but still applied equations (S18) and (S19). Results of this analysis—see Figure S9—show that the thermal adsorption rate constant for temperatures $\leq$1000 K is dominated by the contribution of molecules in $v = 0$. As expected, the contribution of excited vibrational quantum states increases with increasing temperature and is larger for D_2 when compared to H_2 at a given temperature. As a result, the D_2 adsorption rate constant at 1000 K has a considerable contribution of 34% of molecules in excited vibrational states.

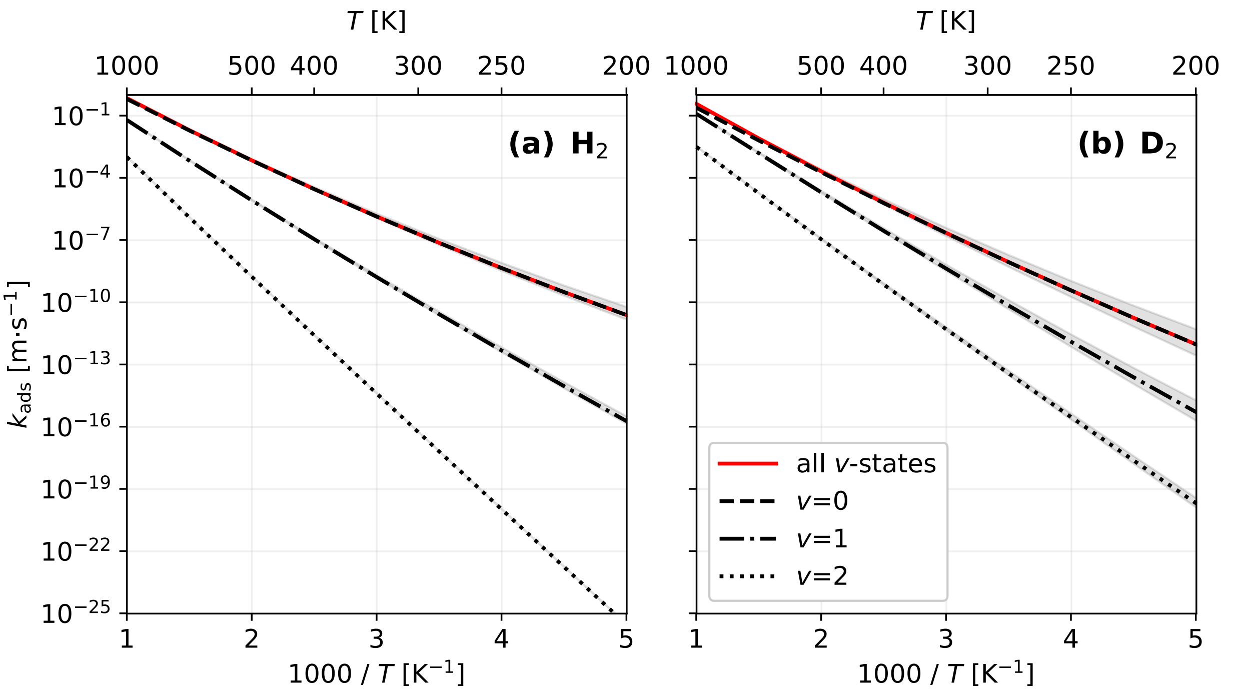

Figure S9: Contribution of vibrational quantum states v to the thermal adsorption rate constant k_{ads}. Results for H_2 are shown in panel (a) and for D_2 in panel (b). The red solid line represents a Boltzmann-weighted average rate constant over the vibrational quantum states v=0, 1 and 2. The dashed, dash-dotted and dotted black lines show Boltzmann-weighted the contributions of the individual quantum states v=0, 1 and 2, respectively, i.e. the sum of the black lines is equal to the red line. The transparent regions around the lines indicate uncertainties.

S4. Adsorption Rate Constant Modeling using Transition State Theory

In this subsection, we construct a transition state theory (TST) model for H_2/D_2 adsorption on Cu(111). We perform global fitting of this model to thermal rate constants, which we have derived from previous reaction dynamics experiments on this system (see section S2 & S3). The outcome of this fit allows a determination of the barrier height for dissociative adsorption and a test of the influence of tunneling on the adsorption process. We begin by describing the limitations encountered with transition state theory, and then proceed to introduce corrections to overcome these limitations. Finally, the fitting procedure and fit results used to make the corrections are presented.

Constructing the hydrogen adsorption rate constant within harmonic transition state theory (hTST)—equation (S20)—requires knowledge of the zero-point energy (ZPE) corrected dissociative barrier height $E^{\ddagger}_{\mathrm{ads,H_2}}$, as well as the partition functions for the transition state ($Q^{\mathrm{H_2}}_{\mathrm{TS}}$) and the gas-phase molecule ($Q^{\mathrm{H_2}}_{\mathrm{gas}}$). Note that one must normalize both the gas-phase as well as the transition state partition function to the unit volume V and unit cell area A, respectively to obtain the correct units for k_{ads} ($\mathrm{m \cdot s^{-1}}$).

$$k^{\mathrm{H_2}}_{\mathrm{hTST}} = \frac{k_{\mathrm{B}}T}{h}\frac{Q^{\mathrm{H_2}}_{\mathrm{TS}}/A}{Q^{\mathrm{H_2}}_{\mathrm{gas}}/V}\exp\left(-\frac{E^{\ddagger}_{\mathrm{ads,H_2}}}{k_{\mathrm{B}}T}\right) \tag{S20}$$

$Q^{\mathrm{H_2}}_{\mathrm{gas}}$ is based on spectroscopic constants for diatomic molecules from the NIST database [46]. Transition state (TS) partition functions $Q^{\mathrm{H_2}}_{\mathrm{TS}}$ can be evaluated within the harmonic oscillator approximation for nonreactive TS modes ν_i as shown in equation (S21).

$$Q^{\mathrm{H_2}}_{\mathrm{TS}} = \prod_{i=1}^{i=5}\left[1 - \exp\left(-\frac{h\nu_i}{k_{\mathrm{B}}T}\right)\right]^{-1} \tag{S21}$$

We calculated transition state frequencies with DFT using several exchange-correlation functionals, see Table S13. The variation of $Q^{\mathrm{H_2}}_{\mathrm{TS}}$ induced by different choices of functional is minor—as shown for $T = 300$ K in Table S13—an observation that gives us confidence to use DFT derived frequencies for our TST model. In our estimation of uncertainties, we include this small effect. All given transition state frequencies and barrier heights refer to hydrogen dissociation at Cu(111) bridge sites, yielding hydrogen atoms in the adjacent hollow sites, as this pathway has a minimum energy barrier. This is consistent with reference 27. Within the unit cell of the Cu(111) surface there are three equivalent bridge sites for H_2 dissociation, each with a two-fold rotational symmetry. Thus, when normalizing (S20) to the unit cell area A, a six-fold TS degeneracy $d_{\mathrm{TS}} = 6$ needs to be introduced. For A we used 5.66 $\mathrm{Å^2}$, which is derived from the experimentally known lattice constant [47]. In equation (S20), we do not include corrections that arise from electronic partition functions due to the fact that for H_2 adsorption on Cu(111) the TS resembles the reactant state and therefore corrections are likely to cancel between $Q^{\mathrm{H_2}}_{\mathrm{TS}}$ and $Q^{\mathrm{H_2}}_{\mathrm{gas}}$.

The harmonic approximation for nonreactive TS modes may introduce error, especially at high temperatures. Therefore, we include anharmonicity correction factors a^{H_2} and a^{D_2}. To minimize the number of fit parameters, we approximate both factors as being temperature independent.

An important limitation of equation (S20), and for that matter TST in general, arises from the assumption that all trajectories crossing the dividing plane at the transition state reach the product state without returning to the reactant state. This non-recrossing assumption introduces errors which we address by introducing a re-crossing correction factor $\kappa \leq 1$ to equation (S20). H_2/D_2-Cu(111) re-crossing coefficients were previously calculated by explicitly counting the number of ab-initio molecular dynamics trajectories that recrossed the TS before reaching the product state [48]. The results do not depend strongly on the specific method [48] used to calculate the recrossing. The recrossing correction is, in principle, temperature dependent; hence, we linearly interpolated between available values at 300 K and 924 K to obtain $\kappa(T)$. Below 300 K we use a linear extrapolation based the available values.

Transition state theory *per se* relies on classical mechanics and thus neglects quantum effects, such as tunneling. This problem has been addressed in the past by introducing multiplicative tunneling correction factors Γ [49,50]. Tunneling corrections, in principle, require knowledge of the complete shape of the potential energy surface near the barrier; but, good methods have been developed using an approximate one-dimensional potential energy function near the barrier relying on separability of the reaction coordinate and nonreactive modes, analogous to TST[50]. Here, the potential energy along the reaction coordinate is described by an analytic function—*e.g.* an Eckart potential [51-53]—for which exact quantum mechanical transmission probabilities are known [50,51]. We employed the a*symmetrical* Eckart potential for the tunneling corrections. This function is characterized by three parameters: The barrier heights $E^{\ddagger}_{\text{ads}}$ and $E^{\ddagger}_{\text{des}}$ from either side of the barrier and the imaginary frequency at the TS, which determines the curvature of the one-dimensional potential barrier [51-53]. We explicitly use zero-point corrected barrier heights instead of their classical analogues, which was discussed and suggested in reference 50. We employ a numerical calculation of $\Gamma(T, E^{\ddagger}_{\text{ads}}, E^{\ddagger}_{\text{des}}, \nu^{\ddagger})$ following reference 53.

To summarize the discussion above, we have introduced four additional factors to equation (S20): the six-fold transition state degeneracy for each Cu(111) surface atom and corrections for recrossing, anharmonicity and tunneling. The fully elaborated TST model is shown in equation (S22) with X = H or D.

$$k^{X_2}_{\text{TST}}(T) = \kappa^{X_2}(T)\, a^{X_2}\, \Gamma^{X_2}\left(T, E^{\ddagger}_{\text{ads},X_2}, E^{\ddagger}_{\text{des},X_2}, \nu^{\ddagger}_{X_2}\right) \frac{k_B T}{h} \frac{d_{\text{TS}}\, Q^{X_2}_{\text{TS}}/A}{Q^{X_2}_{\text{gas}}/V} \exp\left(-\frac{E^{\ddagger}_{\text{ads},X_2}}{k_B T}\right) \quad \text{(S22)}$$

Note that both H_2 and D_2 share the same classical barrier height for adsorption $\varepsilon^{\ddagger}_{\text{ads}}$ and the respective ZPE's are simply related, as shown in equation (S23).

$$E^{\ddagger}_{\text{ads},X_2} = \varepsilon^{\ddagger}_{\text{ads}} + \text{ZPE}_{X_2^{\ddagger}} - \text{ZPE}_{X_2} \quad \text{with:} \quad X = \text{H, D} \quad \text{(S23)}$$

ZPE_{H_2} and ZPE_{D_2} are the molecular zero-point energy of gas-phase H_2 and D_2, respectively—these were determined from spectroscopic data. Likewise, the imaginary frequencies of the two isotopologues are related simply to one another, within the Eckart tunneling model $\nu^{\ddagger}_{D_2} = \nu^{\ddagger}_{H_2}/\sqrt{2}$. We show the relationships in Figure S10 as a sketch of the potential energy path along the reaction coordinate.

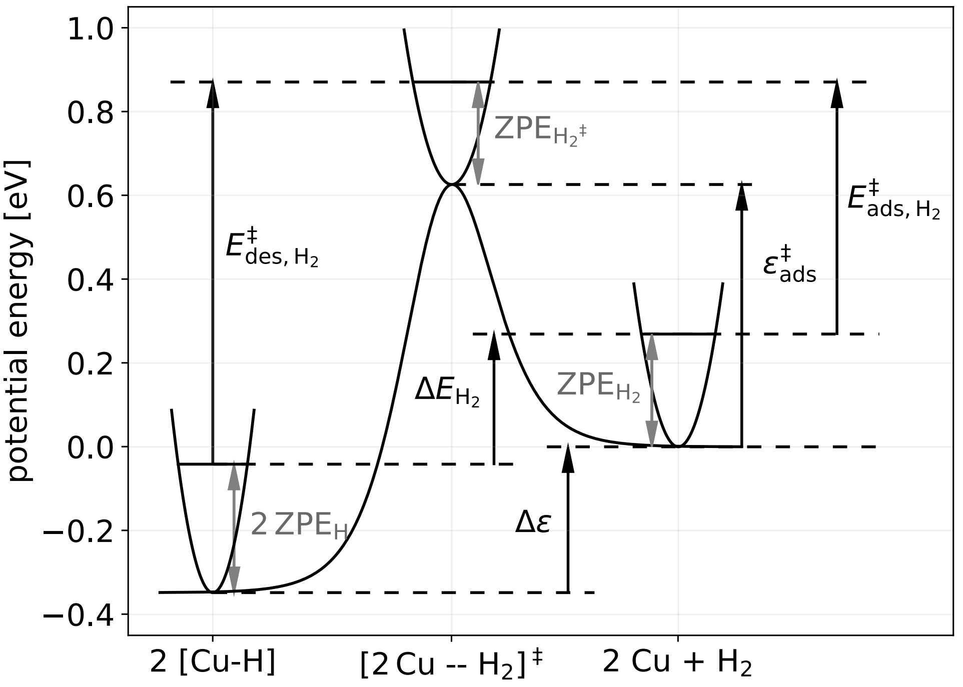

Figure S10: Schematic representation of the potential energy path along the reaction coordinate for hydrogen dissociation on Cu(111).

The TST model can thus be applied to both H_2 and D_2 adsorption with only four fit parameters: the classical barrier height for adsorption, an anharmonicity correction factor for each isotopologue and one imaginary TS frequency. We determine best fit values in a global optimization procedure to the rate constants of both isotopologues, minimizing the error-weighted root mean square deviation ERMSD between experimentally derived (k^{exp}_{ads}) and modeled (k_{TST}) rate constants on a logarithmic scale. N is the number of all data, σ is the uncertainty of experimentally derived rate constants and estimated as follows.

$$\text{ERMSD} = \sqrt{\frac{1}{N}\sum_{i}^{N}\left(\frac{\log_{10}\left(k^{exp}_{ads,i}\right) - \log_{10}\left(k_{TST,i}\right)}{\sigma}\right)^{2}} \qquad (S24)$$

$$\sigma = \frac{1}{2}\left[\log_{10}(k^{exp}_{ads,i} + \Delta k^{exp}_{ads,i}) - \log_{10}(k^{exp}_{ads,i} - \Delta k^{exp}_{ads,i})\right] \qquad (S25)$$

Table S8 shows the best-fit parameters for six choices of DFT exchange-correlation functional. The optimized fit parameters do not depend significantly on the choice of the exchange-correlation functional. We present in the last row, a weighted average across the six functionals of each optimized parameter—here, the weighting is determined by the quality of each fit. When comparing fitted classical barrier height to DFT values from Table S13 we find best agreement to the SRP functionals. Of course, this agreement is not fortuitous as the SRP functional was tuned to better reproduce experimental sticking probabilities [27].

Table S8: Optimized model parameters for fitting equation (S22) to experimentally derived H_2 and D_2 adsorption rate constants in a global optimization routine. The model has four adjustable parameters: the classical barrier height for adsorption $\varepsilon^{\ddagger}_{ads}$, the imaginary transition state frequency $\tilde{\nu}^{\ddagger}_{H_2}$ and anharmonicity corrections a^{H_2/D_2}. These results also provide zero-point energy corrected barrier heights $E^{\ddagger}_{ads}$. Results found using six functionals are shown together with a weighted average.

	$\varepsilon^{\ddagger}_{\mathrm{ads}}$ [eV]	$E^{\ddagger}_{\mathrm{ads,H_2}}$ [eV]	$E^{\ddagger}_{\mathrm{ads,D_2}}$ [eV]	$\tilde{\nu}^{\ddagger}_{\mathrm{H_2}}$ [cm^{-1}]	$a^{\mathrm{H_2}}$	$a^{\mathrm{D_2}}$
PBE-TS	$0.606^{+0.017}_{-0.024}$	$0.592^{+0.017}_{-0.024}$	$0.595^{+0.017}_{-0.024}$	1043^{+74}_{-15}	$1.8^{+0.3}_{-0.5}$	$1.9^{+0.2}_{-0.3}$
PBE	$0.610^{+0.018}_{-0.023}$	$0.595^{+0.018}_{-0.023}$	$0.598^{+0.018}_{-0.023}$	1037^{+83}_{-11}	$2.4^{+0.5}_{-0.6}$	$2.6^{+0.3}_{-0.4}$
optB86b	$0.620^{+0.020}_{-0.020}$	$0.592^{+0.020}_{-0.020}$	$0.600^{+0.020}_{-0.020}$	1010^{+109}_{-10}	$2.7^{+0.6}_{-0.5}$	$3.1^{+0.5}_{-0.3}$
SRP48	$0.615^{+0.019}_{-0.021}$	$0.597^{+0.019}_{-0.021}$	$0.601^{+0.019}_{-0.021}$	1029^{+96}_{-8}	$3.0^{+0.7}_{-0.6}$	$3.4^{+0.5}_{-0.4}$
RPBE	$0.622^{+0.019}_{-0.019}$	$0.596^{+0.019}_{-0.019}$	$0.603^{+0.019}_{-0.019}$	1014^{+108}_{-13}	$3.5^{+0.8}_{-0.7}$	$4.0^{+0.6}_{-0.4}$
vdW-DF2	$0.640^{+0.010}_{-0.023}$	$0.580^{+0.010}_{-0.023}$	$0.596^{+0.010}_{-0.023}$	924^{+55}_{-91}	$2.4^{+0.2}_{-0.5}$	$3.0^{+0.0}_{-0.4}$
weighted average	$0.619^{+0.018}_{-0.021}$	$0.593^{+0.016}_{-0.023}$	$0.599^{+0.017}_{-0.023}$	1013^{+80}_{-33}	$2.7^{+0.4}_{-0.7}$	$3.0^{+0.2}_{-0.5}$

Figure S11 shows the results of the fitting of equation (S22) to the experimentally derived adsorption rate constants as dashed red (H_2) and blue (D_2) lines. Here, the dashed lines were obtained with the model relying on SRP48 transition state frequencies. The agreement is excellent at all temperatures. Specifically, the model captures the observed curvature—much more pronounced for H_2 compared to D_2—in the Arrhenius plots. This supports conclusions in this work concerning the importance of tunneling. Neglecting the tunneling corrections yields the dotted red and blue lines in Figure S11—this leads to dramatically smaller rate constants especially at $T <$ 400 K.

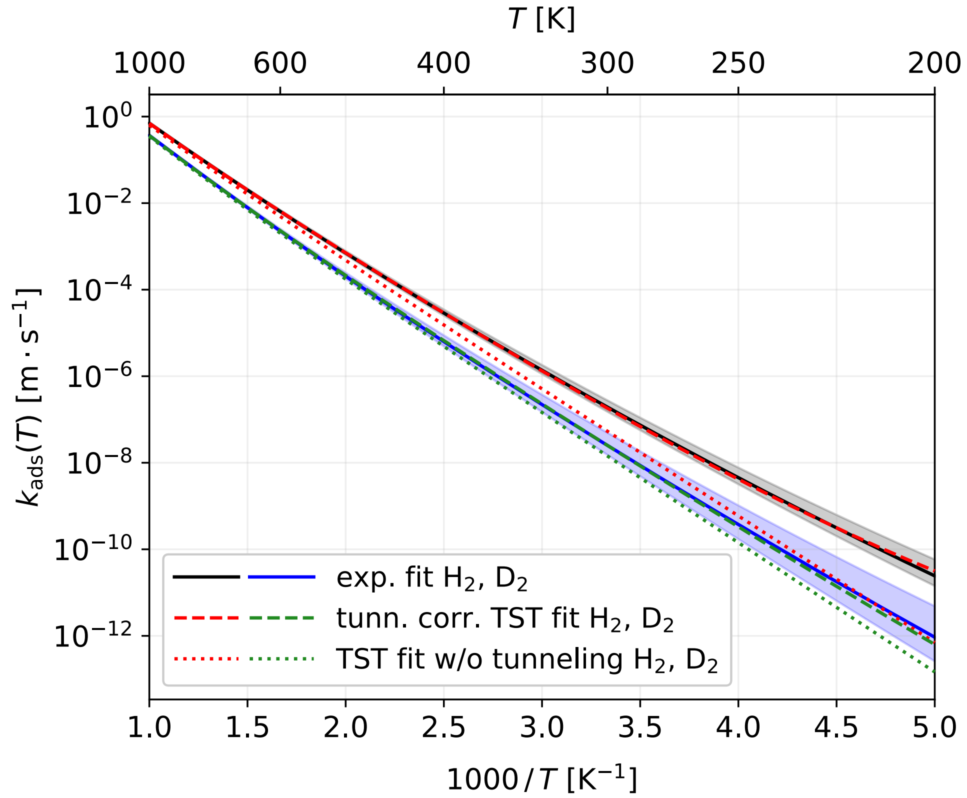

Figure S11: The modified transition state theory expression from equation (S22) was fitted in a global optimization routine to experimentally derived H_2 and D_2 thermal adsorption rate constants (shown as solid lines). The result of this fit is shown as dashed lines (here, using SRP48 transition state frequencies). We highlight the importance of tunneling in the dissociative adsorption process by showing the fitted model neglecting Eckart tunneling corrections, as the dotted lines.

We would like to point out that the uncertainty intervals of the weighted averages in Table S8 reflect statistical errors from fits to k_{ads}^{exp} and deviations from input parameters using different DFT functionals, but they ignore any systematic errors that arise from the level of approximations used to evaluate the rate expression. Such systematic errors can arise from the use of recrossing coefficients computed from ab-initio molecular dynamics under classical treatment of the equations of motion. Another limitation in the current model is the assumption that anharmonic contributions to the transition state partition function are temperature independent or the approximation of the true tunneling correction with results for an analytic function along the minimum energy path (MEP). For example, it is known that tunneling often proceeds through paths other than the MEP and that accurate tunneling corrections need to consider the exact tunneling path and the potential along it [54,55].

S5. Adsorption and Desorption Rate Constants from Ring Polymer Molecular Dynamics

S5.1. Six-Dimensional Potential Energy Surface

A neural network (NN) PES was constructed for H_2 interacting with a rigid Cu(111) surface from DFT data obtained using the Vienna *Ab-initio* Simulation Package (VASP) [56,57]. In these calculations, the Kohn-Sham valence electronic wave function was expanded in a plane-wave basis set with a kinetic energy cutoff at 400 eV, while the ionic core-electron interactions were described by the projector-augmented wave (PAW) method [58]. The surface of Cu(111) was modelled by a four-layer rigid slab with a vacuum region of 15 Å in the vertical direction within a (2 × 2) surface supercell (effective H* coverage of 0.5 ML). The Brillouin zone was sampled using a 9 × 9 × 1 Γ–centered k-points grid mesh. As discussed in the main paper, we use the PBEα-vdW functional with $\alpha = 0.57$ [30,59,60] as a semi-empirical exchange-correlation functional.

This PES for H_2 interacting with Cu(111) is represented by a permutation invariant polynomial neural network (PIP-NN) [61,62], with a total of 1508 DFT points using a trajectory-free active learning strategy [63,64]. A total root mean squared error (RMSE) of 7.85 meV was obtained. The minimum energy path for H_2 dissociation on Cu(111) goes through the highest point at the bridge site, associated with a barrier of 0.607 eV. The adsorption energy is 0.227 eV with two hydrogen atoms located in adjacent fcc hollow sites. A contour plot for the PES is shown in Figure S12, where the center of mass (COM) of H_2 is fixed on the bridge site and the angular coordinates are optimized.

To match the experimental energetics in the limit of zero hydrogen atom coverage, we added the following empirical correction potential V_{adj} to the original PES,

$$V_{\mathrm{adj}}(Z,r) = \frac{a_1}{2}\left(1 + \tanh\left(2\frac{Z}{\text{Å}} - 7\right)\right) + \frac{a_2}{2}\left(1 + \tanh\left(5\frac{r}{\text{Å}} - 9\right)\right), \quad \text{(S26)}$$

where $a_1 = -0.012$ eV, $a_2 = -0.133$ eV, Z is the Z component of the COM of H_2 and r is the distance between the two hydrogen atoms. This scaling changes neither the PES topography nor the vibrational frequencies of the stationary points to any significant degree. In total, V_{adj} increases the adsorption energy by 121 meV (from 0.227 to 0.348 eV) and increases the adsorption barrier by 12 meV (from 0.607 to 0.619 eV). The modification of the adsorption energy equals the sum of two corrections: (1) the experimental coverage dependence (104 meV) to effectively calculate RPMD rate constants in the limit of zero hydrogen atom coverage; (2) a correction for small differences between DFT and experimental energetics (17 meV). The modification of the adsorption barrier is only due to differences between DFT and experimental energies. The geometries (r and Z) at the transition state and in the adsorbed state are shown in Table S9.

We emphasize that we explicitly chose more efficient computational settings to calculate the 1508 energy values to which the neural network PES was fitted. This is reasonable, since the neural network PES is slightly modified by the above-mentioned shifts to match the energetics extracted from experiments.

Table S9: Distance between the two hydrogen atoms (r) and Z component of the center of mass of the H_2 molecule above the surface (Z) at the transition state and in the adsorbed state.

	r [Å]	Z [Å]
Transition State	1.06	1.15
Adsorbed State	2.57	0.89

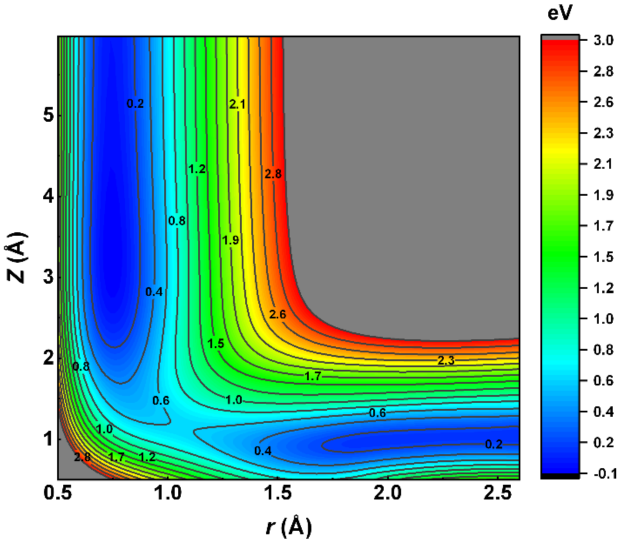

Figure S12: Two-dimensional contours of the uncorrected PES (without addition of V_{adj}) with the COM of H_2 fixed on the bridge site of Cu(111) and the angular coordinates optimized.

S5.2. Ring Polymer Molecular Dynamics Rate Theory

The RPMD rate theory for gas-phase bimolecular reactions has been extensively discussed in the literature [65,66]. In such calculations, each atom is approximated by a ring polymer consisting of a number of harmonically connected beads that follow classical mechanics. Quantum statistical properties are obtained by taking advantage of the isomorphism with the fictitious classical ring polymer [67]. Importantly, the RPMD theory is amenable to large systems as the computational cost scales linearly with the number of atoms.

The extension of RPMD rate theory to dissociative chemisorption (DC) was presented in our recent work [68]. To this end, two dividing surfaces are introduced in terms of the ring polymer centroid variables $(\bar{\boldsymbol{q}})$. Specifically, the first dividing surface

$$s_0^{\mathrm{DC}}(\bar{\boldsymbol{q}}) = Z_\infty - Z = 0\,, \qquad \text{(S27)}$$

is located in the reactant asymptote where the molecule is far from the surface. Here, Z_∞ is an adjustable parameter that is chosen to make the interaction between the molecule and the surface negligible. The second dividing surface

$$s_1^{\mathrm{DC}}(\bar{\boldsymbol{q}}) = (Z^{\ddagger} - Z) - (r^{\ddagger} - r) \tag{S28}$$

is located at the barrier. Here $Z^{\ddagger}$ and $r^{\ddagger}$ correspond to values at the saddle point of the PES.

We further extend the RPMD rate theory to recombinative desorption (RD) of adsorbed hydrogen atoms. To this end, we assume that the adsorbed atoms are two-dimensional free gas, and the first dividing surface

$$s_0^{\mathrm{RD}}(\bar{\boldsymbol{q}}) = (r_{\mathrm{ads}} - r) = 0 \tag{S29}$$

is located in the reactant region where two hydrogen atoms are adsorbed at two adjacent fcc sites at 0.5 ML coverage. Here r_{ads} is the corresponding distance at the adsorbed state of the PES. The second dividing surface is defined by equation (S30).

$$s_1^{\mathrm{RD}}(\bar{\boldsymbol{q}}) = -s_1^{\mathrm{DC}}(\bar{\boldsymbol{q}}) = 0 \tag{S30}$$

As given in reference 69, a suitable interpolating reaction coordinate $\xi(\bar{\boldsymbol{q}})$ can be constructed connecting the two dividing surfaces as follows,

$$\xi(\bar{\boldsymbol{q}}) = \frac{s_0(\bar{\boldsymbol{q}})}{s_0(\bar{\boldsymbol{q}}) - s_1(\bar{\boldsymbol{q}})}, \tag{S31}$$

such that $\xi \to 0$ as $s_0 \to 0$ and $\xi \to 1$ as $s_1 \to 0$.

In practice, the RPMD rate constant $k^{\mathrm{RPMD}}(T)$ is recast [69,70] in the Bennett–Chandler factorization form [71,72],

$$k^{\mathrm{RPMD}}(T) = k^{\mathrm{QTST}}(T;\xi^{\ddagger})\,\kappa(t \to t_p;\xi^{\ddagger})\,. \tag{S32}$$

Here, $\kappa(t \to t_p;\xi^{\ddagger})$, is the long-time limit of the time-dependent ring polymer transmission coefficient. It is a dynamical correction to account for recrossing of the dividing surface $\xi(\bar{\boldsymbol{q}}) = \xi^{\ddagger}$ and is given by equation (S33).

$$\kappa(t \to t_p;\xi^{\ddagger}) = \lim_{t \to t_p} \frac{\langle f_\xi(\bar{\boldsymbol{q}})^{-1} v_\xi(\boldsymbol{p},\boldsymbol{q}) h[\xi(\bar{\boldsymbol{q}}_t) - \xi^{\ddagger}] \rangle_{\xi^{\ddagger}}}{\langle f_\xi(\bar{\boldsymbol{q}})^{-1} v_\xi(\boldsymbol{p},\boldsymbol{q}) h[v_\xi(\bar{\mathbf{p}},\bar{\boldsymbol{q}})] \rangle_{\xi^{\ddagger}}}, \tag{S33}$$

where the subscripts of the brackets indicate that the averages are over the constrained ensemble at $\xi(\bar{\boldsymbol{q}}) = \xi^{\ddagger}$ and the factor $f_\xi(\bar{\boldsymbol{q}})^{-1}$ is a metric tensor correction for the effect of the constraint [65,69,73]. $k^{\mathrm{QTST}}(T;\xi^{\ddagger})$, is the centroid-density quantum transition-state theory (QTST) rate constant [74] evaluated at the position of the reaction coordinate ($\xi^{\ddagger}$) corresponding to maximum in the potential of mean force (PMF). For DC [68],

$$k^{\mathrm{QTST}}(T;\xi^{\ddagger}) = \left(\frac{1}{2\pi\beta M}\right)^{\frac{1}{2}} e^{-\beta[W(\xi^{\ddagger}) - W(0)]}, \tag{S34}$$

with $\beta = (k_B T)^{-1}$. For RD, on the other hand,

$$k^{\mathrm{QTST}}(T;\xi^{\ddagger}) = 2\pi r_{\mathrm{ads}}\left(\frac{1}{2\pi\beta\mu_r}\right)^{\frac{1}{2}} e^{-\beta[W(\xi^{\ddagger})-W(0)]}\ . \tag{S35}$$

Here, $M = 2m_{\mathrm{H}}$ and $\mu_r = m_{\mathrm{H}}/2$, where m_{H} is the mass of the hydrogen atom. $W(\xi)$ is the PMF calculated by using umbrella integration [66,75,76],

$$W(\xi^{\ddagger}) - W(0) = \int_0^{\xi^{\ddagger}} \frac{\sum_{i=1}^{N_{\mathrm{windows}}} N_i P_i(\xi)\left(\frac{1}{\beta}\frac{\xi-\bar{\xi}_i}{(\sigma_i)^2} - k_i(\xi-\xi_i)\right)}{\sum_{j=1}^{N_{\mathrm{windows}}} N_j P_j(\xi)} d\xi\ , \tag{S36}$$

with the probability distribution

$$P_i(\xi) = \frac{1}{\sigma_i\sqrt{2\pi}} \exp\left[-\frac{1}{2}\left(\frac{\xi-\bar{\xi}_i}{\sigma_i}\right)^2\right], \tag{S37}$$

N_{windows} is the number of biasing windows placed along the reaction coordinate, each with a specific value ξ_i assigned to each window. N_i is the total number of steps sampled for window i. $\bar{\xi}_i$ and σ_i^2 are the mean value and the variance calculated for the ith window from the umbrella trajectory, respectively. Note that the corresponding rate constant for DC is in $\mathrm{m} \cdot \mathrm{s}^{-1}$ and for RD in $\mathrm{m}^2 \cdot \mathrm{s}^{-1}$, according to equations (S34) and (S35), respectively. The derivation of equation (S35) can be found in section S5.3.

In this work, the surface is assumed to be rigid, which is a reasonable approximation for this system [77]. For the calculation of the PMF using an umbrella sampling technique, the range of reaction coordinate (–0.05 to 1.10 for DC and –0.05 to 1.55 for RD) was divided into bins of equal size ($d\xi = 0.01$, centered at ξ_i) with the force constant $k = 2.72$ eV (T/K) of the harmonic biasing potential, where T is the temperature.

$$V_{\mathrm{bias}}(\xi) = \frac{1}{2}k(\xi - \xi_i)^2 \tag{S38}$$

This bias potential is introduced in each window so that the system is sampled near the center of the window. This potential is later removed when the free energy profile is spliced together.

In each sampling window, 60 trajectories of 100 ps were calculated, each initiated after 20 ps equilibration with the Andersen thermostat [78]. Once the PMF is determined at each temperature, the transmission coefficient was computed. Specifically, a long (2 ns) parent trajectory was carried out with the ring-polymer centroid constrained at the peak of PMF via the SHAKE algorithm [79], after an initial equilibration period of 20 ps. Configurations were saved once every 2 ps. For each of these configurations, 100 separate unconstrained ring polymer trajectories were spawned with different initial momenta sampled from the Boltzmann distribution. These trajectories were then propagated for 150 fs, which is long enough for the transmission coefficients to reach plateau

values. The time step is selected to be 0.1 fs in all RPMD calculations. The classical rate constants were also calculated for comparison by setting the number of beads to one.

For DC, the thermal rate constants were calculated at several temperatures ranging from 200 to 1000 K. The parameter Z_∞ in equation (S27) was set to 7 Å for all of the temperatures considered and the dividing surface $s_1(\bar{\boldsymbol{q}}) = 0$ was placed at the saddle point associated with the bridge site ($\xi = 1.0$ or $Z^\ddagger = 1.15$ Å and $r^\ddagger = 1.06$ Å). The converged RPMD PMFs for H_2/D_2 DC along the reaction coordinate ξ are displayed in Figure S13(a) and Figure S13(d), respectively. In Figure S13(b) and Figure S13(c), the classical and RPMD PMFs of H_2 DC are compared at two representative temperatures (300 and 700 K), which show that the RPMD free-energy barrier is significantly lower than the classical counterpart. This trend becomes more pronounced at lower temperatures. Likewise, we can see from Figure S13(e) and Figure S13(f) that the RPMD free-energy barrier of D_2 DC is also significantly lower than the classical counterpart, although the difference is smaller than that of H_2 DC. As a result, the centroid-density QTST rate constant is significantly larger than the classical TST counterpart, owing primarily to the contribution from quantum mechanical tunneling.

The converged RPMD transmission coefficients $\kappa(t;\xi^\ddagger)$ for H_2/D_2 DC, are shown in Figure S14(a) and Figure S14(b), respectively. All the transmission coefficients reach plateau values after an initial drop from one. The smaller quantum transmission coefficient for H_2 than D_2, which has been observed in many previous studies, is presumably due to the centroid expression of the dividing surface [65].

The final RPMD rate constants for H_2/D_2 DC are listed in Table S10, and are compared to the experimentally derived rate constants in Figure S15. We find excellent agreement over the entire temperature range. In particular, the non-Arrhenius curvature at low temperatures is accurately reproduced, underscoring the importance of quantum mechanical tunneling [68]. The figure also clearly shows that the rate constants from converged RPMD simulations are larger at all temperatures than those from classical limit, which exhibit a nearly perfect Arrhenius behavior (linear proportionality between $\ln k$ and $1/T$). Figure S15 also shows that the difference in the rate constants between the classical and RPMD simulations increases with decreasing temperature, reaching a factor of 10^4 at 200 K. This difference can be attributed to signifcant quantum effects.

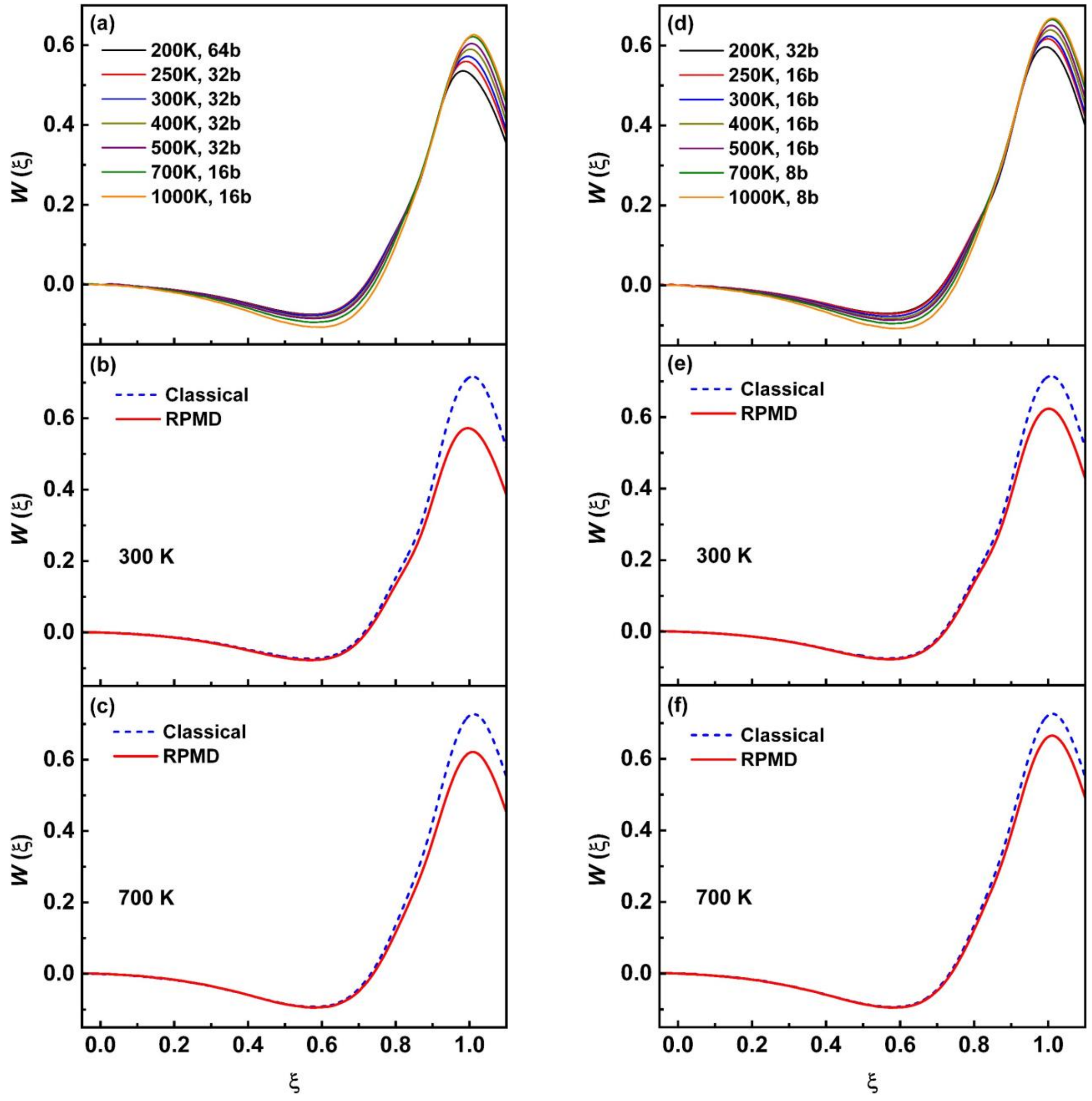

Figure S13: Converged RPMD PMFs $W(\xi)$ in eV for the H_2 (left column) and D_2 (right column) DC on Cu(111) – the number of beads (N_{bead}) are shown in the legend. The classical (dashed blue line) and RPMD (solid red line) PMFs are compared at two temperatures (b, c and e, f).

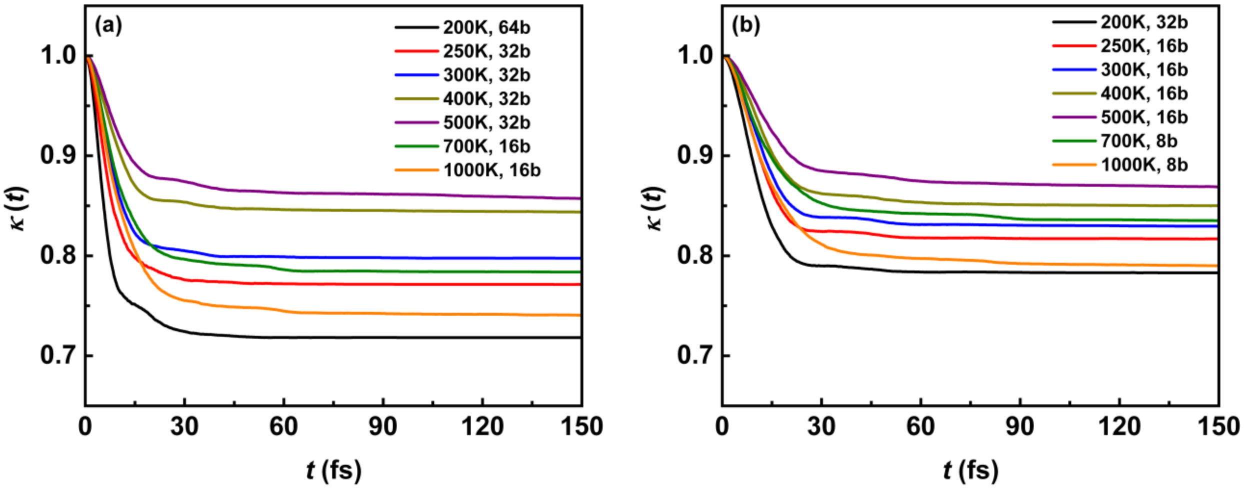

Figure S14: Converged RPMD transmission coefficients $\kappa(T)$ for (a) H_2 and (b) D_2 dissociative chemisorption on Cu(111) (with the number of beads N_{bead} shown in the legend).

Table S10: Summary of centroid-density QTST rate constants, k^{QTST}, transmission coefficients, κ, and converged RPMD rate constants, k^{RPMD}, for dissociative chemisorption of H_2/D_2 on Cu(111) at temperatures T between 200 and 1000 K.

	T [K]	N_{bead}	k^{QTST} $[\mathrm{m}\cdot\mathrm{s}^{-1}]$	κ	k^{RPMD} $[\mathrm{m}\cdot\mathrm{s}^{-1}]$
	200	64	2.48E-11	0.718	1.78E-11
	250	32	4.57E-09	0.771	3.52E-09
	300	32	2.21E-07	0.798	1.76E-07
H_2	400	32	3.90E-05	0.844	3.29E-05
	500	32	9.56E-04	0.857	8.19E-04
	700	16	4.69E-02	0.784	3.68E-02
	1000	16	1.15E+00	0.741	8.52E-01
	200	32	4.87E-13	0.783	3.81E-13
	250	16	2.14E-10	0.817	1.75E-10
	300	16	2.19E-08	0.830	1.82E-08
D_2	400	16	6.55E-06	0.850	5.57E-06
	500	16	2.32E-04	0.869	2.02E-04
	700	8	1.58E-02	0.835	1.32E-02
	1000	8	4.96E-01	0.790	3.92E-01

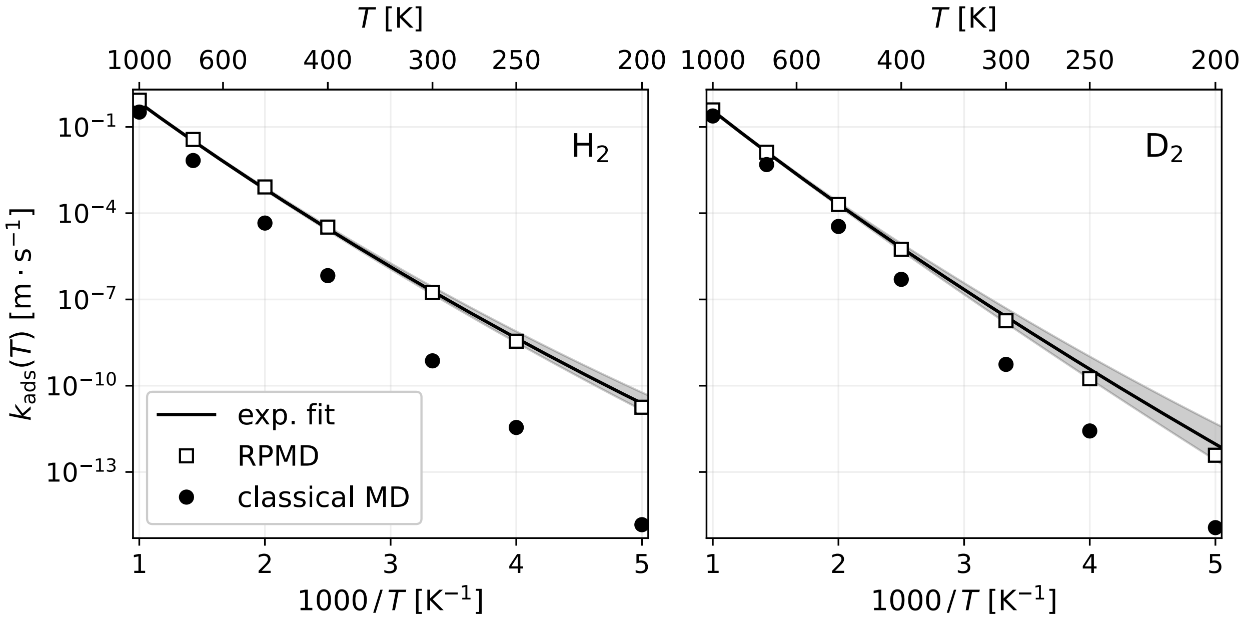

Figure S15: Rate constants for dissociative adsorption of H_2 (left panel) and D_2 (right panel) on Cu(111) calculated with the RPMD rate theory (shown as squares) and classical MD (one bead RPMD, shown as circles). Experimentally derived rate constants are shown as solid lines with uncertainties indicated by the grey region around the solid lines.

The thermal rate constants for recombinative desorption were also calculated at a number of temperatures ranging from 200 to 1000 K. The parameter r_{ads} in equation (S29) was set to 2.57 Å for all temperatures considered and the $s_1(\bar{\boldsymbol{q}}) = 0$ dividing surface was placed at the same saddle point at the bridge site. The converged RPMD PMFs of H_2/D_2 RD along the reaction coordinate ξ are shown in Figure S16(a) and (d). In Figure S16(b) and Figure S16(c), the classical and RPMD PMFs of H_2 RD are compared at two representative temperatures (325 and 700 K). Clearly, the RPMD free-energy barrier is lower than the classical counterpart due to ZPE and tunneling effects, and the quantum-classical difference in the barrier height becomes smaller at higher temperatures. Interestingly, the difference between the quantum and classical free-energy barriers is much larger for DC than RD at similar temperatures. Likewise, in Figure S16(e) and Figure S16(f), the RPMD free-energy barrier of D_2 RD is also lower than that of the classical counterpart, and this isotope effect can also be attributed to a contribution of tunneling.

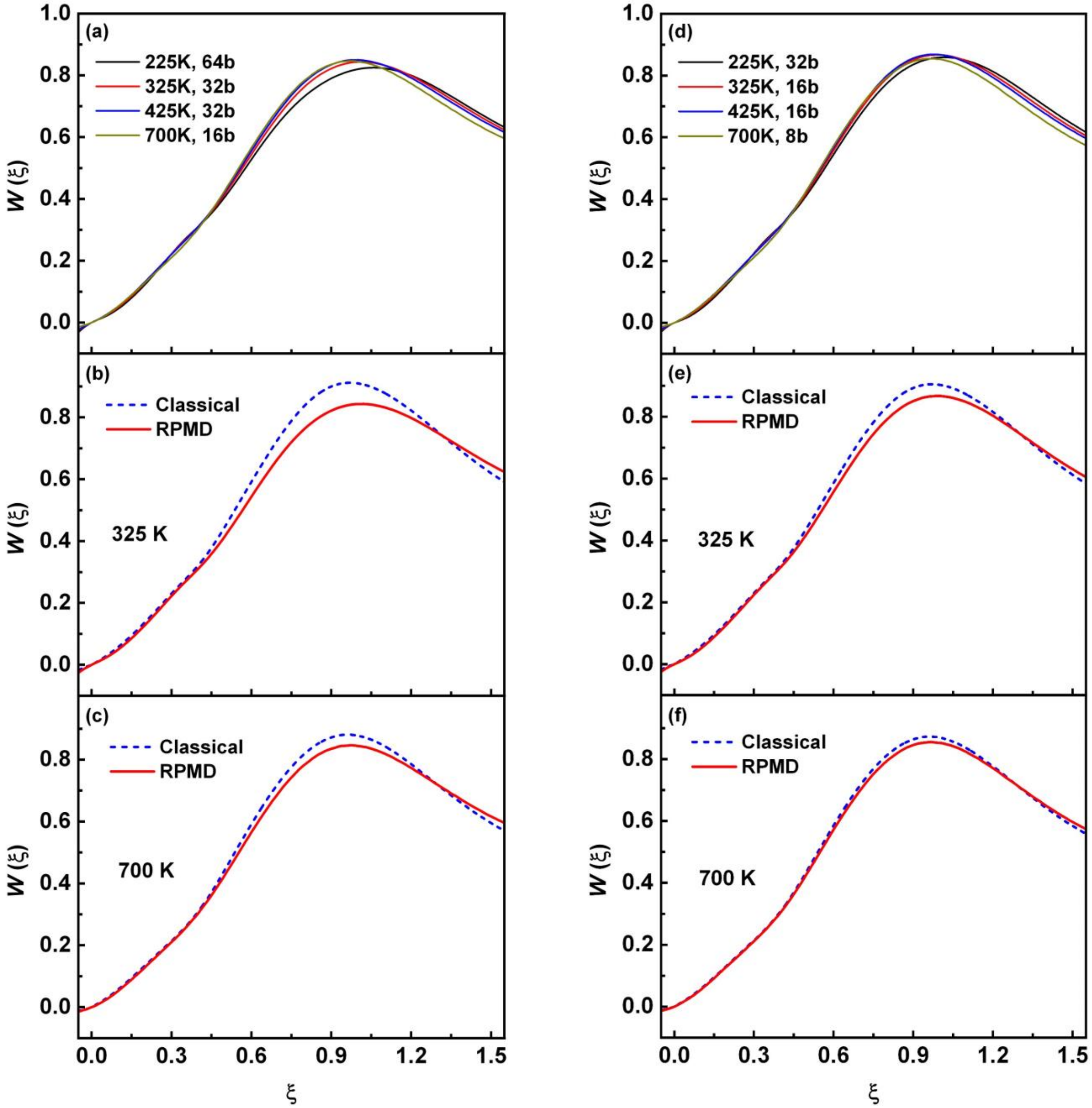

Figure S16: Converged RPMD PMFs $W(\xi)$ in eV for H_2 (left column) and D_2 (right column) RD on Cu(111) – the number of beads (N_{bead}) are shown in the legend. The classical (dashed blue line) and RPMD (solid red line) PMFs are compared at two temperatures (b, c and e, f).

The converged RPMD transmission coefficients of H_2/D_2 RD, $\kappa(t;\xi^{\ddagger})$, are shown in Figure S17(a) and Figure S17(b), respectively. Similar to the behavior seen for DC, all the transmission coefficients converge after initial drops from one and the transmission coefficients of H_2 RD are slightly lower than that of D_2.

The results of equation (S32) for the desorption process are listed in Table S11. To get the rate constants for recombinative desorption, two corrections need to be included: First, for systems with two indistinguishable particles, one has to symmetrize the wave function with respect to the exchange symmetry in a quantum mechanical treatment [80]. However, the classical mechanics based RPMD method does not account for this exchange symmetry, and therefore, a symmetry number of 2 needs to be introduced [81]. Moreover, as shown in reference 82, only one out of four electron spin combinations of two H atoms yields a ground electronic state gas-phase H_2 molecule. In total, the RPMD recombinative desorption rate constants shown in Figure S18 have been divided by a factor of 8 to account for the symmetry and the electronic spin effect.

The RPMD rate constants are in very good agreement with the experimentally derived rate constants over the entire temperature range. The converged RPMD rate constants are larger than those from the classical limit at all temperatures.

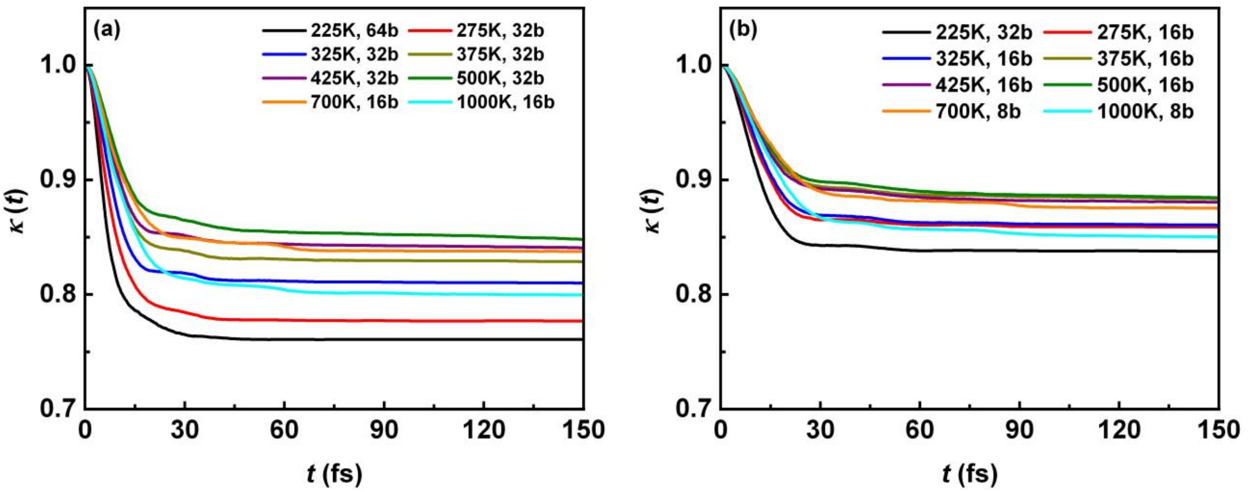

Figure S17: Converged RPMD transmission coefficients $\kappa(T)$ for (a) H_2 and (b) D_2 recombinative desorption from Cu(111) (with the number of beads N_{bead} shown in the legend).

Table S11: Summary of centroid-density QTST rate constants, k^{QTST}, transmission coefficients, κ, and converged RPMD rate constants, k^{RPMD}, for H_2/D_2 recombinative desorption on Cu(111) at temperatures T between 225 and 1000 K. Note that no correction factors for the indistinguishability of the two atoms in the recombination reaction or the electronic spin effect have been applied to these values.

	T [K]	N_{bead}	k^{QTST} $[\mathrm{m}^2 \cdot \mathrm{s}^{-1}]$	κ	k^{RPMD} $[\mathrm{m}^2 \cdot \mathrm{s}^{-1}]$
H_2	225	64	4.19E-25	0.761	3.19E-25
	275	32	6.58E-22	0.777	5.11E-22
	325	32	1.25E-19	0.810	1.01E-19
	375	32	6.47E-18	0.829	5.36E-18
	425	32	1.43E-16	0.841	1.20E-16
	500	32	4.79E-15	0.848	4.06E-15
	700	16	1.77E-12	0.837	1.48E-12
	1000	16	1.55E-10	0.800	1.24E-10
D_2	225	32	5.05E-26	0.838	4.23E-26
	275	16	1.44E-22	0.859	1.24E-22
	325	16	3.78E-20	0.861	3.25E-20
	375	16	2.51E-18	0.884	2.22E-18
	425	16	6.09E-17	0.880	5.36E-17
	500	16	2.45E-15	0.885	2.17E-15
	700	8	1.08E-12	0.875	9.47E-13
	1000	8	1.07E-10	0.850	9.09E-11

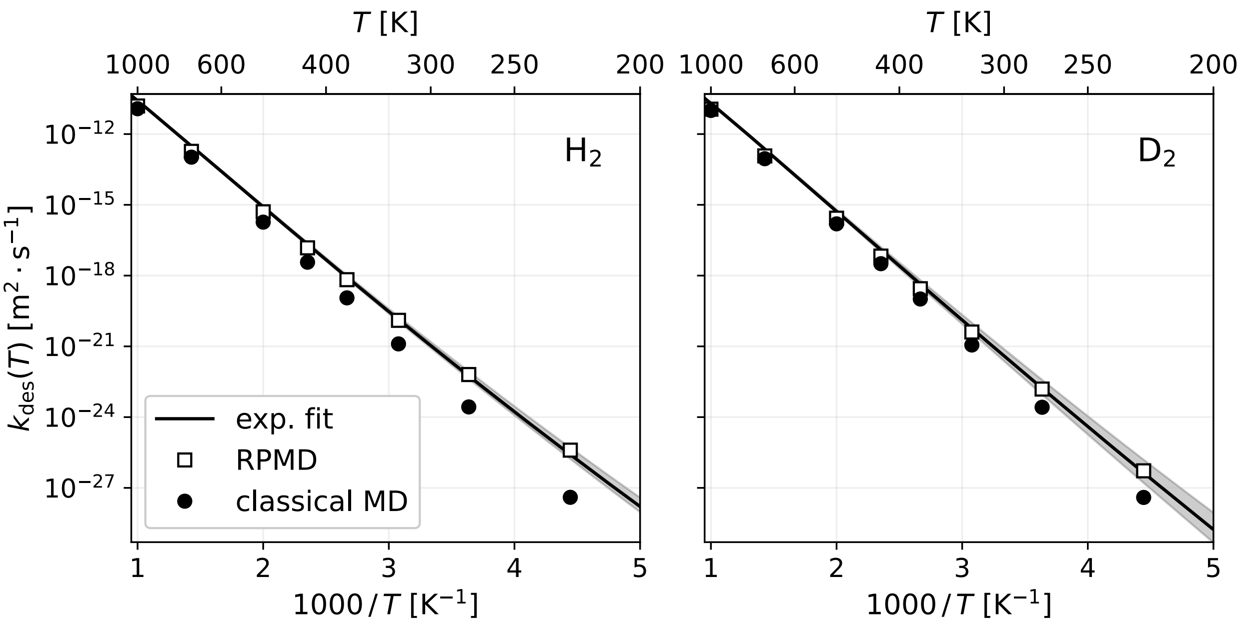

Figure S18: Rate constants for recombinative desorption of H_2 (left panel) and D_2 (right panel) from Cu(111) calculated with the RPMD rate theory (shown as squares) and classical MD (one bead RPMD, shown as circles). Experimentally derived rate constants are shown as solid lines with uncertainties indicated by the grey region around the solid lines. Note that the rate constants based on the classical MD and RPMD rate theory have been divided by 8 to account for the indistinguishability of the two atoms in the recombination reaction and the electronic spin effect.

S5.3. Derivation of Equation (S35)

It is well established that the classical flux-side correlation function $c_{fs}(t;s)$ is a real and odd function of t which is discontinuous at $t = 0$ and has a positive limit as t tends to zero from above [83]. This leads to a well-defined TST approximation to the rate constant,

$$k^{\mathrm{TST}}(s) = \frac{1}{Q_r(T)} c_{fs}(t \to 0_+; s) \,, \qquad \text{(S39)}$$

where

$$c_{fs}(t \to 0_+; s) = \frac{1}{(2\pi\hbar)^{3N}} \int d^{3N}\boldsymbol{p} \int d^{3N}\boldsymbol{q}\, e^{-\beta H(\boldsymbol{p},\boldsymbol{q})} \delta[s(\boldsymbol{q})] f_s(\boldsymbol{q}) \,. \qquad \text{(S40)}$$

N is the number of atoms in the system, $H(\boldsymbol{p},\boldsymbol{q}) = T(\boldsymbol{p}) + V(\boldsymbol{q})$ and $\beta = (k_{\mathrm{B}}T)^{-1}$. $f_s(\boldsymbol{q})$ can be computed as shown in equation (S41) [84].

$$f_s(\boldsymbol{q}) = \left\{ \sum_{i=1}^{3N} \frac{1}{2\pi\beta m_i} \left[\frac{\partial s(\boldsymbol{q})}{\partial q_i} \right]^2 \right\}^{\frac{1}{2}} \,, \qquad \text{(S41)}$$

where m_i is the atomic mass. $Q_r(T)$ is the classical partition function of the reactants per volume V.

$$Q_r(T) = \frac{1}{(2\pi\hbar)^{3N} V} \int d^{3N}\boldsymbol{p} \int d^{3N}\boldsymbol{q}\, e^{-\beta H_0(\boldsymbol{p},\boldsymbol{q})} \qquad \text{(S42)}$$

Here, $H_0(\boldsymbol{p},\boldsymbol{q}) = T(\boldsymbol{p})$ since the interaction between the reactants is considered negligible in the reactant asymptotic region.

Instead of using Cartesian coordinates, it is more convenient to work within the coordinates $\boldsymbol{q} = (X, Y, z_1, z_2, r, \varphi)$, with a Hamiltonian of the form:

$$H(\boldsymbol{p},\boldsymbol{q}) = \frac{P_X^2 + P_Y^2}{2M} + \frac{P_{z_1}^2}{2m_H} + \frac{P_{z_2}^2}{2m_H} + \frac{1}{2\mu_r}\left(p_r^2 + \frac{p_\varphi^2}{r^2} \right) + V(X, Y, z_1, z_2, r, \varphi) \,, \qquad \text{(S43)}$$

where, $M = 2m_{\mathrm{H}}$ and $\mu_r = m_{\mathrm{H}}/2$, where m_{H} is the mass of the hydrogen atom. z_1 (z_2) is the vertical distance between the atom 1 (2) and the first layer of the surface. The definition of the reactant asymptote relies on the following assumptions: First, we assume that the two hydrogen atoms adsorbed on the rigid surface can be treated as a two-dimensional system ignoring the surface corrugation. Second, we assume that the motion in the Z direction (along the surface normal) is completely decoupled from the in-plane motion and treated as static. Hence, $X = (x_1 + x_2)/2$, $Y = (y_1 + y_2)/2$, $z_1 = z_2 = Z_{\mathrm{ads}} = 0.89$ Å, $r = \sqrt{(x_1 - x_2)^2 + (y_1 - y_2)^2}$, $\tan\varphi = \frac{y_1 - y_2}{x_1 - x_2}$. The reactant reference Hamiltonian in equation (S43) can thus be simplified to:

$$H(\boldsymbol{p},\boldsymbol{q}) = \frac{P_X^2 + P_Y^2}{2M} + \frac{1}{2\mu_r}\left(p_r^2 + \frac{p_\varphi^2}{r^2}\right) + V(Z_{\mathrm{ads}}, r)\ . \tag{S44}$$

Choosing a circle

$$s_0(\boldsymbol{q}) = r_{\mathrm{ads}} - r = 0 \tag{S45}$$

with the radius r_{ads} as a dividing surface in the reactant reference plane, equation (S41) becomes:

$$f_{s_0}(\boldsymbol{q}) = \left\{\frac{1}{2\pi\beta\mu_r}\left[\frac{\partial s_0(\boldsymbol{q})}{\partial r}\right]^2\right\}^{\frac{1}{2}} = \left(\frac{1}{2\pi\beta\mu_r}\right)^{\frac{1}{2}}, \tag{S46}$$

which yields for the flux in equation (S40):

$$c_{fs}(t \to 0_+; s_0) = \frac{1}{(2\pi\hbar)^4}\left(\frac{1}{2\pi\beta\mu_r}\right)^{\frac{1}{2}} \int dP_X dP_Y dp_r dp_\varphi dX dY dr d\varphi\ e^{-\beta H(P_X, P_Y, p_r, p_\varphi, r)}\delta(r_{\mathrm{ads}} - r)\ . \tag{S47}$$

where the conservation of volume in the phase space of the system was considered. To integrate in the momentum space, we transform back from polar to Cartesian momenta and take advantage of the Gaussian integral:

$$\int_{-\infty}^{\infty} dx\, e^{-ax^2} = \left(\frac{\pi}{a}\right)^{\frac{1}{2}}, \tag{S48}$$

which leads to

$$c_{fs}(t \to 0_+; s_0) = \left(\frac{1}{2\pi\beta\mu_r}\right)^{\frac{1}{2}}\left(\frac{M}{2\pi\beta\hbar^2}\right)\left(\frac{\mu_r}{2\pi\beta\hbar^2}\right)\int dX dY dr d\varphi\ r e^{-\beta V(Z_{\mathrm{ads}}, r)}\delta(r_{\mathrm{ads}} - r) \tag{S49}$$

$$c_{fs}(t \to 0_+; s_0) = \left(\frac{1}{2\pi\beta\mu_r}\right)^{\frac{1}{2}}\left(\frac{M}{2\pi\beta\hbar^2}\right)\left(\frac{\mu_r}{2\pi\beta\hbar^2}\right)2\pi r_{\mathrm{ads}} e^{-\beta V(Z_{\mathrm{ads}}, r_{\mathrm{ads}})} S \tag{S50}$$

$$c_{fs}(t \to 0_+; s_0) = 2\pi r_{\mathrm{ads}} e^{-\beta V(Z_{\mathrm{ads}}, r_{\mathrm{ads}})}\left(\frac{1}{2\pi\beta\mu_r}\right)^{\frac{1}{2}} Q_0(T) \tag{S51}$$

Here, S is the reference area. The four-dimensional free-particle partition function $Q_0(T)$ follows equation (S52).

$$Q_0(T) = \left(\frac{M}{2\pi\beta\hbar^2}\right)\left(\frac{\mu_r}{2\pi\beta\hbar^2}\right) S = \left(\frac{m_{\mathrm{H}}}{2\pi\beta\hbar^2}\right)^2 S \tag{S52}$$

Finally, substituting equation (S51) into equation (S39) gives

$$k^{\mathrm{TST}}(s_0) = 2\pi r_{\mathrm{ads}} e^{-\beta V(Z_{\mathrm{ads}}, r_{\mathrm{ads}})} \left(\frac{1}{2\pi\beta\mu_r}\right)^{\frac{1}{2}} \frac{Q_0(T)}{Q_r(T)} \, . \tag{S53}$$

Assuming that the interaction energy between hydrogen atoms adsorbed at a distance r_{ads} is negligible, $V(Z_{\mathrm{ads}}, r_{\mathrm{ads}}) = 0$, the reactant partition function becomes:

$$Q_r(T) = \frac{1}{S} Q_0(T) \int d\varphi dr\, r = Q_0(T) \, . \tag{S54}$$

Therefore, the flux through the dividing surface, given by equation (S51), becomes

$$c_{fs}(t \to 0_+; s_0) = 2\pi r_{\mathrm{ads}} \left(\frac{1}{2\pi\beta\mu_r}\right)^{\frac{1}{2}} Q_0(T) \, , \tag{S55}$$

with the corresponding rate constant

$$k^{\mathrm{TST}}(s_0) = 2\pi r_{\mathrm{ads}} \left(\frac{1}{2\pi\beta\mu_r}\right)^{\frac{1}{2}} \, . \tag{S56}$$

The rate constant at the position of the reaction coordinate $\xi^{\ddagger}$ is calculated in the standard way by multiplying equation (S56) with a factor $e^{-\beta[W(\xi^{\ddagger})-W(0)]}$ [70].

S6. Determination of the Dissociative Adsorption Energy

Here, we extend the discussion of the model for desorption rates given in equation (3) of the main text to determine the dissociative adsorption energy, which we define as the energy of the H_2(D_2) gas-phase molecule minus the energy of two adsorbed H(D) atoms. Note that the adsorption energy is positive if two adsorbed H atoms are more stable than the gas-phase H_2 molecule.

The model requires several inputs, including the thermal adsorption rate constant k_{ads}, partition functions for gas-phase molecules and adsorbed atoms, and the adsorption energy. In sections S2 and S3, we described an accurate determination of k_{ads}, which serves as an input for this stage of the analysis. The gas-phase partition function can be precisely calculated using tabulated diatomic constants. In subsection S6.1, we show how we obtain the nuclear eigenstates and partition functions through a fully quantum mechanical treatment of the adsorbate. Consequently, the adsorption energy represents the only input necessary for the calculation of the recombinative desorption rate constant. We determine the adsorption energy by fitting equation (3) of the main text to previously published Temperature Programmed Desorption (TPD) data. The outcome of this fitting procedure can be considered as an experimental determination of the adsorption energy. Details about the TPD analysis can be found in subsection S6.2.

S6.1. Adsorbate Partition Function Modeling

Different approaches to determine adsorbate partition functions have been proposed and used in the literature [85-87]. However, most of these established models rely on approximations of the potential energy surface or on a classical treatment of the adsorbate, making them poor choices for adsorbates with low masses like hydrogen. Recently, we reported a method—Quantum Potential Energy Sampling (QPES)—that provides an accurate partition function of adsorbed hydrogen, taking into account the adsorbate's wave function delocalization, anharmonicity of the potential energy surface and electron spin [82,88]. We briefly describe how we have applied QPES to H and D adsorbed to Cu(111).

QPES assumes separated H atom motion for in and out of plane translation, a vibrationally adiabatic approximation that can be justified by the high out-of-plane stretching frequency of the hydrogen adsorbate [82,88]. The QPES partition function $q_{\mathrm{in-plane}}(T)$ is calculated with DFT in a two-dimensional in-plane potential on the Cu(111) surface, which we construct by placing hydrogen atoms at 10 different lateral positions in the irreducible part of the surface Wigner-Seitz cell. From these points we use symmetry considerations to reconstruct the in-plane interaction potential. At each in-plane position, we optimize the energy with respect to the z-coordinate allowing neighboring surface atoms to move, thereby creating a 2D energy grid in the plane. Harmonic frequencies along the z-direction were then calculated by displacing the adsorbates 0.01 Å from their optimized positions and solving the dynamical matrix with a finite difference scheme. We add the resulting harmonic vibrational zero-point energy to the interaction energies for each position in the plane to obtain the z-coordinate optimized and vibrational zero-point

energy corrected potential $V(x,y)$. For computational details of the DFT calculations see section S7.2.

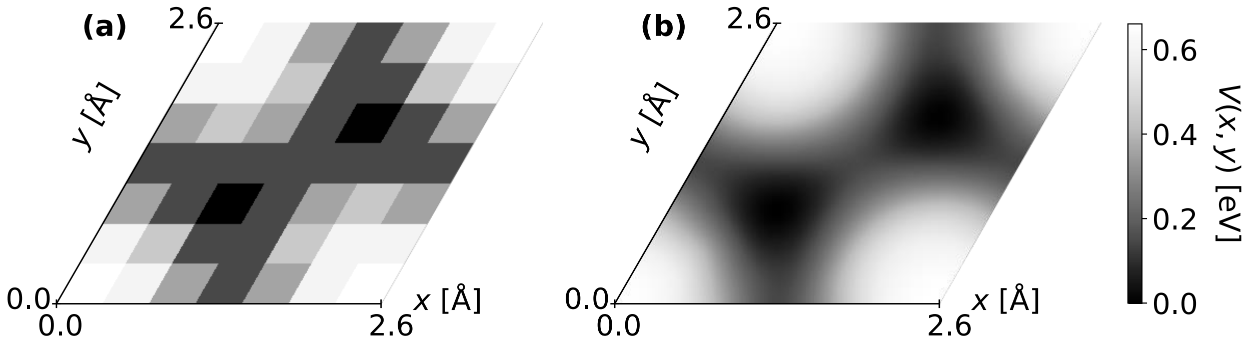

Figure S19: Vibrational zero-point corrected potential for the interaction hydrogen atoms with a Cu(111) surface. At each vertex of these plots, a copper atom (top site) is located. (a) Discrete values obtained from DFT using the RPBE exchange-correlation functional. (b) Bicubic interpolation of (a) with periodic boundary conditions.

Figure S19a displays the discrete values $V(x,y)$, where the energy of the most stable binding site (fcc hollow) is set to 0. For the partition function calculation, a bicubic interpolation with periodic boundary conditions of this discrete PES is evaluated for 80 points in each direction (see Figure S19b). For all calculations, we fix the distance between adjacent copper top sites on the surface to the experimental value of 2.56 Å [47]. A one-dimensional cut through this PES is shown in Figure S20. The D-Cu(111) in-plane interaction potential is obtained by adding zero-point energies for frequencies along the z-direction for D instead for H. We assume a $\sqrt{2}$ scaling between these frequencies. The in-plane potential $V(x,y)$ was constructed both with the RPBE and PBE functional, resulting in only minor differences in relative energies. There is only a weak influence of the choice of DFT functional on the QPES partition function.

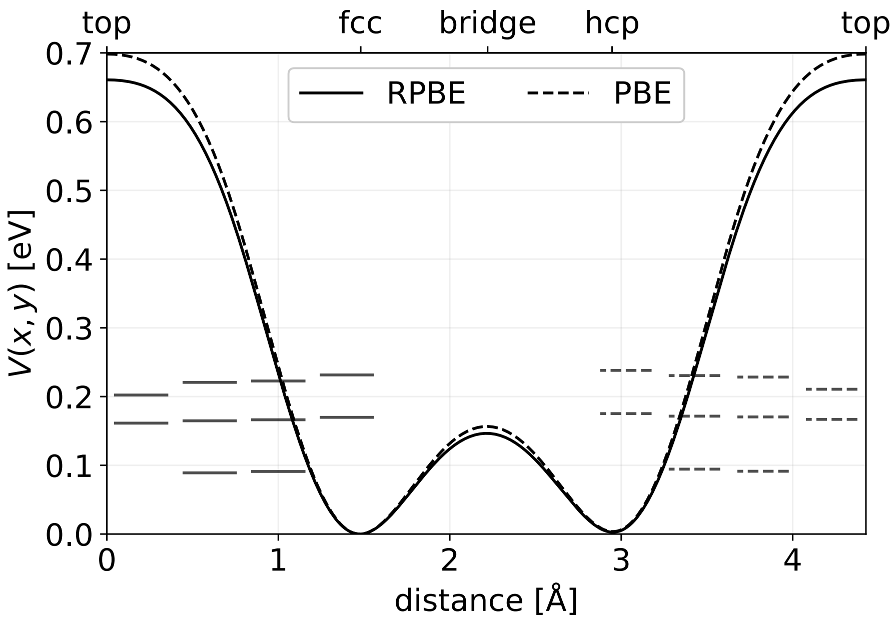

Figure S20: One-dimensional cut through the vibrational zero-point corrected H-Cu(111) interaction potential calculated with the RPBE (solid line) and PBE (dashed line) functional (see Figure S19(b) for a two-dimensional plot). Horizontal bars indicate the energies of the first 10 eigenstates.

We solved the two-dimensional Schrödinger equation numerically in the z-coordinate optimized potential $V(x,y)$ for H and D independently, yielding in-plane energy eigenvalues ϵ and eigenfunctions $\Psi(x,y)$. This procedure is equivalent to the one described in greater detail in reference 82. The fully quantum mechanical in-plane partition function can then be calculated as shown in equation (S57).

$$q_{\text{in-plane}}(T) = \sum_{i=0}^{\infty} \exp\left(-\frac{\epsilon_i - \epsilon_{i=0}}{k_\text{B}T}\right) \tag{S57}$$

To account for the vibration along the surface normal, a position-dependent harmonic vibrational partition function is constructed based on DFT calculated frequencies $\nu_z(x,y)$ for each position (x, y).

$$q_\text{vib}(x,y,T) = \left[1 - \exp\left(-\frac{h\nu_z(x,y)}{k_B T}\right)\right]^{-1} \tag{S58}$$

We average equation (S58) over the probability density $|\Psi_i(x,y)|^2$ of each eigenstate i and weight the result with the thermal population of eigenstate i. We repeat this procedure for all eigenstates with energies below 1 eV and sum all terms. We have shown in previous work that the adsorbate partition function $Q^{\text{H}^*}(T)$ must include an electronic spin degeneracy factor $q_\text{el} = 2$ (for H^* and D^*) [82].

$$Q^{\text{H}^*}(T) = q_\text{el} \times \sum_{i=0}^{\infty}\left[\exp\left(-\frac{\epsilon_i - \epsilon_{i=0}}{k_\text{B}T}\right) \times \frac{\iint q_\text{vib}(x,y,T)\ |\Psi_i(x,y)|^2\ \text{d}x\text{d}y}{\iint |\Psi_i(x,y)|^2\ \text{d}x\text{d}y}\right] \tag{S59}$$

S6.2. Fits to Temperature Programmed Desorption Data

In this section we show how we determined the adsorption energy from thermal adsorption rate constants and TPD data by applying the Detailed Balance Rate Model (DBRM) which was previously derived [82,89,90] and is summarized in equations (S60) and (S61).

$$k_{\mathrm{des}}^{\mathrm{X}_2}(T) = k_{\mathrm{ads}}^{\mathrm{X}_2}(T) \times \frac{Q_{\mathrm{gas}}^{\mathrm{X}_2}/V}{(Q^{\mathrm{X}^*}/A)^2} \exp\left(-\frac{\Delta E_{\mathrm{X}_2} - \alpha_{\mathrm{X}_2}\theta}{k_{\mathrm{B}}T}\right) \tag{S60}$$

$$\Delta E_{\mathrm{X}_2} = \Delta\varepsilon + \mathrm{ZPE}_{\mathrm{X}_2} - 2\ \mathrm{ZPE}_{\mathrm{X}}\ , \tag{S61}$$

where X represents the atoms H and D and θ symbolizes coverage. Only $\Delta\varepsilon$—the classical adsorption energy—and α_{X_2}—representing the coverage dependence of the adsorption energy associated with repulsive interactions between atoms [91]—are used to simultaneously fit TPD for H_2 and D_2 recombinative desorption. Adsorbate zero-point energies $\mathrm{ZPE}_{\mathrm{X}}$ were discussed in section S6.1, gas-phase molecular zero-point energies $\mathrm{ZPE}_{\mathrm{X}_2}$ are obtained from spectroscopy. Note that referencing the partition functions in equation (S60) to a unit volume V/unit area A yields the correct units for the recombinative desorption rate constant: $\mathrm{m}^2\ \mathrm{s}^{-1}$ or alternatively, $\mathrm{ML}^{-1}\ \mathrm{s}^{-1}$. Since the partition function refers to a unit cell of the Cu(111) surface containing one copper surface atom, we use $A \approx 5.66\ \mathrm{Å}^2$, derived from experimental lattice parameters [47]. This also establishes the surface coverage definition; an atom density of $1.77 \times 10^{15}\ \mathrm{cm}^{-2}$ corresponds to one monolayer (ML).

We consider four previously reported sets of TPD spectra for H_2 [91,92]—Figure S21(a) and (b)—and for D_2 [24,92]—Figure S21(c) and (d). Each data set contains TPD curves for several initial coverages θ_0, which must be known. Of course, the area under each TPD curve is proportional to θ_0, hence, the absolute value of θ_0 is only needed for one curve in the data set.

For the curves shown in panels (b) & (d) of Figure S21, θ_0 was given for the TPD curve, for which saturation with respect to H/D atom dosing was observed [92]. We scaled to the areas under the curves to obtain the other values of θ_0. A similar procedure was used to find θ_0 values for the data of panel S12(c) [24].

For curves shown in Figure S21(a), the authors reported for each TPD curve a hydrogen exposure I, based on a calibration of absolute pressures. We integrated the area under all TPD curves a plotted them versus I. See Figure S22. We utilized the known saturation coverage $\theta_{\mathrm{max}} = 0.5$ ML [91] of H on Cu(111) to determine the absolute initial coverages. This was done by fitting the data in Fig. S11 to the following equation.

$$\theta_0 = \theta_{\mathrm{max}} \frac{c \times (I/\mathrm{ML})^n}{1 + c \times (I/\mathrm{ML})^n}, \tag{S62}$$

where c and n are fitting parameters. Fitting c and n yielded the solid line shown in Figure S22 and $c = (8.1 \pm 0.2) \times 10^{-2}$ and $n = 0.89 \pm 0.02$. The initial coverages for curves in Figure S21a were then calculated from the reported values of I.

With knowledge of θ_0 for all TPD data, we numerically solved differential equation (S63).

$$\frac{\mathrm{d}\theta}{\mathrm{d}T} = -\frac{2}{\beta} k_{\mathrm{des}}(T) \times \theta^2 \tag{S63}$$

Here, the heating rate β is 3 K/s for panels (a), (b), (d) and 2 K/s for panel (c) in Figure S21 [24,91,92].

The desorption rate, $k_{\mathrm{des}}\theta^2(T)$, can be computed from the experimentally observed pressure increase versus T with appropriate conversion of units. This requires fitting a single amplitude parameter for each set of TPD curves, as well as the classical zero-coverage adsorption energy $\Delta\varepsilon$ and its coverage dependence parameter α. This was done separately for data of panel (a)-(d).

This local fitting procedure is employed due to the slightly different methods and resulting confidence intervals associated with the determination of initial coverages across these different studies. We assign uncertainties of 10% (study (a), (b), (d)) or 20% (study (c)) to these initial coverages [92,93]. To ensure the validity of the applied linear coverage dependence of the energy, we restrict the fitting procedure to curves with initial coverages $\theta_0 \leq 0.3$ ML. The fits to are shown as red (H_2) and blue (D_2) solid lines in Figure S21(a-d) and the derived values of $\Delta\varepsilon$ and α are shown in Figure S21(e) and (f).

Uncertainties in the TPD modeling arise from uncertainties in the initial coverages, the experimentally derived adsorption rate constant, and the choice of DFT functional used to calculate the adsorbate PES. The errors associated with the first two are represented by the vertical error bars in Figure S21e and f. We explicitly show separate results for fits using the PBE and RPBE PES to demonstrate the insensitivity of this analysis to the choice of the DFT functional. Weighting results obtained for panels (a)-(d) with the number of data points and the fit quality, we obtain $\Delta\varepsilon = 0.348^{+0.028}_{-0.023}$ eV and $\alpha = 0.208^{+0.049}_{-0.072}$ eV·ML^{-1}. Zero-point energy corrected adsorption energies are $\Delta E_{\mathrm{H}_2} = 0.311^{+0.028}_{-0.023}$ eV and $\Delta E_{\mathrm{D}_2} = 0.320^{+0.028}_{-0.023}$ eV.

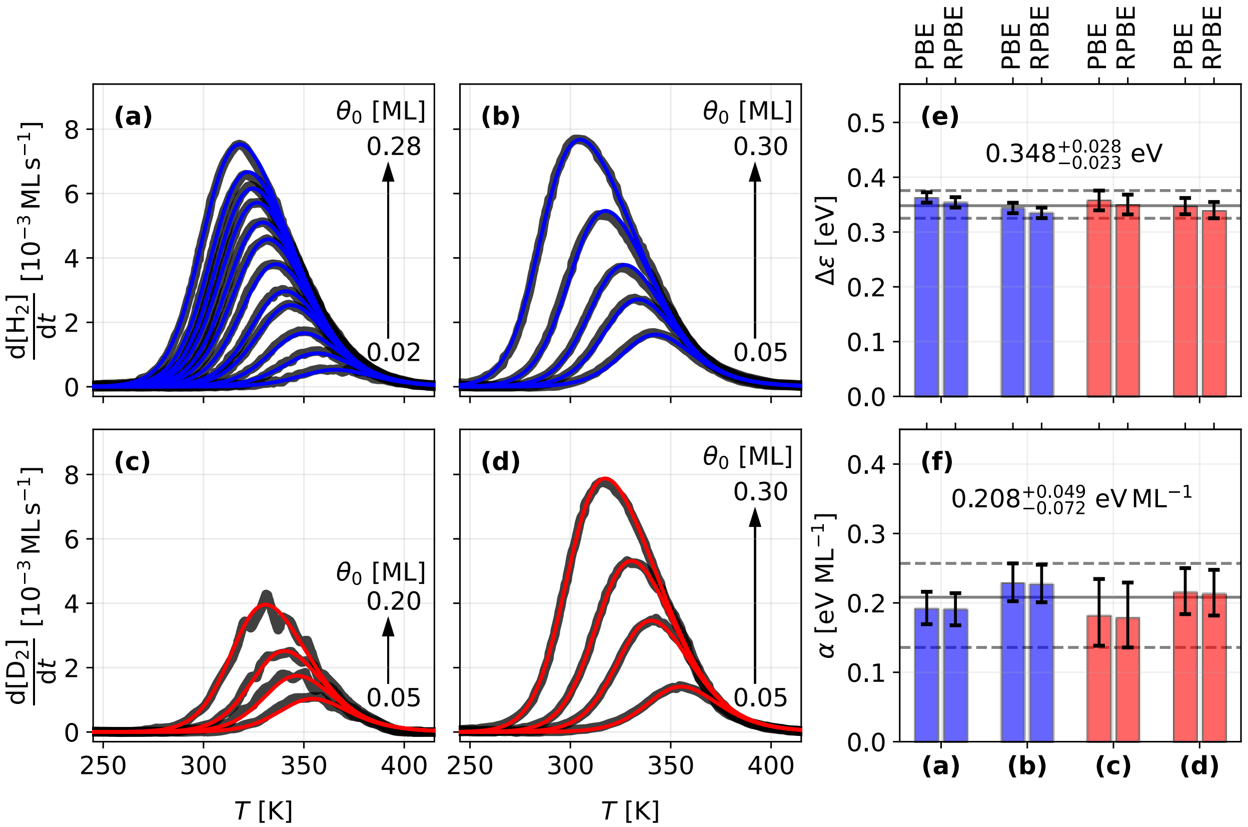

Figure S21: Panels (a)-(d): Experimental TPD spectra (black line) and best fit for hydrogen/deuterium (blue/red solid line). Experimental data was taken from (a): reference 91, (b), (d): reference 92, (c): reference 24. We fit for each panel the classical adsorption energy $\Delta\varepsilon$ and the parameter α accounting for its linear decrease with increasing coverage. Panels (e), (f): Fit results and uncertainties, explicitly shown using the PBE and RPBE functional for construction of the adsorbate PES.

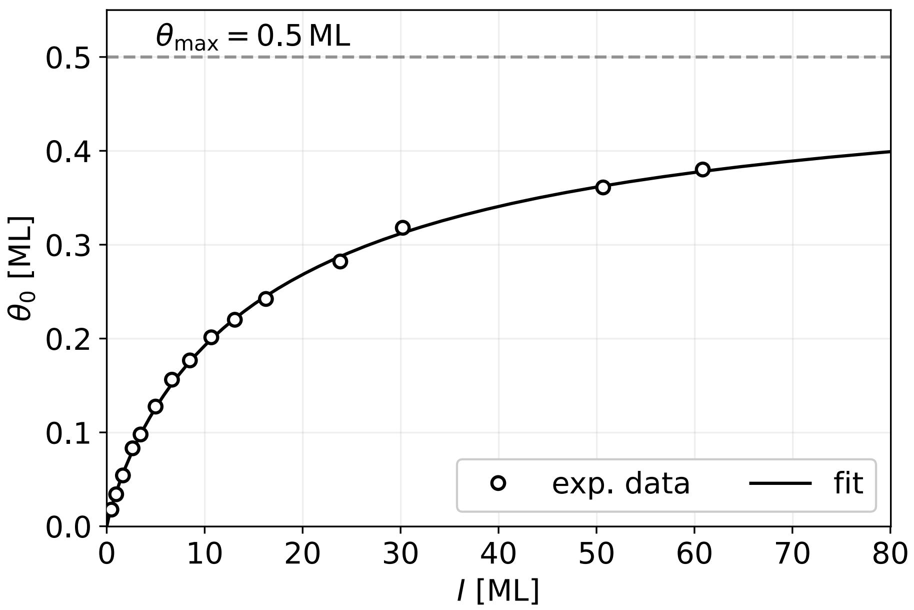

Figure S22: Areas under experimental TPD curves, being proportional to the initial coverage θ_0 in the TPD experiment, show a saturation with respect to increasing exposures (denoted as I). Experimental values from reference 91 (circles) were fitted with equation (S62) to yield the solid line. Setting the absolute saturation value of the fit to the experimental saturation coverage θ_{max} for hydrogen atoms on Cu(111)[91] allows to determine initial TPD coverages that are required for the model shown in Figure S21a.

S6.3. Estimation of the Adsorption Energy from Eley-Rideal Reactions

In this section, we independently determine a rigorous upper bound to the classical adsorption energy $\Delta\varepsilon$ of hydrogen on Cu(111) through analysis of Eley-Rideal surface reactions and thermodynamic considerations. Rettner and Auerbach determined the population distribution of ro-vibrational states for HD formed when H(D)-atoms collide with a D(H)-covered Cu(111) surface [94]. The highest observed internal energy of the product HD estimated from their results is $E_{\mathrm{max}}^{\mathrm{H-D_{ad}}} = 2.08$ eV when H atoms collide with D-covered Cu(111) and $E_{\mathrm{max}}^{\mathrm{D-H_{ad}}} = 2.23$ eV when D atoms collide with H-covered Cu(111) [94]. Assuming no translational energy in the HD product, no excitation of surface phonons or electron-hole pairs, but accounting for the incidence translation energy of the atoms $E_{\mathrm{trans}} = 0.07$ eV [94], we may equate $\left(E_{\mathrm{max}}^{\mathrm{H-D_{ad}}} - E_{\mathrm{trans}}\right)$ and $\left(E_{\mathrm{max}}^{\mathrm{D-H_{ad}}} - E_{\mathrm{trans}}\right)$ to the two Eley-Rideal reactions' energies.

Equation (S64) shows how to obtain the classical atomic adsorption energy $\Delta\varepsilon_{\mathrm{at}}$ from the two Eley-Rideal reactions. The fact that the two values are not equal reflects errors and uncertainties in the experiment and assumptions.

$$\Delta\varepsilon_{\mathrm{at}} \leq \begin{cases} D_0^{\mathrm{HD}} + \mathrm{ZPE_H} - \left(E_{\mathrm{max}}^{\mathrm{D-H_{ad}}} - E_{\mathrm{trans}}\right) = 2.52\ \mathrm{eV} \\ D_0^{\mathrm{HD}} + \mathrm{ZPE_H} - \left(E_{\mathrm{max}}^{\mathrm{H-D_{ad}}} - E_{\mathrm{trans}}\right) = 2.63\ \mathrm{eV} \end{cases} \qquad \text{(S64)}$$

Here, the HD dissociation energy ($D_0^{\mathrm{HD}} = 4.520$ eV) is known from the literature [95] and the zero-point energy of adsorbed H ($\mathrm{ZPE_H}$) and D ($\mathrm{ZPE_D}$) atoms are obtained as described in section S6.1.The thermodynamic cycles associated with these equations are shown in Figure S23 and equation (S64).

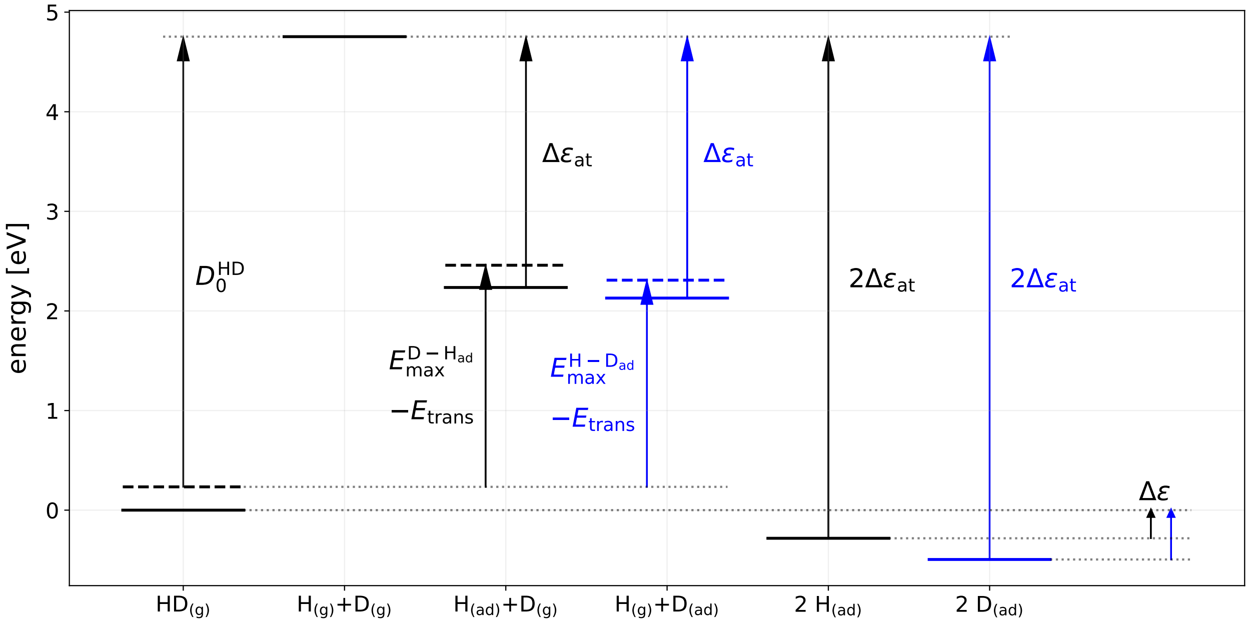

Figure S23: Thermodynamic cycle for estimation of the classical adsorption energy Δε of hydrogen on Cu(111). This estimation was performed for H_2 (black) and D_2 (blue) independently. Gas-phase species are indexed with (g) and adsorbed atoms as (ad).

With knowledge of $\Delta\varepsilon_{\text{at}}$, the classical dissociative adsorption energy $\Delta\varepsilon$ can be calculated according to equation (S65). This requires knowledge of the vibrational zero-point energy of the HD molecule ($\text{ZPE}_{\text{HD}} = 0.234$ eV [96]).

$$\Delta\varepsilon \leq 2\Delta\varepsilon_{\text{at}} - D_0^{\text{HD}} - \text{ZPE}_{\text{HD}} = \begin{cases} 0.28 \text{ eV} \\ 0.50 \text{ eV} \end{cases} \tag{S65}$$

The two values obtained reflect independent determination from the two Eley-Rideal reactions and the uncertainty of this analysis. Due to the assumptions made, $E_{\text{max}}^{\text{H-D}_{\text{ad}}}$ and $E_{\text{max}}^{\text{D-H}_{\text{ad}}}$ are rigorous lower bounds making $\Delta\varepsilon_{\text{at}}$ and $\Delta\varepsilon$ rigorous *upper* bounds.

To summarize, state-resolved detection of the Eley-Rideal reaction products clearly show that the maximum energy for dissociative adsorption of hydrogen on copper surfaces is $\Delta\varepsilon = 0.39 \pm 0.11$ eV at the experimental coverage of $\theta = 0.5$ ML [94]. If extrapolated to the low coverage limit—see section S6.2—we obtain an upper limit of 0.49 ± 0.11 eV. This upper limit is consistent with the present work (see Figure S24). A previously reported value derived from isotherm measurements is not consistent with this upper bound, a topic to which we turn in section S6.4.

S6.4. Previous Determinations of the Adsorption Energy

Adsorption Isotherms

The isosteric heat of hydrogen adsorption on Cu(111) was previously reported to be $\Delta H_{\text{ads}} = (0.58 \pm 0.08)$ eV based on isothermal adsorption measurements experiments at $T \approx 360$ K[23]. The adsorption *enthalpy* is temperature dependent—see equation (S66)—and can only be compared to ΔE_{H_2} at zero Kelvin—see equation (S67).

$$\Delta H_{\text{ads}}(T) = U_{\text{H}_{2(\text{g})}}(T) - 2U_{\text{H}_{(\text{ad})}}(T) + k_{\text{B}}T \tag{S66}$$

$$\Delta H_{\text{ads}}(0\ \text{K}) = U_{\text{H}_{2(\text{g})}}(0\ \text{K}) - 2U_{\text{H}_{(\text{ad})}}(0\ \text{K}) = \Delta E_{\text{H}_2} \tag{S67}$$

In these equations, U represents the internal energy, gas-phase and adsorbed species are indicated with (g) and (ad), respectively.

Subtracting equation (S66) from (S67) yields the adsorption energy ΔE_{H_2} as follows:

$$\Delta E_{\text{H}_2} = \Delta H_{\text{ads}}(T) - \left[U_{\text{H}_{2(\text{g})}}(T) - U_{\text{H}_{2(\text{g})}}(0\ \text{K})\right] + 2\left[U_{\text{H}_{(\text{ad})}}(T) - U_{\text{H}_{(\text{ad})}}(0\ \text{K})\right] - k_{\text{B}}T \tag{S68}$$

The difference between the internal energy at a given temperature T and at 0 K can be derived from the partition function of the respective species, equation (S69).

$$U(T) - U(0\ \text{K}) = k_{\text{B}}T^2 \frac{\partial \ln(Q)}{\partial T} \tag{S69}$$

For adsorbed hydrogen atoms, we use the QPES partition function given in section S6.1. The gas-phase partition function is based on spectroscopic constants for diatomic molecules from the NIST database[46]. This allows us to extract from the isotherm measurements a rigorous adsorption energy $\Delta E_{\text{H}_2,\text{isoth.}} = (0.52 \pm 0.08)$ eV and, after zero-point energy correction based on equation (S61), a classical adsorption energy of $\Delta\varepsilon_{\text{isoth.}} = (0.55 \pm 0.08)$ eV. These values are larger than the Eley-Rideal upper limit for the adsorption energy (0.49 $\pm$ 0.11 eV, see section S6.3 and Figure S24).

We attribute the quantitative error of this determination to difficulties associated with surface impurities, which become more important for isothermal adsorption measurements on systems with low adsorption probabilities, such as hydrogen on Cu(111). Consider that in the isotherm study, pressures in the range of 400 Pa – 2000 Pa were required to establish detectable stationary hydrogen coverages [23]. Errors will arise, if impurities adsorb and block H-atom surface binding sites [23]. In fact, the authors reported evidence for accumulation of impurities on their sample but claimed that the use of high pressures removes these impurities by chemical reaction with hydrogen and subsequent product desorption [23]. However, no additional experimental evidence for impurity removal was presented.

TPD

The dissociative adsorption energy of H_2 on Cu(111) $\Delta\varepsilon$ was also previously reported based on TPD experiments as (0.15 ± 0.01) eV [24] which is $\approx$ 0.2 eV smaller than the value of the present work (see Figure S24). The TPD data in question was presented by us in section S6.2 and shown to be consistent with $\Delta\varepsilon = 0.348^{+0.028}_{-0.023}$ eV. This discrepancy arises from simplifications in the previous analysis of TPD data and erroneous conclusions. Specifically, the authors derived from the Arrhenius activation energy for desorption the energy $\Delta\varepsilon$ [24]. By equating this activation energy to the barrier height for desorption, an error of $\approx$ 0.2 eV was made, which is mainly caused by tunneling.

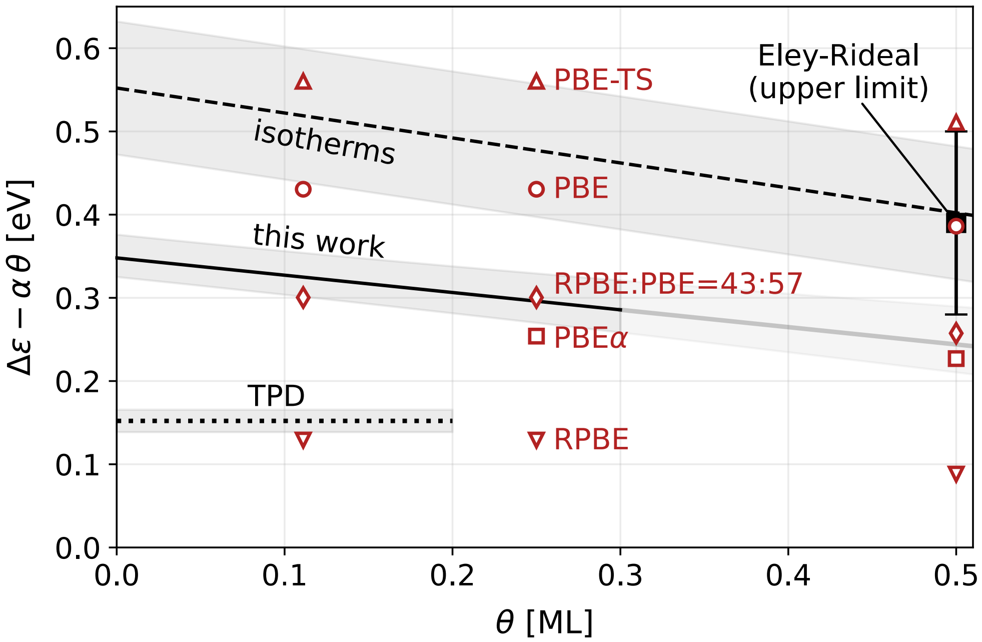

Figure S24: Classical adsorption energy vs. surface coverage θ: This work (black solid line) with uncertainties indicated by the gray shaded region, valid below at least θ≈ 0.3 ML. Prior reported results derived from refs. 23 and 24 are indicated with the dashed and dotted lines. A rigorous upper limit from Eley-Rideal reactions is shown as the solid square (see section S6.3). Results of DFT calculations with different functionals are shown as red symbols (see SI section S7.1 for computational details).

S7. Density Functional Theory (DFT) Calculations

S7.1. Hydrogen Dissociation: Adsorption Barrier and Energy

The DFT calculations were performed with the aid of the Vienna ab-initio simulation package VASP 5.4.4 [57,97,98]. The k-point grid of the Brillouin zone has been sampled according to the scheme proposed by Monkhorst and Pack [99]. Partial occupancies were modeled with the method of Methfessel and Paxton ($N = 2$) [100]. A smearing width σ of 0.2 eV and an energy cutoff of 450 eV were used. For the description of electronic exchange and correlation effects the generalized gradient approximation (GGA) PBE [101] and RPBE [102] functionals have been used. The influence of van der Waals interactions was tested by applying van der Waals corrections in an *a posteriori* manner as proposed by Tkatchenko and Scheffler [103] to the PBE calculations (PBE-TS) as well as by performing calculations with the non-local van-der-Waals optB86b [104] and vdW-DF2 [105] functionals. We also employed calculations with three distinct versions of the specific reaction parameter (SRP) functional [27,106]. In the original work, a weighted average of two GGA exchange correlation functionals, PW91 and RPBE, was used and resulted in the "SRP43" functional [27]. Here, we follow a later report [28,40] that averages PBE and RPBE exchange correlation functionals to yield the SRP48 and SRP50 functionals. The value of the mixing parameter determines the percentage of RPBE contribution into the exchange correlation energy. We tested three values of the mixing parameter: 43, 48, and 50, but in all cases mixed RPBE with PBE. To indicate the difference to the original functional form of SRP43, we will use the term RPBE:PBE=43:57.

The Cu(111) surface was modelled by a (2×2) slab with 4 layers to calculate adsorption energies and adsorption barrier heights. We additionally employed calculations using a (3×3) slab with 4 layers to calculate the adsorption energy at lower coverage. The k-point grid of the Brillouin zone has been sampled as a (15 × 15 × 1) mesh for the 2×2 slab and as a (10 × 10 × 1) mesh for the 3×3 slab. To prevent interactions of the slabs with their periodic images, a vacuum layer of 15 Å was introduced into the simulation cell perpendicular to the surface. The optimized lattice constants used for calculation of bulk coordinates are listed in Table S12. The DFT calculations presented in this section were performed using a more conservative set of computational parameters than in section S5 to minimize possible inaccuracies in forces and frequencies and to calculate the effect of the DFT functional reliably.

When relaxing the slabs, positions of atoms in the bottom layer were fixed at bulk positions, the other layers were relaxed until the norms of all forces were smaller than $0.02\ \mathrm{eV/Å}$. The optimization of the electronic energy was stopped if the total free energy change between two steps was smaller than 10^{-5} eV. The energy of the optimized slab is denoted as $\varepsilon_{\mathrm{slab}}$ in the following.

To calculate hydrogen's dissociative adsorption energy $\Delta\varepsilon$, one hydrogen atom was placed at its most stable binding site (face-centered hollow site, fcc, for all employed functionals) on the surface of the (3×3) slab and the (2×2) slab (in a different calculation). Due to the size of these unit cells, the adsorption energies correspond to H atom coverages of 0.11 ML and 0.25 ML. Positions of the H atom and the Cu atoms within the top three layers were optimized until the above-mentioned

convergence criteria were met, resulting in the energy $\varepsilon_{\mathrm{slab+H_{(ad)}}}$. The adsorption energy is calculated according to equation (S70).

$$\Delta\varepsilon(0.11\ \&\ 0.25\ \mathrm{ML}) = \varepsilon_{\mathrm{slab}} + \varepsilon_{\mathrm{slab+H_{2(g)}}} - 2\varepsilon_{\mathrm{slab+H_{(ad)}}}\ , \tag{S70}$$

where $\varepsilon_{\mathrm{slab}}$ is the energy of the clean relaxed slab and $\varepsilon_{\mathrm{slab+H_{2(g)}}}$ is the energy of the relaxed slab with the H_2 molecule in its equilibrium geometry in the gas phase (here, at 7.5 Å distance from the surface). Both energies were calculated using the same conditions and cell sizes as for $\varepsilon_{\mathrm{slab+H_{(ad)}}}$. Results are listed in Table S12.

To determine the dissociative adsorption energy at 0.50 ML coverage, we placed two hydrogen atoms in adjacent fcc sites of the (2×2) unit cell. Again, we optimize the coordinates of both H atoms and the upper three layers of the slab. The corresponding adsorption energy at 0.50 ML is calculated according to equation (S71).

$$\Delta\varepsilon(0.50\ \mathrm{ML}) = \varepsilon_{\mathrm{slab+H_{2(g)}}} - \varepsilon_{\mathrm{slab+2H_{(ad)}}}\ , \tag{S71}$$

where $\varepsilon_{\mathrm{slab+2H_{(ad)}}}$ is the energy of the optimized configuration with hydrogen atoms located in adjacent fcc hollow sites. Results are shown in Table S12.

Table S12: Lattice constant of copper (a) and classical adsorption energy ($\Delta\varepsilon$) of hydrogen on Cu(111). DFT-derived adsorption energies were calculated according to equations (S70) and (S71) and refer to the most stable binding site (fcc hollow). From a linear fit to $\Delta\varepsilon$ at three distinct coverages, we determined the coverage dependence α of the adsorption energy. A comparison to experimental lattice parameters/energetics from fits to TPD spectra is given, see section S6.2 for more details.

DFT functional	a [Å]	$\Delta\varepsilon$ (0.50 ML) [eV]	$\Delta\varepsilon$ (0.25 ML) [eV]	$\Delta\varepsilon$ (0.11 ML) [eV]	α [eV ML^{-1}]
PBE-TS	3.5335	0.511	0.560	0.570	0.157
PBE	3.6273	0.386	0.431	0.433	0.127
optB86b	3.5940	0.365	0.410	0.416	0.137
RPBE:PBE =43:57	3.6464	0.257	0.300	0.305	0.129
SRP48	3.6471	0.242	0.286	0.289	0.126
SRP50	3.6480	0.236	0.280	0.283	0.126
RPBE	3.6707	0.088	0.129	0.134	0.124
vdW-DF2	3.7445	−0.198	−0.162	−0.202	0.007
experimental	3.6149[47]	$0.244^{+0.046}_{-0.034}$	$0.296^{+0.033}_{-0.026}$	$0.325^{+0.029}_{-0.024}$	$0.208^{+0.049}_{-0.072}$

The geometry with two H atoms in adjacent fcc sites on the surface of the (2×2) slab served as the final state for calculation of the minimum energy path (MEP) for hydrogen dissociation, while the initial geometry is a hydrogen molecule 4.5 Å away from the surface. This geometry was optimized by fixing the positions of the bottom layer of the slab and the z-coordinate of both H atoms. Between initial and final state, 5 images are constructed. The MEP is found by employing the climbing image nudged elastic band method (CI-NEB) [107] in combination with the above-mentioned convergence criteria. During the CI-NEB calculation, all atoms except the bottom layer of the slab were allowed to move. The image with maximum energy $\varepsilon_{\mathrm{max}}$ is used to calculate the classical barrier $\varepsilon^{\ddagger}_{\mathrm{ads}}$ for H_2 adsorption:

$$\varepsilon^{\ddagger}_{\mathrm{ads}} = \varepsilon_{\mathrm{max}} - \varepsilon_{\mathrm{slab+H_2(g)}} \,. \tag{S72}$$

For the sake of consistency with the definition of the adsorption energy we reference the barrier height to the energy $\varepsilon_{\mathrm{slab+H_2(g)}}$ of the H_2 molecule at 7.5 Å distance from the surface. In Figure S25 and Figure S26 we show geometries and energies of all images, respectively. Subsequently, we displaced hydrogen atoms of the highest energy image by 0.015 Å out of the optimized geometry to construct and solve the dynamical matrix in terms of a finite difference scheme. In

this calculation, positions of all copper atoms were fixed. The resulting frequencies are shown together with values for $\varepsilon^{\ddagger}_{\mathrm{ads}}$ in Table S13. Note that barrier heights and transition state frequencies were only calculated for the (2×2) slab.

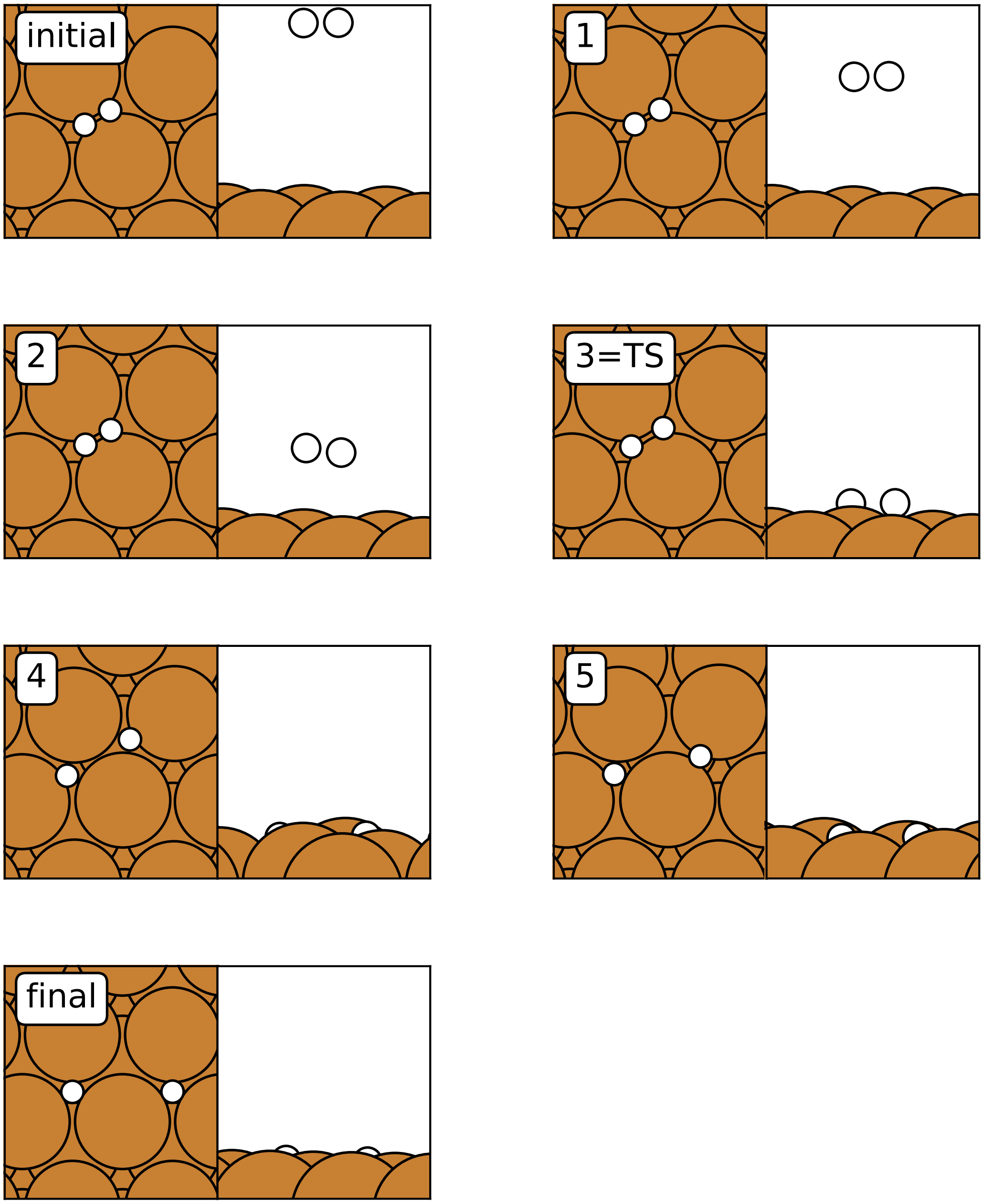

Figure S25: Minimum energy path for hydrogen dissociation at Cu(111) bridge sites calculated with the climbing image nudged elastic band method [107] and the PBE exchange correlation functional. For computational details see section S7.1.

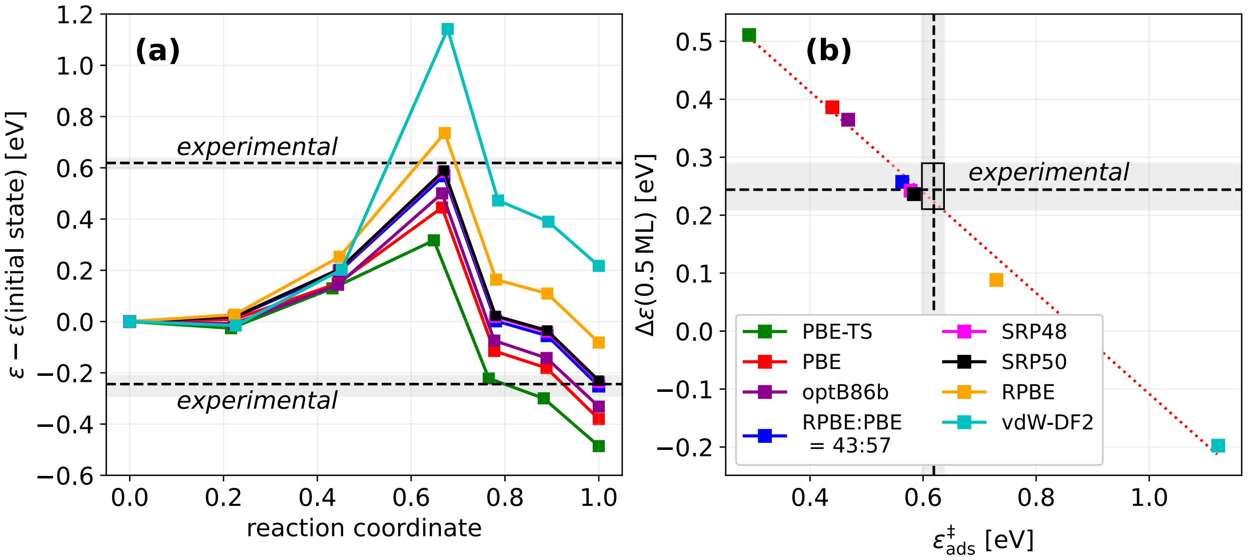

Figure S26: (a) Energies of the images along the minimum energy path for hydrogen dissociation at Cu(111) bridge sites calculated with the climbing image nudged elastic band method[107]. Figure S25 shows geometries of these images. The calculations were performed with different exchange correlation functionals as indicated in the legend (see (b), legend applies to (a) and (b)). For computational details see section S7.1. (b) Adsorption energies $\Delta\varepsilon(0.5\ ML)$ and adsorption barriers $\varepsilon^{\ddagger}_{ads}$ calculated with different exchange correlation functionals according to equations (S70) and (S71). The red dotted line shows a linear fit to the squares. The black rectangle indicates a 95% confidence interval for the experimental values. The linear dependence between adsorption barrier and energy are indicative of the Brønsted-Evans-Polanyi (BEP) relationship.

Table S13: DFT calculated classical barrier heights $\varepsilon^{\ddagger}_{ads}$ and transition state frequencies for H_2 dissociation on Cu(111). In all cases, hydrogen dissociates on a bridge site, yielding hydrogen atoms in the adjacent hollow sites. For computational details, see section S7.1. The last column shows the harmonic transition state partition function at 300 K, which was calculated using equation (S21).

functional	$\varepsilon^{\ddagger}_{\mathrm{ads}}$ [eV]	$\tilde{\nu}_i$ [cm^{-1}] $i=1$	$i=2$	$i=3$	$i=4$	$i=5$	$\nu^{\ddagger}$	$Q_{\mathrm{TS}}^{\mathrm{H_2}}$ (300 K)
PBE-TS	0.292	1994	954	688	301	183	399i	2.354
PBE	0.440	1857	959	726	302	245	443i	1.971
optB86b	0.468	1566	974	738	309	298	648i	1.770
RPBE:PBE =43:57	0.563	1651	982	761	313	310	569i	1.724
SRP48	0.578	1679	979	761	311	308	550i	1.733
SRP50	0.584	1622	986	766	321	313	588i	1.696
RPBE	0.730	1457	996	787	362	320	721i	1.597
vdW-DF2	1.122	1020	929	736	386	303	1310i	1.626

In Figure S26(b), we show that adsorption barriers and adsorption energies calculated with various exchange correlation functionals are correlated ($R^2 = 0.994$) in a manner reminiscent of the Brønsted-Evans-Polanyi (BEP) relationship [108]. It was demonstrated previously that for a GGA-functional, DFT predicts reaction barriers and reaction energies that follow the BEP trend, both for different types of surface reactions and different catalysts [109-113]. Here, we observe a BEP relationship for one specific molecule-surface pair, but different descriptions of the exchange-correlation energy. The linear fit (red dotted line Figure S26(b)) to the DFT calculated adsorption barriers and energies intersects the experimental values (black rectangle in Figure S26(b)) reported in this work within uncertainties. This suggests that different exchange correlation functionals can be mixed on a semi-empirical level to yield an accurate description of both the adsorption barrier height and the energy for this system. The only prerequisite for a successful comparison to the experiment is to choose at least one functional which gives a lower and one giving an upper limit to the experimental value. As evident from Figure S25(b), the PBE and RPBE functionals fulfill this condition. Therefore, the previously introduced weighted linear combination of both functionals [28], SRPX, where X is the percentage of RPBE in the weighted linear combination, can provide a chemically accurate description of both the adsorption barrier and energy. In this case, X can be tuned to reproduce either the experimental adsorption energy or the adsorption barrier height, as done previously resulting in $\mathrm{X} = 43 - 50$ [29].

S7.2. Calculation of the in-plane Interaction Potential

The DFT calculations were performed with the aid of the Vienna ab-initio simulation package VASP 5.3.5 [57,97,98]. The k-point grid of the Brillouin zone has been sampled as a (4 × 4 × 1) mesh according to the sampling scheme proposed by Monkhorst and Pack [99]. Partial occupancies were modeled with the method of Methfessel and Paxton ($N = 1$) [100]. The smearing width σ was set to 0.2 eV. An energy cutoff of 450 eV was used. For the description of electronic exchange and correlation effects, two different functionals within the generalized gradient approximation (GGA) have been used: the PBE [101], RPBE [102]. The surfaces were modeled by a (3×3) slab with 6 layers, where the two bottom layers were kept fixed. The other layers were relaxed until the norms of all forces were smaller than $0.05\ \mathrm{eV/Å}$. Periodic boundary conditions were applied in all three directions. The optimization of the electronic energy was stopped if the total free energy change between two steps was smaller than 10^{-5} eV. To prevent interactions of the copper slab with its periodic images in perpendicular direction, a vacuum layer of 15 Å was introduced into the simulation cell. Convergence of the in-plane interaction potential with respect to the chosen set of computational parameters was reached and verified.

S8. Additional References

1 Levine, R. D. *Molecular reaction dynamics*. (Cambridge University Press, 2009).

2 Rettner, C., Michelsen, H. & Auerbach, D. Quantum-state-specific dynamics of the dissociative adsorption and associative desorption of H_2 at a Cu(111) surface. *The Journal of Chemical Physics* **102**, 4625-4641 (1995).

3 Rettner, C. T., Michelsen, H. A. & Auerbach, D. J. From quantum-state-specific dynamics to reaction rates: the dominant role of translational energy in promoting the dissociation of D_2 on Cu(111) under equilibrium conditions. *Faraday Discussions* **96**, 17-31 (1993).

4 Donald, S. B. & Harrison, I. Rice–Ramsperger–Kassel–Marcus Simulation of Hydrogen Dissociation on Cu (111): Addressing Dynamical Biases, Surface Temperature, and Tunneling. *The Journal of Physical Chemistry C* **118**, 320-337 (2014).

5 Abbott, H. & Harrison, I. Seven-dimensional microcanonical treatment of hydrogen dissociation dynamics on Cu (111): Clarifying the essential role of surface phonons. *The Journal of Chemical Physics* **125**, 024704 (2006).

6 Kwan, T. & Kujirai, M. Absolute Rate of the Chemisorption of Hydrogen on Reduced Copper. *The Journal of Chemical Physics* **19**, 798-799 (1951).

7 Mikovksy, R. J., Boudart, M. & Taylor, H. S. Hydrogen-deuterium exchange on copper, silver, gold and alloy surfaces. *Journal of the American Chemical Society* **76**, 3814-3819 (1954).

8 Cadenhead, D. & Wagner, N. Hydrogen sorption and exchange on reduced-oxide nickel and copper. *Journal of Catalysis* **21**, 312-320 (1971).

9 Alexander, C. & Pritchard, J. Chemisorption of hydrogen on evaporated copper films. *Journal of the Chemical Society, Faraday Transactions 1: Physical Chemistry in Condensed Phases* **68**, 202-215 (1972).

10 Kiyomiya, M., Momma, N. & Yasumori, I. The Kinetics and Mechanism of Hydrogen Adsorption and Hydrogen–Deuterium Equilibration on the Copper Surface. *Bulletin of the Chemical Society of Japan* **47**, 1852-1857 (1974).

11 Balooch, M. & Stickney, R. Angular distributions of H_2 desorbed from the (100),(110), and (111) faces of copper crystals. *Surface Science* **44**, 310-320 (1974).

12 Hayden, B. & Lamont, C. Coupled translational-vibrational activation in dissociative hydrogen adsorption on Cu (110). *Physical Review Letters* **63**, 1823 (1989).

13 Campbell, J. & Campbell, C. T. The dissociative adsorption of H_2 and D_2 on Cu (110): activation barriers and dynamics. *Surface Science* **259**, 1-17 (1991).

14 Hayden, B. & Lamont, C. Vibrational and translational energy partition and the barrier to dissociative H_2 and D_2 adsorption on Cu (110). *Surface Science* **243**, 31-42 (1991).

15 Sandoval, M. J. & Bell, A. T. Temperature-programmed desorption studies of the interactions of H_2, CO, and CO_2 with Cu/SiO_2. *Journal of Catalysis* **144**, 227-237 (1993).

16 Rasmussen, P., Holmblad, P., Christoffersen, H., Taylor, P. & Chorkendorff, I. Dissociative adsorption of hydrogen on Cu (100) at low temperatures. *Surface Science* **287**, 79-83 (1993).

17 Tabatabaei, J., Sakakini, B., Watson, M. & Waugh, K. The detailed kinetics of the adsorption of hydrogen on polycrystalline copper studied by reactive frontal chromatography. *Catalysis letters* **59**, 151-155 (1999).

18 Ward, A. The sorption of hydrogen on copper. Part I.—Adsorption and the heat of adsorption. *Proceedings of the Royal Society of London. Series A, Containing Papers of a Mathematical and Physical Character* **133**, 506-522 (1931).

19 Beebe, R. A. The heats of adsorption of hydrogen and carbon monoxide on copper. *Transactions of the Faraday Society* **28**, 761-765 (1932).

20 Beebe, R. A., Low Jr, G. W., Wildner, E. L. & Goldwasser, S. The Adsorption of Hydrogen and Deuterium on Copper at Low Pressures. *Journal of the American Chemical Society* **57**, 2527-2532 (1935).

21 Shield, L. S. & Russell, W. W. CALORIMETRIC HEATS OF ADSORPTION FOR HYDROGEN ON NICKEL, COPPER AND SOME OF THEIR ALLOYS. *The Journal of Physical Chemistry* **64**, 1592-1594 (1960).

22 Holden, S. & Rossington, D. Hydrogen adsorption on copper: Studies of parahydrogen conversion. *Journal of Catalysis* **4**, 403-405 (1965).

23 Reisfeld, G., Shaltiel, D. & Steinberger, I. The influence of hydrogen at pressures up to 2000 Pa on the work function of Cu (111). *Journal of Physics: Condensed Matter* **7**, 2687 (1995).

24 Cao, K., Füchsel, G., Kleyn, A. W. & Juurlink, L. B. Hydrogen adsorption and desorption from Cu(111) and Cu(211). *Physical Chemistry Chemical Physics* **20**, 22477-22488 (2018).

25 Wei, Z., Martirez, J. M. P. & Carter, E. A. Introducing the embedded random phase approximation: H_2 dissociative adsorption on Cu(111) as an exemplar. *The Journal of Chemical Physics* **159**, 194108 (2023).

26 Hammer, B., Scheffler, M., Jacobsen, K. W. & Nørskov, J. K. Multidimensional potential energy surface for H_2 dissociation over Cu(111). *Physical Review Letters* **73**, 1400 (1994).

27 Díaz, C. *et al.* Chemically accurate simulation of a prototypical surface reaction: H_2 dissociation on Cu (111). *Science* **326**, 832-834 (2009).

28 Nattino, F., Díaz, C., Jackson, B. & Kroes, G.-J. Effect of Surface Motion on the Rotational Quadrupole Alignment Parameter of D_2 Reacting on Cu(111). *Physical Review Letters* **108**, 236104 (2012).

29 Tchakoua, T., Gerrits, N., Smeets, E. & Kroes, G.-J. SBH17: Benchmark database of barrier heights for dissociative chemisorption on transition metal surfaces. *Journal of Chemical Theory and Computation* **19**, 245-270 (2023).

30 Tchakoua, T. *et al.* Constructing Mixed Density Functionals for Describing Dissociative Chemisorption on Metal Surfaces: Basic Principles. *The Journal of Physical Chemistry A* **127**, 10481-10498 (2023).

31 Doblhoff-Dier, K., Meyer, J., Hoggan, P. E. & Kroes, G.-J. Quantum Monte Carlo Calculations on a Benchmark Molecule–Metal Surface Reaction: H2 + Cu(111). *Journal of Chemical Theory and Computation* **13**, 3208-3219 (2017).

32 Mudiyanselage, K. *et al.* Adsorption of hydrogen on the surface and sub-surface of Cu(111). *The Journal of Chemical Physics* **139**, 044712 (2013).

33 Sakong, S. & Groß, A. Dissociative adsorption of hydrogen on strained Cu surfaces. *Surface Science* **525**, 107-118 (2003).

34 Kaufmann, S., Shuai, Q., Auerbach, D. J., Schwarzer, D. & Wodtke, A. M. Associative desorption of hydrogen isotopologues from copper surfaces: Characterization of two reaction mechanisms. *The Journal of Chemical Physics* **148**, 194703 (2018).

35 Michelsen, H., Rettner, C. & Auerbach, D. On the influence of surface temperature on adsorption and desorption in the D_2/Cu(111) system. *Surface Science* **272**, 65-72 (1992).

36 Rettner, C., Auerbach, D. & Michelsen, H. Role of vibrational and translational energy in the activated dissociative adsorption of D_2 on Cu(111). *Physical Review Letters* **68**, 1164 (1992).

37 Michelsen, H., Rettner, C., Auerbach, D. & Zare, R. Effect of rotation on the translational and vibrational energy dependence of the dissociative adsorption of D_2 on Cu(111). *The Journal of Chemical Physics* **98**, 8294-8307 (1993).

38 Rettner, C., DeLouise, L. & Auerbach, D. Effect of incidence kinetic energy and surface coverage on the dissociative chemisorption of oxygen on W (110). *The Journal of Chemical Physics* **85**, 1131-1149 (1986).

39 Michelsen, H. & Auerbach, D. A critical examination of data on the dissociative adsorption and associative desorption of hydrogen at copper surfaces. *The Journal of Chemical Physics* **94**, 7502-7520 (1991).

40 Nattino, F. *et al.* Dissociation and recombination of D_2 on Cu(111): Ab initio molecular dynamics calculations and improved analysis of desorption experiments. *The Journal of Chemical Physics* **141**, 124705 (2014).

41 Dorenkamp, Y. *et al.* Hydrogen collisions with transition metal surfaces: Universal electronically nonadiabatic adsorption. *The Journal of Chemical Physics* **148**, 034706 (2018).

42 Auerbach, D. J. personal communication. (2023).

43 Chase, M. W. *NIST-JANAF thermochemical tables*. Vol. 9 (American Chemical Society, 1998).

44 Murphy, M. & Hodgson, A. Adsorption and desorption dynamics of H_2 and D_2 on Cu(111): The role of surface temperature and evidence for corrugation of the dissociation barrier. *The Journal of Chemical Physics* **108**, 4199-4211 (1998).

45 Rettner, C., Michelsen, H., Auerbach, D. & Mullins, C. Dynamics of recombinative desorption: Angular distributions of H_2, HD, and D_2 desorbing from Cu(111). *The Journal of Chemical Physics* **94**, 7499-7501 (1991).

46 Linstrom, P. J. & Mallard, W. G. (National Institute of Standards and Technology, Gaithersburg MD, 20899, https://doi.org/10.18434/T4D303, retrieved March 8, 2024).

47 Straumanis, M. & Yu, L. Lattice parameters, densities, expansion coefficients and perfection of structure of Cu and of Cu–In α phase. *Acta Crystallographica Section A: Crystal Physics, Diffraction, Theoretical and General Crystallography* **25**, 676-682 (1969).

48 Galparsoro, O., Kaufmann, S., Auerbach, D. J., Kandratsenka, A. & Wodtke, A. M. First principles rates for surface chemistry employing exact transition state theory: application to recombinative desorption of hydrogen from Cu(111). *Physical Chemistry Chemical Physics* **22**, 17532-17539 (2020).

49 Garrett, B. C. & Truhlar, D. G. Semiclassical tunneling calculations. *Journal of Physical Chemistry* **83**, 2921-2926 (1979).

50 Truhlar, D. G. & Kuppermann, A. Exact tunneling calculations. *Journal of the American Chemical Society* **93**, 1840-1851 (1971).

51 Eckart, C. The penetration of a potential barrier by electrons. *Physical Review* **35**, 1303 (1930).

52 Johnston, H. S. & Heicklen, J. Tunnelling corrections for unsymmetrical Eckart potential energy barriers. *The Journal of Physical Chemistry* **66**, 532-533 (1962).

53 Brown, R. L. A Method of Calculating Tunneling Corrections for Eckart Potential Barriers. *JOURNAL OF RESEARCH of the National Bureau of Standards* **86** (1981).

54 Truhlar, D. G. *et al.* The Incorporation of Quantum Effects in Enzyme Kinetics Modeling. *Accounts of Chemical Research* **35**, 341-349 (2002).

55 Fernandez-Ramos, A. & Truhlar, D. G. Improved algorithm for corner-cutting tunneling calculations. *The Journal of Chemical Physics* **114**, 1491-1496 (2001).

56 Kresse, G. & Furthmuller, J. Efficiency of ab initio total energy calculations for metals and semiconductors using plane wave basis set. *Computational Materials Science* **6**, 15-50 (1996).

57 Kresse, G. & Furthmüller, J. Efficient iterative schemes for ab initio total-energy calculations using a plane-wave basis set. *Physical Review B* **54**, 11169-11186 (1996).

58 Blöchl, P. E. Projector augmented-wave method. *Physical Review B* **50**, 17953-17979 (1994).

59 Madsen, G. K. H. Functional form of the generalized gradient approximation for exchange: The PBEα functional. *Physical Review B* **75**, 195108 (2007).

60 Dion, M., Rydberg, H., Schröder, E., Langreth, D. C. & Lundqvist, B. I. Van der Waals Density Functional for General Geometries. *Physical Review Letters* **92**, 246401 (2004).

61 Jiang, B. & Guo, H. Permutation invariant polynomial neural network approach to fitting potential energy surfaces. III. Molecule-surface interactions. *The Journal of Chemical Physics* **141**, 034109 (2014).

62 Jiang, B. & Guo, H. Permutation invariant polynomial neural network approach to fitting potential energy surfaces. *The Journal of Chemical Physics* **139**, 054112 (2013).

63 Lin, Q., Zhang, L., Zhang, Y. & Jiang, B. Searching configurations in uncertainty space: Active learning of high-dimensional neural network reactive potentials. *Journal of Chemical Theory and Computation* **17**, 2691-2701 (2021).

64 Lin, Q., Zhang, Y., Zhao, B. & Jiang, B. Automatically growing global reactive neural network potential energy surfaces: A trajectory-free active learning strategy. *The Journal of Chemical Physics* **152**, 154104 (2020).

65 Suleimanov, Y. V., Aoiz, F. J. & Guo, H. Chemical Reaction Rate Coefficients from Ring Polymer Molecular Dynamics: Theory and Practical Applications. *The Journal of Physical Chemistry A* **120**, 8488-8502 (2016).

66 Suleimanov, Y. V., Allen, J. W. & Green, W. H. RPMDrate: bimolecular chemical reaction rates from ring polymer molecular dynamics. *Computer Physics Communications* **184**, 833-840 (2013).

67 Habershon, S., Manolopoulos, D. E., Markland, T. E. & III, T. F. M. Ring-Polymer Molecular Dynamics: Quantum Effects in Chemical Dynamics from Classical Trajectories in an Extended Phase Space. *Annual Review of Physical Chemistry* **64**, 387-413 (2013).

68 Zhang, L., Zuo, J., Suleimanov, Y. V. & Guo, H. Ring Polymer Molecular Dynamics Approach to Quantum Dissociative Chemisorption Rates. *The Journal of Physical Chemistry Letters* **14**, 7118-7125 (2023).

69 Suleimanov, Y. V., Collepardo-Guevara, R. & Manolopoulos, D. E. Bimolecular reaction rates from ring polymer molecular dynamics: Application to $H + CH_4 \rightarrow H_2 + CH_3$. *The Journal of Chemical Physics* **134**, 044131 (2011).

70 Collepardo-Guevara, R., Suleimanov, Y. V. & Manolopoulos, D. E. Bimolecular reaction rates from ring polymer molecular dynamics. *The Journal of Chemical Physics* **130**, 174713 (2009).

71 Bennett, C. H. in *Algorithms for Chemical Computations, ACS Symposium Series* Vol. 46 (ed R. E. Christofferson) (ACS, 1977).

72 Chandler, D. Statistical mechanics of isomerization dynamics in liquids and the transition state approximation. *The Journal of Chemical Physics* **68**, 2959-2970 (1978).

73 Frenkel, D. & Smit, B. *Understanding Molecular Simulation, Second Edition: From Algorithms to Applications* (Academic Press, 2002).

74 Craig, I. R. & Manolopoulos, D. E. A refined ring polymer molecular dynamics theory of chemical reaction rates. *The Journal of Chemical Physics* **123**, 034102 (2005).

75 Kästner, J. & Thiel, W. Analysis of the statistical error in umbrella sampling simulations by umbrella integration. *The Journal of Chemical Physics* **124**, 234106 (2006).

76 Kästner, J. & Thiel, W. Bridging the gas between thermodynamic integration and umbrella sampling provides a novel analysis method: "umbrella integration". *The Journal of Chemical Physics* **123**, 144104 (2005).

77 Stark, W. G. *et al.* Benchmarking of machine learning interatomic potentials for reactive hydrogen dynamics at metal surfaces. *Machine Learning: Science and Technology* **5**, 030501 (2024).

78 Andersen, H. C. Molecular dynamics simulations at constant pressure and/or temperature. *The Journal of Chemical Physics* **72**, 2384-2393 (1980).

79 Ryckaert, J. P., Ciccotti, G. & Berendsen, H. J. Numerical integration of the cartesian equations of motion of a system with constraints: molecular dynamics of n-alkanes. *Journal of Computational Physics* **23**, 327-341 (1977).

80 Takayangi, K. The production of rotational and vibrational transitions in encounters between molecules. *Advances in Atomic and Molecular Physics* **1**, 149-194 (1965).

81 Gilson, M. K. & Irikura, K. K. Symmetry numbers for rigid, flexible, and fluxional molecules: theory and applications. *J Phys Chem B* **114**, 16304-16317 (2010).

82 Borodin, D. *et al.* Quantum effects in thermal reaction rates at metal surfaces. *Science* **377**, 394-398 (2022).

83 Miller, W. H., Schwartz, S. D. & Tromp, J. W. Quantum mechanical rate constants for bimolecular reactions. *The Journal of Chemical Physics* **79**, 4889-4899 (1983).

84 Schenter, G. K., Garrett, B. C. & Truhlar, D. G. Generalized transition state theory in terms of the potential of mean force. *The Journal of Chemical Physics* **119**, 5828-5833 (2003).

85 Sprowl, L. H., Campbell, C. T. & Arnadottir, L. Hindered translator and hindered rotor models for adsorbates: Partition functions and entropies. *The Journal of Physical Chemistry C* **120**, 9719-9731 (2016).

86 Jørgensen, M. & Gronbeck, H. Adsorbate entropies with complete potential energy sampling in microkinetic modeling. *The Journal of Physical Chemistry C* **121**, 7199-7207 (2017).

87 Waitt, C., Miles, A. R. & Schneider, W. F. Adsorbate free energies from DFT-derived translational energy landscapes. *The Journal of Physical Chemistry C* **125**, 20331-20342 (2021).

88 Schwarzer, M. *et al.* Adsorption and Absorption Energies of Hydrogen with Palladium. *The Journal of Physical Chemistry C* **126**, 14500-14508 (2022).

89 Borodin, D. *et al.* Steric Hindrance of NH_3 Diffusion on Pt(111) by Co-Adsorbed O-Atoms. *Journal of the American Chemical Society* **144**, 21791-21799 (2022).

90 Tully, J. C. The dynamics of adsorption and desorption. *Surface Science* **299**, 667-677 (1994).

91 Anger, G., Winkler, A. & Rendulic, K. Adsorption and desorption kinetics in the systems H_2/Cu (111), H_2/Cu(110) and H_2/Cu(100). *Surface Science* **220**, 1-17 (1989).

92 Kammler, T. & Küppers, J. Interaction of H atoms with Cu(111) surfaces: Adsorption, absorption, and abstraction. *The Journal of Chemical Physics* **111**, 8115-8123 (1999).

93 Winkler, A. Absolute calibration of small gas amounts. *Journal of Vacuum Science & Technology A: Vacuum, Surfaces, and Films* **5**, 2430-2435 (1987).

94 Rettner, C. & Auerbach, D. Quantum-state distributions for the HD product of the direct reaction of H (D)/Cu (111) with D (H) incident from the gas phase. *The Journal of Chemical Physics* **104**, 2732-2739 (1996).

95 Balakrishnan, A., Vallet, M. & Stoicheff, B. Dissociation energy of the HD molecule. *Journal of Molecular Spectroscopy* **162**, 168-171 (1993).

96 Irikura, K. K. Experimental vibrational zero-point energies: Diatomic molecules. *Journal of physical and chemical reference data* **36**, 389-397 (2007).

97 Kresse, G. & Hafner, J. Ab initio molecular dynamics for liquid metals. *Physical Review B* **47**, 558-561 (1993).

98 Kresse, G. & Hafner, J. Ab initio molecular-dynamics simulation of the liquid-metal--amorphous-semiconductor transition in germanium. *Physical Review B* **49**, 14251-14269 (1994).

99 Monkhorst, H. J. & Pack, J. D. Special points for Brillouin-zone integrations. *Physical Review B* **13**, 5188-5192 (1976).

100 Methfessel, M. & Paxton, A. T. High-precision sampling for Brillouin-zone integration in metals. *Physical Review B* **40**, 3616-3621 (1989).

101 Perdew, J. P., Burke, K. & Ernzerhof, M. Generalized Gradient Approximation Made Simple. *Physical Review Letters* **77**, 3865-3868 (1996).

102 Hammer, B., Hansen, L. B. & Nørskov, J. K. Improved adsorption energetics within density-functional theory using revised Perdew-Burke-Ernzerhof functionals. *Physical Review B* **59**, 7413-7421 (1999).

103 Tkatchenko, A. & Scheffler, M. Accurate Molecular Van Der Waals Interactions from Ground-State Electron Density and Free-Atom Reference Data. *Physical Review Letters* **102**, 073005 (2009).

104 Klimeš, J., Bowler, D. R. & Michaelides, A. Van der Waals density functionals applied to solids. *Physical Review B* **83**, 195131 (2011).

105 Lee, K., Murray, É. D., Kong, L., Lundqvist, B. I. & Langreth, D. C. Higher-accuracy van der Waals density functional. *Physical Review B* **82**, 081101 (2010).

106 Chuang, Y.-Y., Radhakrishnan, M. L., Fast, P. L., Cramer, C. J. & Truhlar, D. G. Direct Dynamics for Free Radical Kinetics in Solution: Solvent Effect on the Rate Constant for the Reaction of Methanol with Atomic Hydrogen. *The Journal of Physical Chemistry A* **103**, 4893-4909 (1999).

107 Henkelman, G., Uberuaga, B. P. & Jónsson, H. A climbing image nudged elastic band method for finding saddle points and minimum energy paths. *The Journal of Chemical Physics* **113**, 9901-9904 (2000).

108 Evans, M. G. & Polanyi, M. Inertia and driving force of chemical reactions. *Transactions of the Faraday Society* **34**, 11-24 (1938).

109 Pallassana, V. & Neurock, M. Electronic Factors Governing Ethylene Hydrogenation and Dehydrogenation Activity of Pseudomorphic PdML/Re(0001), PdML/Ru(0001), Pd(111), and PdML/Au(111) Surfaces. *Journal of Catalysis* **191**, 301-317 (2000).

110 Liu, Z.-P. & Hu, P. General trends in CO dissociation on transition metal surfaces. *The Journal of Chemical Physics* **114**, 8244-8247 (2001).

111 Logadottir, A. *et al.* The Brønsted–Evans–Polanyi Relation and the Volcano Plot for Ammonia Synthesis over Transition Metal Catalysts. *Journal of Catalysis* **197**, 229-231 (2001).

112 Nørskov, J. K. *et al.* Universality in Heterogeneous Catalysis. *Journal of Catalysis* **209**, 275-278 (2002).

113 Bligaard, T. *et al.* The Brønsted–Evans–Polanyi relation and the volcano curve in heterogeneous catalysis. *Journal of Catalysis* **224**, 206-217 (2004).
